# Supplementary material for: Network models of driver behavior
Source: PeerJ. 2019 Jan 10;6:e6119. doi: 10.7717/peerj.6119 (PMC6330205; doi:10.7717/peerj.6119)
Supplement: Supplemental Information 1 [file peerj-07-6119-s001.docx]

# Network models of driver behavior: supplementary materials

Markus Mattsson

University of Helsinki, Department of Psychology and Logopedics

# Supplementary material

**Correlations and adjacency matrices.**

**Supplementary analyses.**

**Edge weight accuracy and centrality stability.**

**Choosing the background variables.**

## Correlations and adjacency matrices

The raw correlations between the variables are shown in Supplementary tables S1 (between-person model) and S3 (cross-sectional model), while the adjacency matrices are shown in tables S2 (between-person model) and S4 (cross-sectional model). The correlations in table S1 are polychoric correlations, while those in table S3 are Pearson correlations. In Supplementary tables S3-S4, the DBQ variables are followed by the two attitude variables, two self-image variables and five variables related to self-perceived improvement needs.

------------------ Supplementary tables S1-S4 here ------------------

## Choosing the background variables

There were no principled theoretical reasons to include particular background variables in the cross-sectional model. For this reason, the choice of background variables was performed in the exploratory mode of analysis by constructing three network models for examining the associations of DBQ variables with variables related to the drivers’ attitudes, self-perceived improvement needs and self-image as drivers. The background variables that had the strongest partial correlations with the DBQ variables were included in the cross-sectional network model. First, the model of attitudes and DBQ variables shows that nodes a1 (“Decreasing motorway speed limits is a good idea”) and a5 (“Cars should never overtake on inside lane”) had the strongest partial correlations with driver behaviours. Most of the attitude variables shown in Supplementary figure S1 were related to speeding, and due to this overlap in content, it was understandable that not all of them shared strong edges with variables related to driving behaviour. Nodes a1 and a5 that were included in the models reported in the main text represent attitudes toward different components of driving.


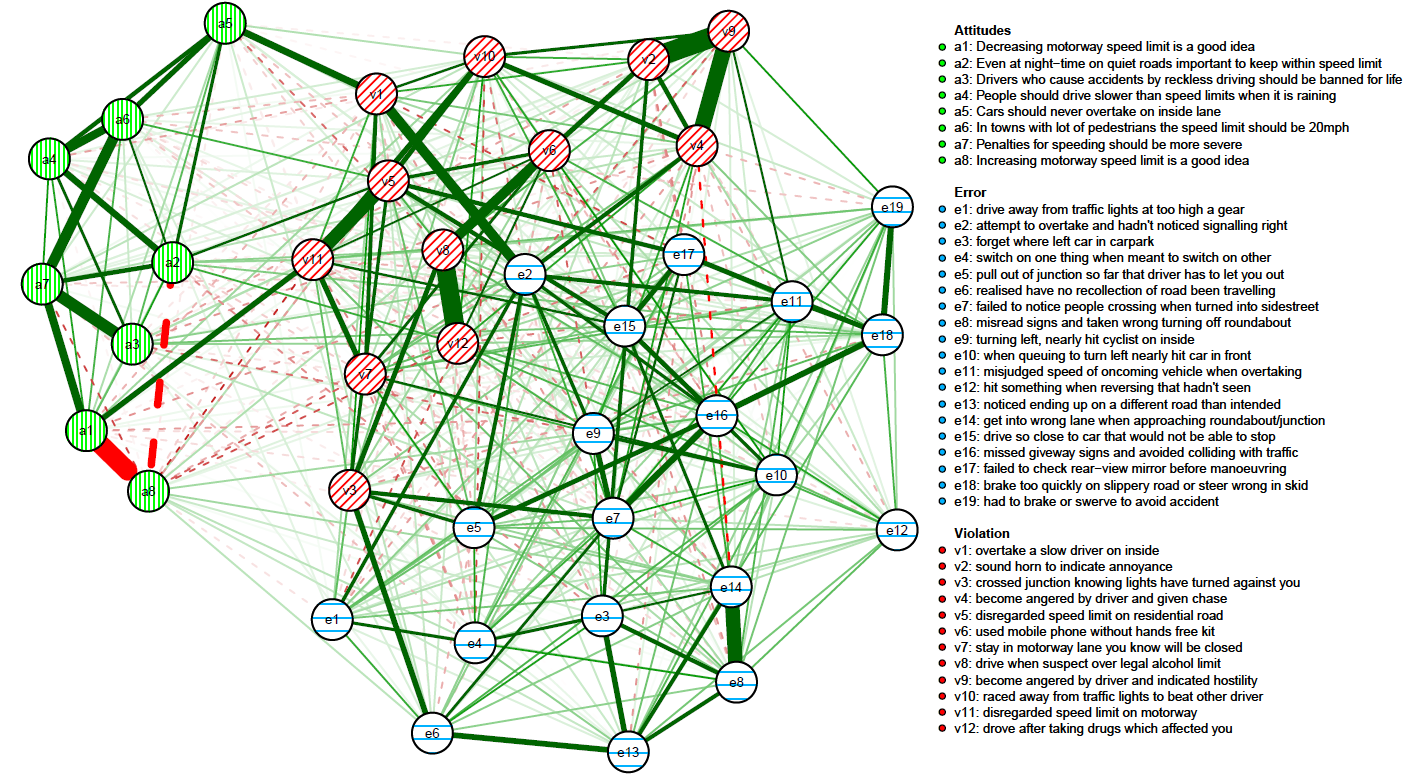


Supplementary figure S1 Network model of attitudes and driver behaviours

The network model of self-perceived improvement needs and driver behaviours (Supplementary figure S2) shows how thematically related perceived improvement needs were inter-related: for instance, nodes i18 and i17 that involve driving in different types of circumstances were strongly associated; similarly, nodes i15 and i14, related to driving on different types of roads shared a strong partial correlation. The nodes that shared the strongest edges with the DBQ nodes were included in the cross-sectional model. These include nodes i1 (“improve controlling the car”) i4 (“improve parking”), i7 (“improve on spotting hazards”), i13 (“improve on changing lanes”) and i20 (“improve on knowing what speed is safe”).


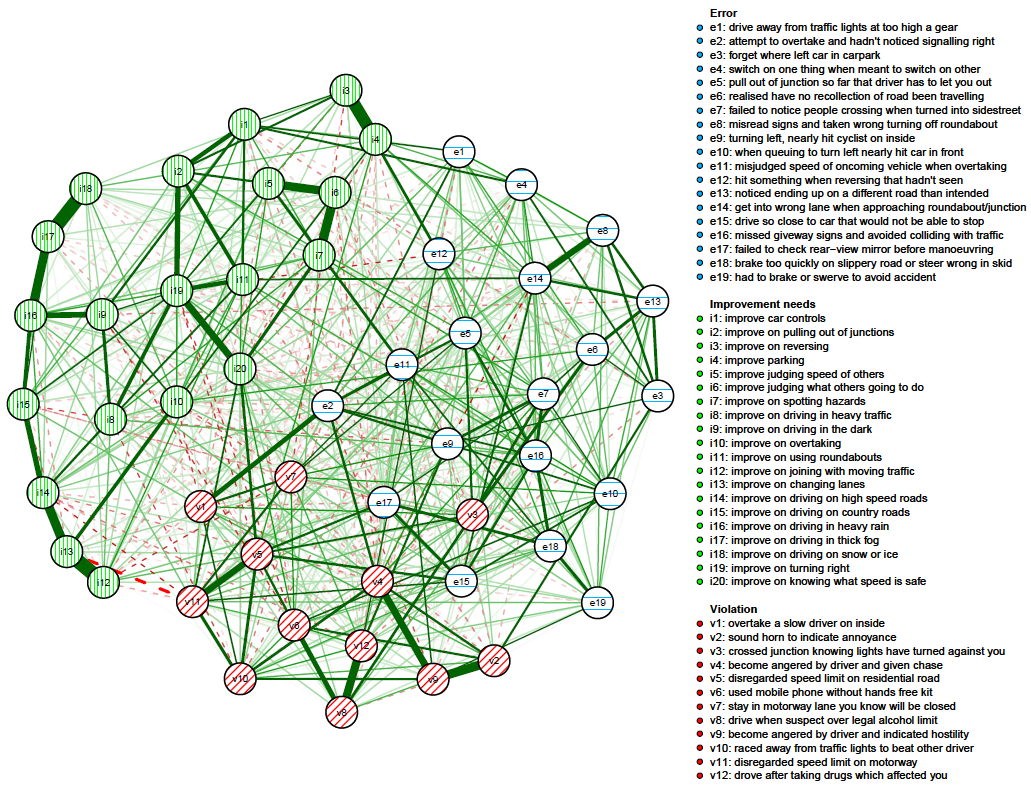


Supplementary figure S2 Network model of self-perceived improvement needs and driver behaviours

Finally, looking at the self-image variables and driver behaviours (Supplementary figure S3), it is immediately obvious that most of the self-image nodes shared no direct edges with the driver behaviour nodes. The self-image variables related to similar aspects of self-image were connected by strong edges: for instance, perceiving oneself as safe (si15) shared a strong edge with perceiving oneself as responsible (si14). The two nodes with the strongest links with the driving behaviours, s11 (perceiving oneself as irritable vs. placid) and s17 (perceiving oneself as slow vs. fast) were included in the cross-sectional network model.


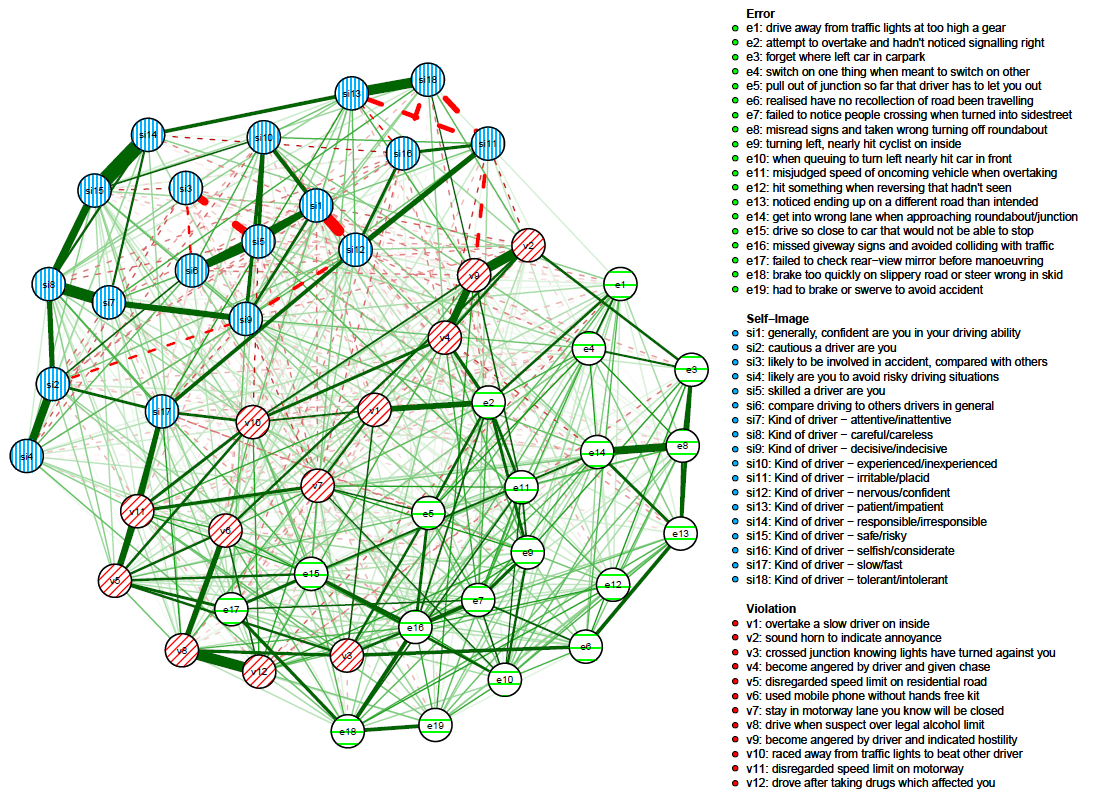


*Supplementary figure S3 Network model of self-image and driver behaviours*

## Accuracy of edge weight estimates and stability of centrality coefficients

The bootstrap analyses reported here enable assessing the accuracy of the edge weight estimates and whether the results are stable with regard to changes in the composition of the sample. The accuracy of the edge weight estimates was assessed by forming 95 % confidence intervals around the estimates using the non-parametric bootstrap procedure described by Epskamp et al^1^. Further, the stability of the estimates of centrality indices was assessed by performing a case-drop bootstrap analysis that involves calculating correlations between the initial estimates and those in the reduced samples that are obtained by dropping a certain percentage of cases from the analysis^1^. Correlation stability coefficients (CS-coefficients) for the three centrality indices are also reported (Supplementary table 5). The CS-coefficient represents the maximum proportion of cases that can be dropped such that the correlation between the original estimates and those based on the bootstrapped samples are 0.7 or more with a 95 % probability. Other values for the correlation and the probability could naturally be chosen, but these values are recommended by the authors of the bootstrap methods. According to a simulation, the values of the CS-coefficients should be above 0.25, and preferably above 0.5^1^.

The values of the CS-coefficient (Supplementary table S5) indicate that the Closeness and Strength indices can be interpreted for the between-person network model (Manuscript Figure 3), while the values of all the centrality indices can be interpreted for the cross-sectional model (Manuscript Figure 5).

Supplementary table S5 CS-coefficients

|  | Between-person network | Cross-sectional network |
| --- | --- | --- |
| Betweenness | 0.205 | 0.517 |
| Closeness | 0.594 | 0.594 |
| Strength | 0.673 | 0.750 |


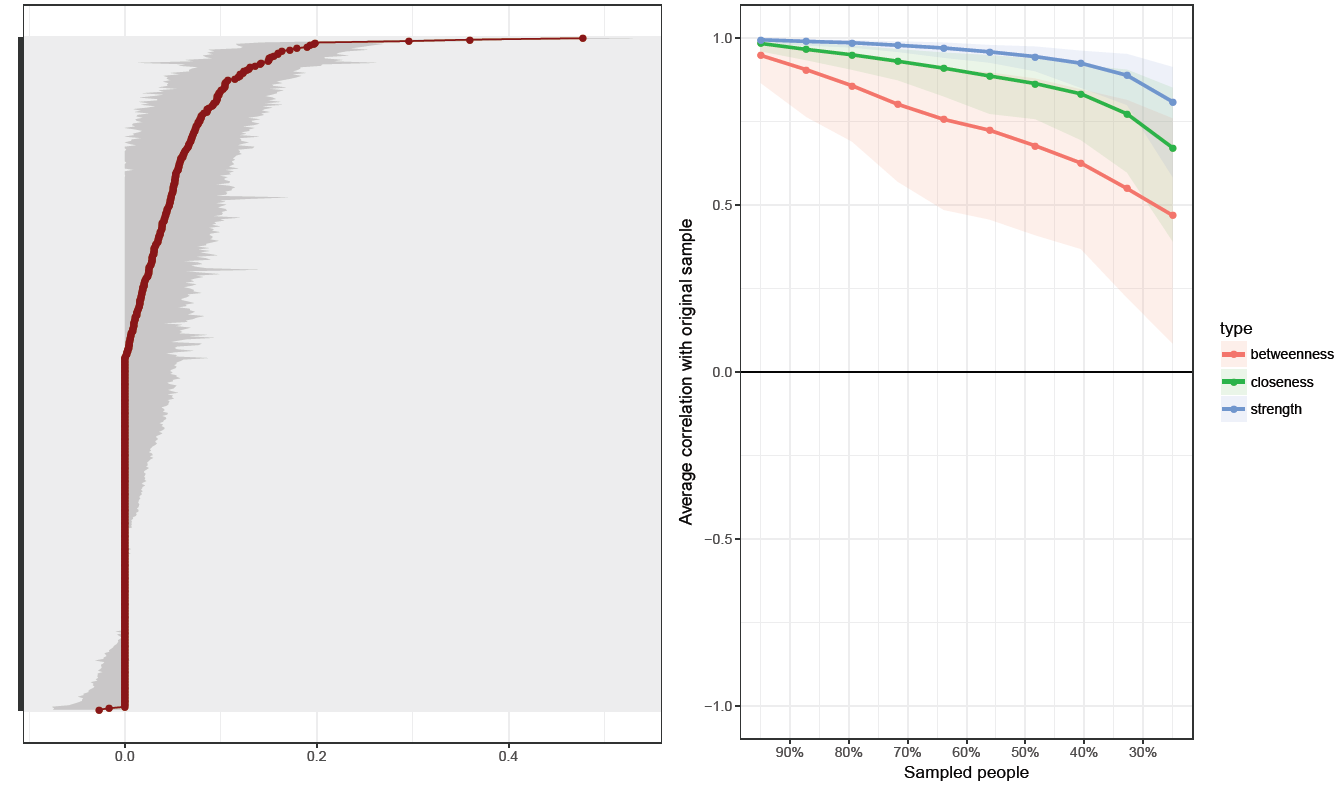
The edge weight estimates for the between-person model were quite accurate even though some of the mid-size estimates may not reliably differ from one another (Supplementary Figure S4, left panel). Closeness and strength centrality indices were quite robust in the face of changes in the composition of the sample (Supplementary Figure S4, right panel).

Supplementary figure S4 Edge weight accuracy (left) and centrality index stability (right) for the between-person network. The edge labels are omitted from the y-axis of the accuracy plot for the sake of clarity. The red dots indicate point estimates for edge strength, while the grey lines show bootstrapped 95 % confidence intervals. The stability plot (right) shows the average correlation of the centrality indices with the sample values as a function of the percentage of sample size included.

Looking at the cross-sectional model, the accuracy of the edge weight estimates was excellent, as indicated by the narrow confidence intervals of Supplementary Figure S5, left panel. The values of all centrality indices were robust to changes in the composition of the sample (Supplementary Figure S5, right panel).


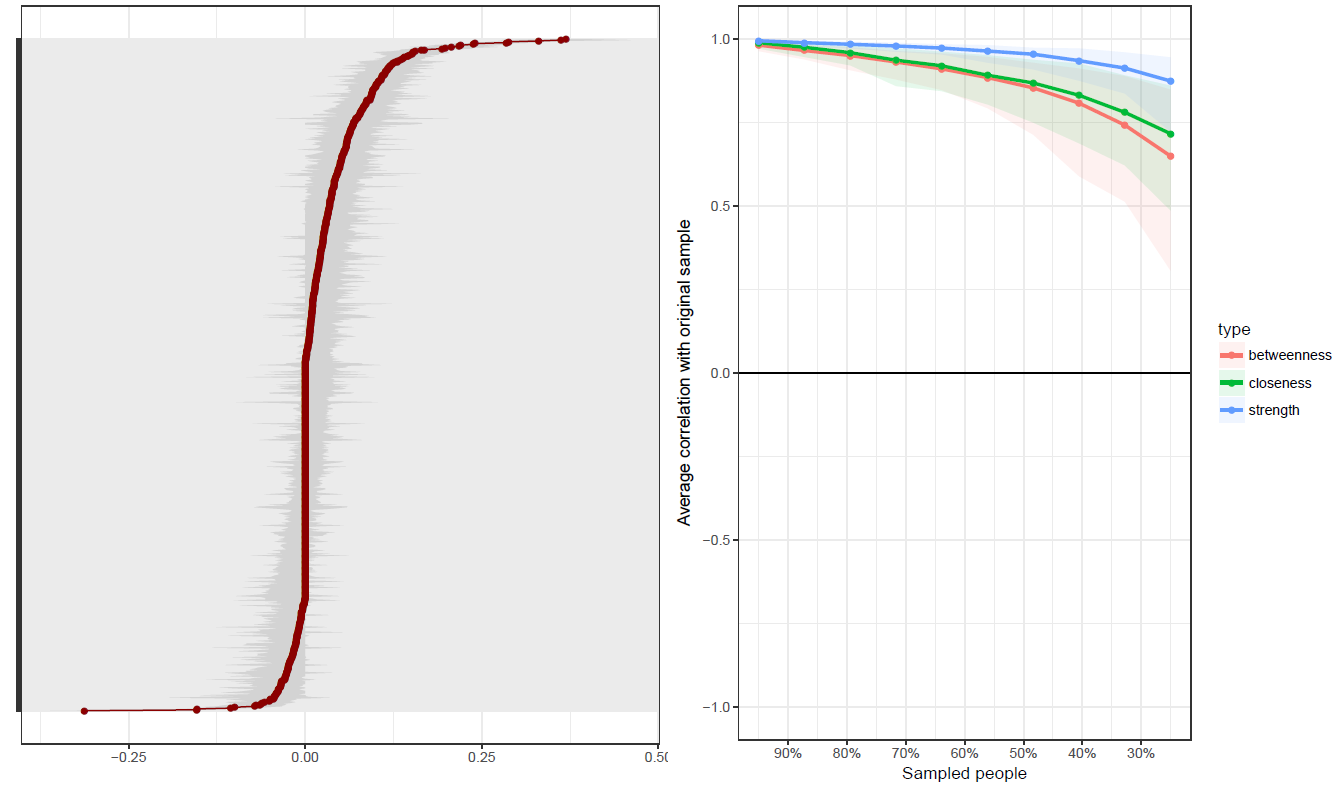


Supplementary figure S5 Edge weight accuracy (left) and centrality stability (right) for the cross-sectional network model

## R syntax files and other additional information

All analysis syntax files, together with the preprint version of the manuscript, can be found at a stable address on the server of the Open Science Framework at <https://osf.io/4wbmt/>

## Reference

1. Epskamp, S., Borsboom, D. & Fried, E. Estimating psychological networks and their accuracy: A tutorial paper. *Behav. Res. Methods*, 1-18 (2017).
