# Supplementary material for: Network models of driver behavior
Source: PeerJ. 2019 Jan 10;6:e6119. doi: 10.7717/peerj.6119 (PMC6330205; doi:10.7717/peerj.6119)
Supplement: Supplemental Information 2 [file peerj-07-6119-s002.pdf]

Cohort II study of novice drivers: archived data

TRL Limited

April 2008

Contents

1 Introduction **1**

2 .Questionnaire data **2**

2.1 Available data 2
2.2 Variable names 2
2.3 Derived variables 3
2.4 Links between data sets 3
2.5 Missing and n/a values 3
2.6 Weighting 6
2.7 Accident involvement 6
2.8 Analysis 6

3 Additional data **6**

3.1 Driving test report (DL25) 6
3.2 Theory Test 8

4 References **8**

1 Introduction

The cohort II study of learner and novice drivers was a major six-year study, funded by the Department for Transport, providing an up-to-date picture of how 'cohorts' of learner drivers in Great Britain undertake driver training and testing, and of their subsequent experiences as new drivers. Findings from the study are reported in Wells et al, 2008.

This data collated as part of the project has been made available on the UK data archive. This document describes the data available and some key information about the data. All users of the data should read this document.

Every three months, from November 2001 to August 2005, a random sample of 8,000 practical test candidates was drawn by the Driving Standards Agency (DSA) from candidates in a given week (this was approximately one-third of those taking their test in that week). For the purposes of the study, the resulting 16 cohorts were labelled A to P. 'Learning to driver questionnaires' (LTDQ) were sent to these candidates and, if they passed their test, follow-up 'driving experience questionnaires' (DEQ) were sent 6 months (DEQ1), 12 months (DEQ2), 24 months (DEQ3) and 36 months (DEQ4) after passing the test. Due to the timescale of the project, not all of the cohorts received all four DEQs.

Table 1 shows the test dates of the practical driving test for each cohort and the range of ID numbers. The table also shows which cohorts received each questionnaire.

Table 1: Test dates and questionnaires available for each cohort

cohort	cohort num	Test date (week commencing)	ID from	ID to	LTDQ	DL25*	TT*	DEQ1	DEQ2	DEQ3	DEQ4
A	1	04/11/2001	1	8000	y	y	y	y	y	y	y
B	2	04/02/2002	8001	16000	y	y	y	y	y	y	y
C	3	29/04/2002	16001	24000	y	y	y	y	y	y	y
D	4	05/08/2002	24001	32000	y	y	y	y	y	y	y
E	5	04/11/2002	32001	40000	y	y	y	y	y	y	y
F	6	03/02/2003	40001	48000	y	y	y	y	y	y	y
G	7	28/04/2003	48001	56000	y	y	y	y	y	y	
H	8	04/08/2003	56001	64000	y	y	y	y	y	y	
I	9	03/11/2003	64001	72000	y	y	y	y	y	y	
J	10	02/02/2004	72001	80000	y	y	y	y	y	y	
K	11	26/04/2004	80001	88000	y	y	y	y	y		
L	12	02/08/2004	88001	96000	y	y	y	y	y		
M	13	01/11/2004	98001	104000	y	y	y	y	y		
N	14	31/01/2005	104001	112000	y	y	y	y	y		
O	15	09/05/2005	112001	120000	y	y	y	y			
P	16	08/08/2005	120001	128000	y	y	y	y			

*DL25 (Driving test report) and TT (Theory test scores) not available for all respondents. TT only available for tests after November 2002. Some respondents have multiple theory test scores.

Table 2 shows the questionnaire data and other data included in the data archive. The data consist of data from 68 questionnaires, each including over 200 variables. Additional data from the practical driving test report (DL25) and the theory test (TT) was supplied by the Driving Standards Agency (DSA) and is also included.

Table 2: Number of variables and responses for each data set

Dataset	Type	Cohorts	Number of variables (including agreed derived variables)	Total number of records
LTDQ	Questionnaire	A-P	204	42,854
DL25*	Driving test report	A-P	183	39,270
TT*	Theory test score	A-L	5	105,086
DEQ1	Questionnaire	A-P	234	10,064
DEQ2	Questionnaire	A-P	230	7,4187
DEQ3	Questionnaire	A-L	232	4,187
DEQ4	Questionnaire	A-H	232	2,760
Total			1,320	281,992
*DL25 and TT not available for all respondents. TT only available for tests after November 2002. Some respondents have multiple theory test scores				

2 Questionnaire data

2.1 Available data

Sample questionnaires are included on the archive.

The data sets available on the archive are shown in the above table. All of the cohorts have been combined into one data set for each of the questionnaires.

The data is in SPSS data (sav) format, and includes variable and value labels.

2.2 Variable names

There were several complex issues with this large data set

- All questionnaires were labelled with questions A1, A2, B1 etc
- Questions on the DEQs were identical apart from the timescale
- Analysis was required across questionnaires and across cohorts
- There were minor changes to the LTDQ questionnaire for some cohorts
- SPSS files had a limit of 8 characters for the variable name (albeit now increased to 64)

Therefore, a system was needed in order to store the data and allow easy retrieval of data and to link it through to SPSS files with appropriate labels. Each question in each questionnaire was given

- a variable ID (VID) – the same VID was used within different LTDQs provided that the question was still the same, i.e. in LTDQ A and B question 'B11a' was V00347 but in LTDQ C to P question 'B11a' had VID of V00455 because of a re-

wording. However a unique VID was required for all the DEQ variables because they related to different time periods.

- a variable label, which was normally the text from the questionnaire
- value labels, which gave the text for numerical values

The variable names spreadsheet which is available with the data gives the links between the question names on each questionnaire and the VIDs. This can be used to look up a VID for a question on a questionnaire or to find the questionnaire(s) and question name for a VID.

2.3 Links between data sets

The questionnaire data, DL25 and TT data can be linked via the ID.

The response rate for each subsequent follow-up questionnaire was generally less than the previous one. Each DEQ was sent to those respondents of the LTDQ who passed their test, whether or not they had responded to the previous DEQ. This means that, for example, there were respondents of DEQ2 who had not responded to DEQ1.

DL25 data and TT scores were not available for all respondents.

Where a respondent took more than one theory test, there is a record in the data for each occurrence, and hence the questionnaire data will link to more than one record in the TT scores data. The final TT score (ie when a candidate finally passed) may not always be available.

2.4 Missing and n/a values

The data from the questionnaires was recoded so that blank entries were split into:

- o Missing (respondents failed to complete this question)
- o N/a (respondent was guided past this question in the questionnaire)

Both of these are recorded as missing values in the SPSS data files and hence do not appear in tables or in calculations.

Note that these missing values mean that each question has a different number of respondents.

2.5 Derived variables

The following derived variables are included in the archived data, and SPSS syntax files are provided which show the calculations:

- Age
    - o Age refers to the age in years when a respondent took the test, and was calculated from the date of birth and week of test.
    - o Two versions of Age group have been derived: AgeGroup1 has single ages from 16 to 24, five-year bands up to 59 and ages 60+. AgeGroup2 has the groups 16-19, 20-24, 25-29, 30-39, 40-49, 50-59 and 60+.
- West attitude to speed scale
    - o This consists of 8 questions each scored on a 5-point Likert scale. The questions ask about level of agreement on speed related issues. A high score=safer attitude.
- Guppy Scale

    - These are three scales relating to driving style as defined by responses to a bi-polar 7-point Likert scale. There were 12 bi-polar scales where respondents were required to indicate what end of each scale they were more like, e.g. attentive or inattentive, or careful – careless etc. The responses were coded onto a 7-point scale. The 3 driver scales were derived by combining 4 of the bi-polar scale values as follows.
    - Driver style1, high score = attentive, careful, responsible, safe
    - Driver style2, high score = placid, patient, considerate, tolerant
    - Driver style3, high score = decisive, experienced, confident, fast
- Hazard awareness scale
    - The scale consisted of 7 questions relating to hazard awareness. Respondents were required to indicate on a 6-point scale how often different things happened to them while driving, e.g. 'have to brake sharply to avoid a collision with the vehicle ahead because it has slowed'.
- DBQ scales
    - These consist of 5-scales based on responses to 34 questions which asked how often something occurred when they were driving. The responses were on 6-point scales from 'never' to 'nearly all the time'. The 5-scales consist of (for high scores):
    - Violations - High score = more violations
    - Errors - High score = more driving errors
    - AgressiveViolations - High score = more aggressive violations
    - Inexperience - High score = more inexperienced
    - Slips - High score = make more slips

Additional derived variables are included as shown in the table below (as well as renamed variables). The associated syntax is provided as part of the archive. This will involve the calculation of some intermediate variables, e.g. an indication of the accident occurring within the appropriate time period. It is not considered necessary to include these intermediate variables; they are only used within the calculation process and have little use for other purposes.

Table 3: Additional derived variables

Source	Variable name	Variable label
LTDQ	mtotest	months to test from starting to drive (all)
	mtotest2	months to test from starting to drive (no gap when learning)
	mttotest	months from theory test to driving test (no gap when learning)
	mtott	months to theory test (no gap & had driven before TT)
	mptot	months from provisional to practical test
	takeHP	took the HP test (0=no or n/a, 1=yes) (derived/recoded variable)
	pass	pass test? (coded as 0=no, 1=yes) (recoded and renamed variable)
DEQ1	accinv1	accident involvement (0-6 months) (coded as 0=no, 1=yes)
	naccs1	total number of stated accidents 0 to 4+ (0-6 months)
	acc1	accs in 0-6 months with date OK (max of 3)
	pracc1	public rd accs in 0-6 months with date OK
	nlacc1	non low speed public rd accs in 0-6 months with date OK
	nlbacc1	non low speed public rd blame accs in 0-6 months with date OK
	acacc1	active public rd accs in 0-6 months with date OK
DEQ2	accinv2	accident involvement (7-12 months) (coded as 0=no, 1=yes)
	naccs2	total number of stated accidents 0 to 4+ (7-12 months)
	acc2	accs in 7-12 months with date OK (max of 3)
	pracc2	public rd accs in 7-12 months with date OK
	nlacc2	non low speed public rd accs in 7-12 months with date OK
	nlbacc2	non low speed public rd blame accs in 7-12 months with date OK
	acacc2	active public rd accs in 7-12 months with date OK
DEQ3	accinv3	accident involvement (13-24 months) (coded as 0=no, 1=yes)
	naccs3	total number of stated accidents 0 to 4+ (13-24 months)
	acc3	accs in 13-24 months with date OK (max of 3)
	pracc3	public rd accs in 13-24 months with date OK
	nlacc3	non low speed public rd accs in 13-24 months with date OK
	nlbacc3	non low speed public rd blame accs in 13-24 months with date OK
	acacc3	active public rd accs in 13-24 months with date OK
DEQ4	accinv4	accident involvement (25-36 months) (coded as 0=no, 1=yes)
	naccs4	total number of stated accidents 0 to 4+ (25-36 months)
	acc4	accs in 25-36 months with date OK (max of 3)
	pracc4	public rd accs in 25-36 months with date OK
	nlacc4	non low speed public rd accs in 25-36 months with date OK
	nlbacc4	non low speed public rd blame accs in 25-36 months with date OK
	acacc4	active public rd accs in 25-36 months with date OK

2.6 Weighting

The final project report for the study (Wells et al, 2008) gives results of comparing some characteristics of respondents with those in the original sample. The characteristics compared were limited to information available from the DSA, namely, age, sex and pass/fail. These could have been used to carry out a weighting exercise, however, other factors which may be unrepresentative of the sample are ethnicity, home language and literacy skills of the sample (as drivers who are unfamiliar with English, or who read with difficulty, are less likely to complete a questionnaire), and data on these was not available. Nevertheless, the actual differences between response groups at the five time-periods were similar on the measures which were available, and the differences between these and the initial sample were relatively small, and therefore basic, unweighted data was used in the analyses in the final report.

2.7 Accident involvement

In the driving experience questionnaires, Question B2 asks the number of accidents which occurred within the period of the questionnaire (last 6 months for DEQ1 and DEQ2, last year for DEQ3 and DEQ4). There are also questions relating to further details for the most recent three accidents, such as type of accident, injuries sustained and what happened, together with the month and year of occurrence.

Analysis of these questions showed that some of the accidents reported by respondents were not within the period of the questionnaire. The accidents have been excluded from the derived variable shown above by comparison of the test date of the cohort with the dates of the reported accidents.

2.8 Analysis

It is important for all users of the data to understand the structure of the data and the interpretation of results.

The process for analysing data was generally as follows:

- Look at the questionnaires to find name of questions that were required
- Use look up table to find the VIDs of those questions
- Check that the VIDs were the same for each cohort required
- Analyse the relevant variables in SPSS

It is important to note that, while associations have been established between many variables in the project report, these are not necessarily causal relationships.

3 Additional data

3.1 Driving test report (DL25)

Data from the driving test report (DL25) is provided for most respondents of the LTDQ. Personal details such as driver number, Approved Driving Instructor (ADI) number and examiner number are not available.

In the SPSS files, the variable labels are given as the item numbers on the form, with m, x and d corresponding to driving faults, serious faults and dangerous faults. For example, 19x corresponds to a serious fault for following distance, and 13am corresponds to a driving fault for moving off safely. A sample DL25 form is shown below.

Figure 1: Driving test report form (DL25)

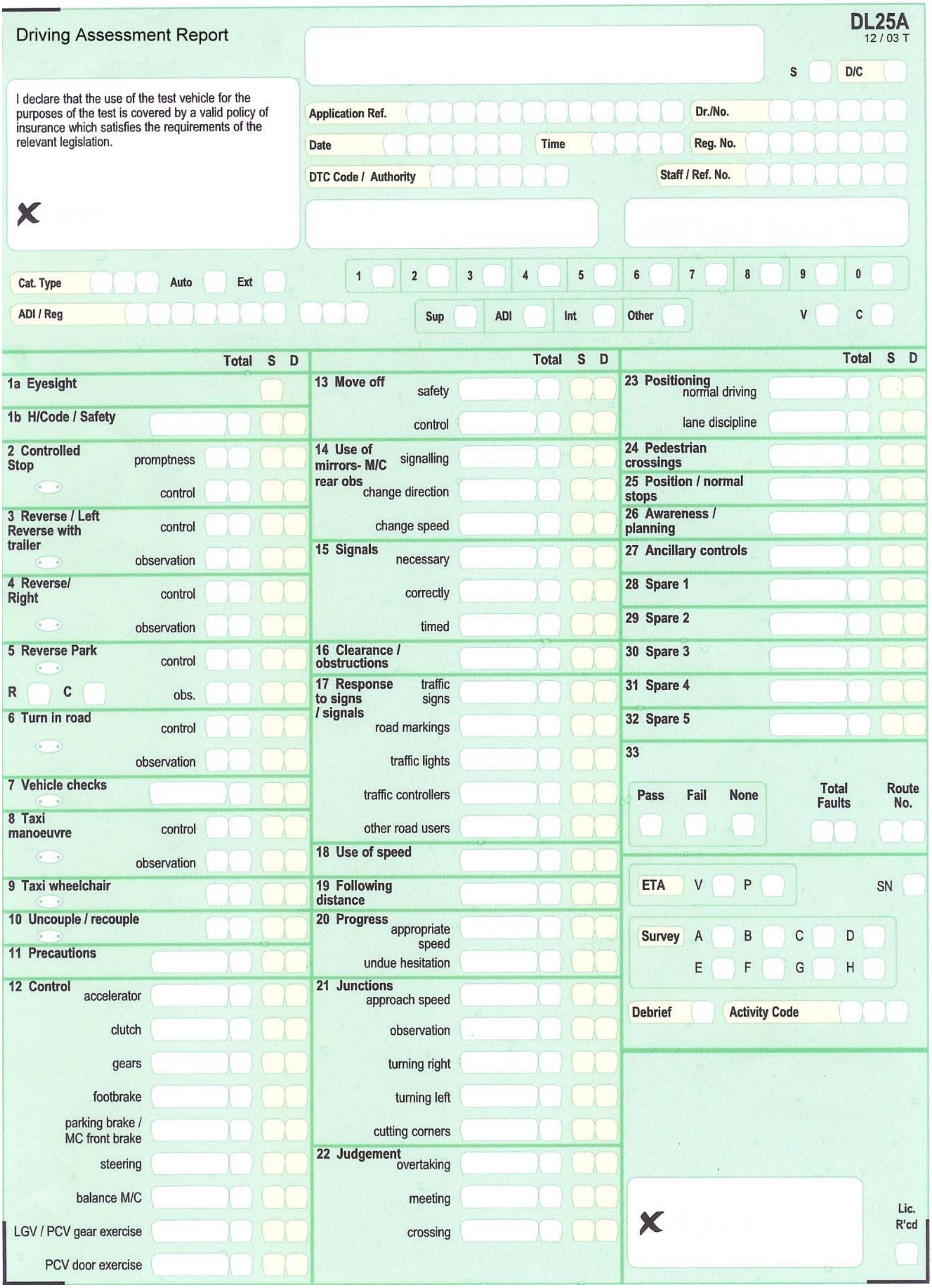

Driving Assessment Report

DL25A
12 / 03 T

S | D/C

I declare that the use of the test vehicle for the purposes of the test is covered by a valid policy of insurance which satisfies the requirements of the relevant legislation.

X

Application Ref. | Dr./No.
Date | Time | Reg. No.
DTC Code / Authority | Staff / Ref. No.

Cat. Type | Auto | Ext
1 | 2 | 3 | 4 | 5 | 6 | 7 | 8 | 9 | 0
ADI / Reg | Sup | ADI | Int | Other | V | C

	Total	S	D
1a Eyesight			
1b H/Code / Safety			
2 Controlled Stop – promptness			
control			
3 Reverse / Left Reverse with trailer – control			
observation			
4 Reverse/ Right – control			
observation			
5 Reverse Park – control			
R C obs.			
6 Turn in road – control			
observation			
7 Vehicle checks			
8 Taxi manoeuvre – control			
observation			
9 Taxi wheelchair			
10 Uncouple / recouple			
11 Precautions			
12 Control – accelerator			
clutch			
gears			
footbrake			
parking brake / MC front brake			
steering			
balance M/C			
LGV / PCV gear exercise			
PCV door exercise			

	Total	S	D
13 Move off – safety			
control			
14 Use of mirrors- M/C rear obs – signalling			
change direction			
change speed			
15 Signals – necessary			
correctly			
timed			
16 Clearance / obstructions			
17 Response to signs / signals – traffic signs			
road markings			
traffic lights			
traffic controllers			
other road users			
18 Use of speed			
19 Following distance			
20 Progress – appropriate speed			
undue hesitation			
21 Junctions – approach speed			
observation			
turning right			
turning left			
cutting corners			
22 Judgement – overtaking			
meeting			
crossing			

	Total	S	D
23 Positioning – normal driving			
lane discipline			
24 Pedestrian crossings			
25 Position / normal stops			
26 Awareness / planning			
27 Ancillary controls			
28 Spare 1			
29 Spare 2			
30 Spare 3			
31 Spare 4			
32 Spare 5			

33

Pass | Fail | None | Total Faults | Route No.

ETA V P | SN

Survey A B C D E F G H

Debrief | Activity Code

X

Lic. R'cd

3.2 Theory Test

DSA provided details of the theory test scores for drivers in the cohort study for tests taken after the introduction of hazard perception testing (November 2002).

Theory test scores were not available for all respondents. Where a respondent took more than one theory test, there is a record in the data for each occurrence (there may still be occurrences missing, including the one that was passed).

Theory test scores are not available for the majority of respondents in cohorts A to E, since the Hazard perception testing did not start until after the test dates of these cohorts. However, there are some TT scores for respondents in these cohorts, which will correspond to candidates who failed their driving test and needed to retake their theory test when the hazard perception element was included.

Where the theory test score data was available the following data are supplied:

- ID (used to match to questionnaire data)
- Cohort
- Test date
- Multiple choice score
- Hazard perception score

Following the introduction of hazard perception testing as part of the theory test, the pass mark was increased over the first year as shown in Table 4.

Table 4: Theory test pass marks

Date	Multiple choice pass mark (out of 35)	Hazard perception pass mark (out of 70)
14/11/2002	30	38
01/03/2003	30	40
01/07/2003	30	42
01/09/2003	30	44

Candidates are required to pass both parts of the theory test at the same time, and must take their practical driving test within two years of passing the theory test.

4 References

Wells P, Tong S, Sexton B, Grayson G and Jones E (2008). Cohort II: a study of learner and new drivers (Volume 1). Road Safety Research Report 81, Department for Transport, London. (To be published)

COPY

3443

LEARNING TO DRIVE QUESTIONNAIRE

Please complete this questionnaire by ticking the appropriate boxes and filling in the spaces as required. Several questions ask for dates or lengths of time etc, if you are unable to give an exact answer to any of these questions please give as good an estimate as you can. It will only take a few minutes to complete this questionnaire and any information you provide will be treated in the **strictest confidence** and will be used for statistical purposes only.

SECTION A: YOUR PRACTICAL DRIVING TEST

A1 Did you take your practical driving test in the first week of November 2001?

Yes ☐ ➡ *Go to A2*

No ☐ ➡ *Go to E7*

A2 When you took your practical driving test in the first week of November 2001 what type of licence did you hold?

(Tick ONE box only)

Provisional licence to drive a car ☐ ➡ *Go to A3*

Full licence for another class of vehicle ☐ ➡ *Go to E7*

A3 Had you previously held a FULL (GB or non- GB) car driving licence before taking your practical driving test in the first week of November 2001?

(Tick ONE box only)

NO ☐ ➡ *Go to A4*

YES – Full GB licence ☐ ➡ *Go to E7*

YES – Full non- GB licence ☐ ➡ *Go to E7*

3540
TRM

A

A4 How old were you when you got your first provisional car licence?

_______________ years _______________ months

A5 Before you started learning to drive a car did you regularly ride a motorcycle, moped, or scooter?

Yes ☐

No ☐

A6 Did you take your practical driving test in the first week of November 2001 in:
(Tick ONE box only)

A driving instructor's car? ☐ 1

A car owned by a friend/relation? ☐ 2

Your own car? ☐ 3

Another car? ☐ 4

A7 Was this practical driving test your:
(Tick ONE box only)

First? ☐ 1 ➨ *Go to A9*

Second? ☐ 2

Third? ☐ 3

Fourth? ☐ 4

More than fourth? ☐ 5

A8 When did you fail the practical driving test before the one in the first week of November 2001?

Day_______________ Month _______________ Year _______________

A9 Did you pass the practical driving test that you took in the first week of November 2001?

Yes ☐ ➨ *Go to B1*

No ☐

A10 Did you agree with the Examiner's decision to fail you?

Yes ☐

No ☐

A11 Why do you think you failed on this occasion? ***(Tick ALL that apply)***

You were just 'unlucky' on the day	☐	1
Difficult weather conditions on the day of the test	☐	2
You were not ready for the test	☐	3
Nerves affected your performance	☐	4
Another road user was to blame	☐	5
Your driving was not good enough on the day	☐	6
You could not cope with something unexpected that happened	☐	7
Other (please specify)	☐	8

____

A12 Since failing the practical driving test in the first week of November 2001, have you applied for another test?

Yes ☐

No ☐

A13 Before your next test do you think that you will... ***(Tick ONE box only)***

Have professional driving lessons MORE OFTEN?	☐	1
Have professional driving lessons AS OFTEN AS BEFORE?	☐	2
Have professional driving lessons LESS OFTEN?	☐	3
START having professional driving lessons?	☐	4
NOT HAVE ANY professional driving lessons?	☐	5

A14 Before your next test do you think that you will... ***(Tick ONE box only)***

Have practice driving sessions with friends or relations MORE OFTEN?	☐	1
Have practice driving sessions with friends or relations AS OFTEN AS BEFORE?	☐	2
Have practice driving sessions with friends or relations LESS OFTEN?	☐	3
START having practice driving sessions with friends or relations?	☐	4
NOT HAVE ANY practice driving sessions with friends or relations?	☐	5

SECTION B: LEARNING TO DRIVE

B1 When did you begin to learn to drive a car?

Month ____________________ Year __________

B2 Since you started learning to drive, was there any period of longer than 6 months during which you did no driving at all?

Yes ☐

No ☐ ➨ ***Go to B4***

B3 What was the total of all those periods during which you did no driving at all since you started learning to drive?

__________ years __________ months

B4 How many miles IN TOTAL had you driven a car before you took your practical driving test in the first week of November 2001?
(If you are not sure, please give your best estimate to the nearest 100 miles)

__________ miles

B5 Please estimate how your total driving time was split. ***(Tick ONE box only)***

All in a DRIVING INSTRUCTOR'S car	Almost all in a DRIVING INSTRUCTOR'S car	More than half in a DRIVING INSTRUCTOR'S car	About half and half	More than half in OTHER car(s)	Almost all in OTHER car(s)	All in OTHER car(s)
(1)	(2)	(3)	(4)	(5)	(6)	(7)
☐	☐	☐	☐	☐	☐	☐

B6 How many hours of lessons from a professional driving instructor have you had:

(a) **IN TOTAL since you first started learning to drive?**

__________ hours ➨ ***If none, please go to B11***

(b) **Between the practical driving test that you took in the first week of November 2001 and your previous test?**

__________ hours No previous practical driving test ☐

B7 How many different professional driving instructors have you ever had lessons from?

__________ (Please write number of instructors)

B8 (a) **Was your most recent professional driving instructor a DSA Approved Driving Instructor (ADI)?**

- Yes ☐
- No ☐
- Don't know ☐

(b) **What grade of instructor is he or she?** *(Tick ONE box only)*

- Grade 6 ADI ☐ 1
- Grade 5 ADI ☐ 2
- Grade 4 ADI ☐ 3
- Trainee ADI ☐ 4
- Don't know ☐ 5

(c) **What was your main reason for choosing this instructor/driving school?**

(Tick ONE box only)

- Good reputation ☐ 1
- Personal recommendation ☐ 2
- You already knew the person ☐ 3
- Cost of lessons ☐ 4
- Local advertising ☐ 5
- Large company/organisation ☐ 6
- You saw them in the phone book ☐ 7
- Highly qualified instructors ☐ 8
- Other (*please specify*) ☐ 9

____

B9 **Thinking about ALL the professional driving lessons that you took from when you started driving up to the time of your driving test in the first week of November 2001, how much have you driven...**

(Tick ONE box per line)

		Never	Less than 2 hours	2-4 hours	More than 4 hours
(a)	In a busy town centre	☐	☐	☐	☐
(b)	On country roads	☐	☐	☐	☐
(c)	On fast dual carriageways	☐	☐	☐	☐
(d)	In the dark	☐	☐	☐	☐
(e)	In the rain	☐	☐	☐	☐
(f)	On snow or ice	☐	☐	☐	☐

B10 When you were preparing for the practical driving test you took in the first week of November 2001, how did you spread out your professional lessons?

(Tick ONE box only)

Regularly up to the practical driving test just taken	☐	1
Most at the beginning with a few near the test	☐	2
Most near the test with a few at the beginning	☐	3
Some at the beginning and some near the test with a few in between	☐	4
Few at the beginning and few near the test with most in between	☐	5
Other (*please specify*)	☐	6

____

B11 How many hours did you spend practising your driving with friends or relations?

(a) IN TOTAL since you first started learning to drive?

__________ hours ➡ ***If none, please go to to B14***

(b) Between the practical driving test that you took in the first week of November 2001 and your previous car driving test?

__________ hours No previous practical driving test ☐

B12 Thinking about ALL the practice driving with friends and relatives that you did from when you started driving up to the time of your driving test in the first week of November 2001, how much have you driven...

(Tick ONE box per line)

	Never	Less than 2 hours	2-4 hours	More than 4 hours
**(a)** In a busy town centre	☐	☐	☐	☐
**(b)** On country roads	☐	☐	☐	☐
**(c)** On fast dual carriageways	☐	☐	☐	☐
**(d)** In the dark	☐	☐	☐	☐
**(e)** In the rain	☐	☐	☐	☐
**(f)** On snow or ice	☐	☐	☐	☐

B13 When you were preparing for the practical driving test you took in the first week of November 2001, how did you spread out your sessions of driving practice with friends and relations?

(*Tick ONE box only*)

Regularly up to the practical driving test just taken	☐	1
Most at the beginning with a few near the test	☐	2
Most near the test with a few at the beginning	☐	3
Some at the beginning and some near the test with a few in between	☐	4
Few at the beginning and few near the test with most in between	☐	5
Other (*please specify*)	☐	6

____

B14 When you were preparing for the practical driving test you took in the first week of November 2001:

		Yes	No
**(a)**	Was it hard to find a friend or relation to take you out for driving practice?	☐	☐
**(b)**	Were you able to have as much driving practice with friends or relations as you wanted?	☐	☐
**(c)**	Was it difficult to find time to fit in driving practice with friends or relations?	☐	☐
**(d)**	Did the cost of professional lessons affect the number you took?	☐	☐
**(e)**	Do you feel that you had professional lessons often enough?	☐	☐
**(f)**	Was it difficult to find time to fit in professional lessons?	☐	☐
**(g)**	When you applied for the test did you think you had a good chance of passing it?	☐	☐
**(h)**	When you took the test did you think you had a good chance of passing it?	☐	☐
**(i)**	Were you keen to take your test as soon as possible?	☐	☐
**(j)**	Did you find learning to drive easy?	☐	☐

SECTION C: YOUR THEORY TEST

C1 When did you pass your theory test for car drivers?

________________ month ________ year

C2 Was this the first time you had taken the theory test for car drivers?

Yes ☐ ➡ ***Go to C4***

No ☐

C3 (a) In total, how many times have you taken the theory test for car drivers?

________ times

(b) When did you take your FIRST theory test for car drivers?

________________ month ________ year

PLEASE ANSWER QUESTIONS C4 TO C7 ABOUT THE <u>FIRST</u> THEORY TEST THAT YOU TOOK

C4 Had you driven at all before taking the theory test?

Yes ☐

No ☐ ➡ ***Go to C6***

C5 How many hours driving experience had you had in total when you took your first theory test?
Please include both professional lessons and driving practice with friends or relations.

________ **hours**

C6 In your opinion, how ready to apply for your practical driving test were you when you took your theory test?

(Tick ONE box only)

I was ready to apply for my practical driving test ☐

I was nearly ready to apply for my practical driving test ☐

I was not at all ready to apply for my practical driving test ☐

C7 Which of the following did your professional driving instructor do...

	(i) when preparing for your theory test	(ii) between your theory test and practical test
	(Tick ALL that apply)	
Taught or tested you on knowledge of road signs and markings	☐ 1	☐ 1
Taught or tested you on other driving- related knowledge	☐ 2	☐ 2
Taught or tested you on hazard awareness skills	☐ 3	☐ 3
Recommended particular driving-related books or materials	☐ 4	☐ 4
Went through example theory test questions	☐ 5	☐ 5
Did not have any lessons with a professional driving instructor	☐ 6	☐ 6
Other (*please specify*) ______________________	☐ 7	☐ 7
Nothing	☐ 8	☐ 8

C8 Which of the following did you use when preparing for your theory and practical driving tests?

	(i) Theory test	(ii) Practical test
	(Tick ALL that apply)	
DSA 'Official theory Test for Car Drivers'	☐ 1	☐ 1
Other example questions	☐ 2	☐ 2
DSA 'The Driving Manual'	☐ 3	☐ 3
Highway code	☐ 4	☐ 4
Any other driving related text book	☐ 5	☐ 5
Driving related CD-Rom	☐ 6	☐ 6
Driving related videos	☐ 7	☐ 7
Internet website	☐ 8	☐ 8
Other (*please specify*) ______________________	☐ 9	☐ 9
I did not use any material to prepare for the test	☐ 10	☐ 10

SECTION D: ATTITUDES TOWARDS DRIVING

D1 The following questions ask about your confidence in your own driving ability.

(Please tick ONE box per line)

		Very confident	Fairly confident	Not very confident	Not at all confident
(a)	How confident were you in your driving ability when you were learning to drive?	☐	☐	☐	☐
(b)	In the week before you took your practical driving test, how confident were you that you would pass?	☐	☐	☐	☐
(c)	In the week before you took your practical driving test, how confident were you that you were a good enough driver to drive on the road unsupervised?	☐	☐	☐	☐
(d)	How confident are you in your driving ability now?	☐	☐	☐	☐

D2 How much do you think you need to improve your ability on each of the following driving skills?

(Tick ONE box per line)

		No improvement needed	Some improvement needed	A lot of improvement needed
(a)	Use of car controls	☐	☐	☐
(b)	Pulling out of junctions	☐	☐	☐
(c)	Reversing	☐	☐	☐
(d)	Parking	☐	☐	☐
(e)	Judging the speed of other traffic	☐	☐	☐
(f)	Judging what other drivers are going to do	☐	☐	☐
(g)	Spotting hazards	☐	☐	☐
(h)	Driving in heavy traffic	☐	☐	☐
(i)	Driving in the dark	☐	☐	☐
(j)	Overtaking	☐	☐	☐
(k)	Using roundabouts	☐	☐	☐
(l)	Joining with moving traffic on a motorway or fast dual carriageway	☐	☐	☐
(m)	Changing lanes on a motorway or fast dual carriageway	☐	☐	☐
(n)	Driving on high speed roads	☐	☐	☐
(o)	Driving on country roads	☐	☐	☐

D3 How much do you agree or disagree with each of the following statements?

(Tick ONE box on each line)

		Strongly agree (1)	Agree (2)	Neither agree nor disagree (3)	Disagree (4)	Strongly disagree (5)
(a)	Decreasing the motorway speed limit is a good idea					
(b)	Even at night-time on quiet roads it is important to keep within the speed limit					
(c)	Drivers who cause accidents by reckless driving should be banned from driving for life					
(d)	People should drive slower than the speed limit when it is raining					
(e)	Cars should never overtake on the inside lane even if a slow driver is blocking the outside lane					
(f)	In towns where there are a lot of pedestrians the speed limit should be 20mph					
(g)	Penalties for speeding should be more severe					
(h)	Increasing the motorway speed limit is a good idea					

D4 We would like to know what kind of driver you think you are by putting a tick somewhere on each of the lines below.

At either end of each line there is a word that describes a way of driving, and these words are opposites. Put your tick nearer to the word that best describes your driving. The closer your tick is to the word, the more you agree with this description of the way you drive.

	(1)	(2)	(3)	(4)	(5)	(6)	(7)	
Attentive								Inattentive
Careful								Careless
Decisive								Indecisive
Experienced								Inexperienced
Irritable								Placid
Nervous								Confident
Patient								Impatient
Responsible								Irresponsible
Safe								Risky
Selfish								Considerate
Slow								Fast
Tolerant								Intolerant

SECTION E: CONTACT DETAILS

For the success of this important research, we would like to get in touch with you again to see how your driving is going.

Please fill in as many of the contact details below as possible.

All information will be treated in the STRICTEST CONFIDENCE and will not be used for any purpose other than to get in touch with you in relation to this research.

E1 Home phone number: ______________________

E2 Work phone number: ______________________
(*if applicable*)

E3 Mobile phone number: ______________________

E4 Email address: ______________________

If the address that this questionnaire was sent to is incorrect, or if there is a better address to help us contact you in the future, please provide the new address in the space below.

E5 New address: ______________________

____

____

Postcode: ______________________

E6 If you have any further comments to make about this survey and the issues raised, please write in the space provided:

E7 Please now return this questionnaire in the pre-paid envelope to:
NFER, The Mere, Upton Park, Slough, Berkshire, SL1 2DQ

Please return this questionnaire even if you have only answered the first few questions, as this information is equally important to us and will allow us to remove your name from our mailing list for future questionnaires.

Thank you very much for your help.

LEARNING TO DRIVE QUESTIONNAIRE

Please complete this questionnaire by ticking the appropriate boxes and filling in the spaces as required. Several questions ask for dates or lengths of time etc, if you are unable to give an exact answer to any of these questions please give as good an estimate as you can. It will only take a few minutes to complete this questionnaire and any information you provide will be treated in the **strictest confidence** and will be used for statistical purposes only.

SECTION A: YOUR PRACTICAL DRIVING TEST

A1 Did you take your practical driving test in the week beginning Monday 4th February 2002?

Yes ☐ ➡ *Go to A2*

No ☐ ➡ *Go to E7*

A2 When you took your practical driving test in the week beginning Monday 4th February 2002 what type of licence did you hold?

(Tick ONE box only)

Provisional licence to drive a car ☐ ➡ *Go to A3*

Full licence for another class of vehicle ☐ ➡ *Go to E7*

A3 Had you previously held a FULL (GB or non- GB) car driving licence before taking your practical driving test in the week beginning Monday 4th February 2002?

(Tick ONE box only)

NO ☐ ➡ *Go to A4*

YES – Full GB licence ☐ ➡ *Go to E7*

YES – Full non- GB licence ☐ ➡ *Go to E7*

A4 How old were you when you got your first provisional car licence?

__________ years __________ months

A5 Before you started learning to drive a car did you regularly ride a motorcycle, moped, or scooter?

Yes ☐

No ☐

A6 Did you take your practical driving test in the week beginning Monday 4th February 2002:
(Tick ONE box only)

A driving instructor's car? ☐ 1

A car owned by a friend/relation? ☐ 2

Your own car? ☐ 3

Another car? ☐ 4

A7 Was this practical driving test your:
(Tick ONE box only)

First? ☐ 1 ➡ *Go to A9*

Second? ☐ 2

Third? ☐ 3

Fourth? ☐ 4

More than fourth? ☐ 5

A8 When did you fail the practical driving test before the one in the week beginning Monday 4th February 2002?

Day__________ Month ____________________ Year __________

A9 Did you pass the practical driving test that you took in the week beginning Monday 4th February 2002?

Yes ☐ ➡ *Go to B1*

No ☐

A10 Did you agree with the Examiner's decision to fail you?

Yes ☐

No ☐

A11 Why do you think you failed on this occasion? *(Tick ALL that apply)*

You were just 'unlucky' on the day	☐	1
Difficult weather conditions on the day of the test	☐	2
You were not ready for the test	☐	3
Nerves affected your performance	☐	4
Another road user was to blame	☐	5
Your driving was not good enough on the day	☐	6
You could not cope with something unexpected that happened	☐	7
Other (please specify)	☐	8

____

A12 Since failing the practical driving test in the week beginning Monday 4th February 2002, have you applied for another test?

Yes ☐

No ☐

A13 Before your next test do you think that you will... *(Tick ONE box only)*

Have professional driving lessons MORE OFTEN?	☐	1
Have professional driving lessons AS OFTEN AS BEFORE?	☐	2
Have professional driving lessons LESS OFTEN?	☐	3
START having professional driving lessons?	☐	4
NOT HAVE ANY professional driving lessons?	☐	5

A14 Before your next test do you think that you will... *(Tick ONE box only)*

Have practice driving sessions with friends or relations MORE OFTEN?	☐	1
Have practice driving sessions with friends or relations AS OFTEN AS BEFORE?	☐	2
Have practice driving sessions with friends or relations LESS OFTEN?	☐	3
START having practice driving sessions with friends or relations?	☐	4
NOT HAVE ANY practice driving sessions with friends or relations?	☐	5

SECTION B: LEARNING TO DRIVE

B1 When did you begin to learn to drive a car?

Month ______________________ Year ____________

B2 Since you started learning to drive, was there any period of longer than 6 months during which you did no driving at all?

Yes ☐

No ☐ ➡ ***Go to B4***

B3 What was the total of all those periods during which you did no driving at all since you started learning to drive?

____________ years ____________ months

B4 How many miles IN TOTAL had you driven a car before you took your practical driving test in the week beginning Monday 4th February 2002?
(If you are not sure, please give your best estimate to the nearest 100 miles)

____________ miles

B5 Please estimate how your total driving time was split. ***(Tick ONE box only)***

All in a DRIVING INSTRUCTOR'S car	Almost all in a DRIVING INSTRUCTOR'S car	More than half in a DRIVING INSTRUCTOR'S car	About half and half	More than half in OTHER car(s)	Almost all in OTHER car(s)	All in OTHER car(s)
(1)	(2)	(3)	(4)	(5)	(6)	(7)
☐	☐	☐	☐	☐	☐	☐

B6 How many hours of lessons from a professional driving instructor have you had:

(a) IN TOTAL since you first started learning to drive?

____________ hours ➡ ***If none, please go to B11***

(b) Between the practical driving test that you took in the week beginning Monday 4th February 2002 and your previous test?

____________ hours No previous practical driving test ☐

B7 How many different professional driving instructors have you ever had lessons from?

____________ (Please write number of instructors)

B8 (a) Was your most recent professional driving instructor a DSA Approved Driving Instructor (ADI)?

- Yes ☐
- No ☐
- Don't know ☐

(b) What grade of instructor is he or she? *(Tick ONE box only)*

- Grade 6 ADI ☐ 1
- Grade 5 ADI ☐ 2
- Grade 4 ADI ☐ 3
- Trainee ADI ☐ 4
- Don't know ☐ 5

(c) What was your main reason for choosing this instructor/driving school?
(Tick ONE box only)

- Good reputation ☐ 1
- Personal recommendation ☐ 2
- You already knew the person ☐ 3
- Cost of lessons ☐ 4
- Local advertising ☐ 5
- Large company/organisation ☐ 6
- You saw them in the phone book ☐ 7
- Highly qualified instructors ☐ 8
- Other (*please specify*) ☐ 9

____

B9 Thinking about ALL the professional driving lessons that you took from when you started driving up to the time of your driving test in the week beginning Monday 4th February 2002, how much have you driven...

(Tick ONE box per line)

		Never	Less than 2 hours	2-4 hours	More than 4 hours
(a)	In a busy town centre	☐	☐	☐	☐
(b)	On country roads	☐	☐	☐	☐
(c)	On fast dual carriageways	☐	☐	☐	☐
(d)	In the dark	☐	☐	☐	☐
(e)	In the rain	☐	☐	☐	☐
(f)	On snow or ice	☐	☐	☐	☐

B10 When you were preparing for the practical driving test you took in the week beginning Monday 4th February 2002, how did you spread out your professional lessons?

(Tick ONE box only)

Regularly up to the practical driving test just taken	☐	1
Most at the beginning with a few near the test	☐	2
Most near the test with a few at the beginning	☐	3
Some at the beginning and some near the test with a few in between	☐	4
Few at the beginning and few near the test with most in between	☐	5
Other (*please specify*)	☐	6

____

B11 How many hours did you spend practising your driving with friends or relations?

(a) IN TOTAL since you first started learning to drive?

__________ hours ➡ ***If none, please go to to B14***

(b) Between the practical driving test that you took in the week beginning Monday 4th February 2002 and your previous car driving test?

__________ hours No previous practical driving test ☐

B12 Thinking about ALL the practice driving with friends and relatives that you did from when you started driving up to the time of your driving test in the week beginning Monday 4th February 2002, how much have you driven...

(Tick ONE box per line)

	Never	Less than 2 hours	2-4 hours	More than 4 hours
(a) In a busy town centre	☐	☐	☐	☐
(b) On country roads	☐	☐	☐	☐
(c) On fast dual carriageways	☐	☐	☐	☐
(d) In the dark	☐	☐	☐	☐
(e) In the rain	☐	☐	☐	☐
(f) On snow or ice	☐	☐	☐	☐

B13 When you were preparing for the practical driving test you took in the week beginning Monday 4th February 2002, how did you spread out your sessions of driving practice with friends and relations?

(Tick ONE box only)

Regularly up to the practical driving test just taken	☐	1
Most at the beginning with a few near the test	☐	2
Most near the test with a few at the beginning	☐	3
Some at the beginning and some near the test with a few in between	☐	4
Few at the beginning and few near the test with most in between	☐	5
Other (*please specify*)	☐	6

____

B14 When you were preparing for the practical driving test you took in the week beginning Monday 4th February 2002:

		**Yes**	**No**
**(a)**	Was it hard to find a friend or relation to take you out for driving practice?	☐	☐
**(b)**	Were you able to have as much driving practice with friends or relations as you wanted?	☐	☐
**(c)**	Was it difficult to find time to fit in driving practice with friends or relations?	☐	☐
**(d)**	Did the cost of professional lessons affect the number you took?	☐	☐
**(e)**	Do you feel that you had professional lessons often enough?	☐	☐
**(f)**	Was it difficult to find time to fit in professional lessons?	☐	☐
**(g)**	When you applied for the test did you think you had a good chance of passing it?	☐	☐
**(h)**	When you took the test did you think you had a good chance of passing it?	☐	☐
**(i)**	Were you keen to take your test as soon as possible?	☐	☐
**(j)**	Did you find learning to drive easy?	☐	☐

SECTION C: YOUR THEORY TEST

C1 When did you pass your theory test for car drivers?

________________ month __________ year

C2 Was this the first time you had taken the theory test for car drivers?

Yes ☐ ➡ *Go to C4*

No ☐

C3 (a) In total, how many times have you taken the theory test for car drivers?

__________ times

(b) When did you take your FIRST theory test for car drivers?

________________ month __________ year

PLEASE ANSWER QUESTIONS C4 TO C7 ABOUT THE FIRST THEORY TEST THAT YOU TOOK

C4 Had you driven at all before taking the theory test?

Yes ☐

No ☐ ➡ *Go to C6*

C5 How many hours driving experience had you had in total when you took your first theory test?
Please include both professional lessons and driving practice with friends or relations.

__________ **hours**

C6 In your opinion, how ready to apply for your practical driving test were you when you took your theory test?

(Tick ONE box only)

I was ready to apply for my practical driving test ☐

I was nearly ready to apply for my practical driving test ☐

I was not at all ready to apply for my practical driving test ☐

C7 Which of the following did your professional driving instructor do...

(Tick ALL that apply)

	(i) when preparing for your theory test	(ii) between your theory test and practical test
Taught or tested you on knowledge of road signs and markings	☐ 1	☐ 1
Taught or tested you on other driving- related knowledge	☐ 2	☐ 2
Taught or tested you on hazard awareness skills	☐ 3	☐ 3
Recommended particular driving-related books or materials	☐ 4	☐ 4
Went through example theory test questions	☐ 5	☐ 5
Did not have any lessons with a professional driving instructor	☐ 6	☐ 6
Other (*please specify*) ______________________	☐ 7	☐ 7
Nothing	☐ 8	☐ 8

C8 Which of the following did you use when preparing for your theory and practical driving tests?

(Tick ALL that apply)

	(i) Theory test	(ii) Practical test
DSA 'Official theory Test for Car Drivers'	☐ 1	☐ 1
Other example questions	☐ 2	☐ 2
DSA 'The Driving Manual'	☐ 3	☐ 3
Highway code	☐ 4	☐ 4
Any other driving related text book	☐ 5	☐ 5
Driving related CD-Rom	☐ 6	☐ 6
Driving related videos	☐ 7	☐ 7
Internet website	☐ 8	☐ 8
Other (*please specify*) ______________________	☐ 9	☐ 9
I did not use any material to prepare for the test	☐ 10	☐ 10

SECTION D: ATTITUDES TOWARDS DRIVING

D1 The following questions ask about your confidence in your own driving ability.

(Please tick ONE box per line)

		Very confident	Fairly confident	Not very confident	Not at all confident
(a)	How confident were you in your driving ability when you were learning to drive?	☐	☐	☐	☐
(b)	In the week before you took your practical driving test, how confident were you that you would pass?	☐	☐	☐	☐
(c)	In the week before you took your practical driving test, how confident were you that you were a good enough driver to drive on the road unsupervised?	☐	☐	☐	☐
(d)	How confident are you in your driving ability now?	☐	☐	☐	☐

D2 How much do you think you need to improve your ability on each of the following driving skills?

(Tick ONE box per line)

		No improvement needed	Some improvement needed	A lot of improvement needed
(a)	Use of car controls	☐	☐	☐
(b)	Pulling out of junctions	☐	☐	☐
(c)	Reversing	☐	☐	☐
(d)	Parking	☐	☐	☐
(e)	Judging the speed of other traffic	☐	☐	☐
(f)	Judging what other drivers are going to do	☐	☐	☐
(g)	Spotting hazards	☐	☐	☐
(h)	Driving in heavy traffic	☐	☐	☐
(i)	Driving in the dark	☐	☐	☐
(j)	Overtaking	☐	☐	☐
(k)	Using roundabouts	☐	☐	☐
(l)	Joining with moving traffic on a motorway or fast dual carriageway	☐	☐	☐
(m)	Changing lanes on a motorway or fast dual carriageway	☐	☐	☐
(n)	Driving on high speed roads	☐	☐	☐
(o)	Driving on country roads	☐	☐	☐

D3 How much do you agree or disagree with each of the following statements?

(Tick ONE box on each line)

		Strongly agree (1)	Agree (2)	Neither agree nor disagree (3)	Disagree (4)	Strongly disagree (5)
(a)	Decreasing the motorway speed limit is a good idea	☐	☐	☐	☐	☐
(b)	Even at night-time on quiet roads it is important to keep within the speed limit	☐	☐	☐	☐	☐
(c)	Drivers who cause accidents by reckless driving should be banned from driving for life	☐	☐	☐	☐	☐
(d)	People should drive slower than the speed limit when it is raining	☐	☐	☐	☐	☐
(e)	Cars should never overtake on the inside lane even if a slow driver is blocking the outside lane	☐	☐	☐	☐	☐
(f)	In towns where there are a lot of pedestrians the speed limit should be 20mph	☐	☐	☐	☐	☐
(g)	Penalties for speeding should be more severe	☐	☐	☐	☐	☐
(h)	Increasing the motorway speed limit is a good idea	☐	☐	☐	☐	☐

D4 We would like to know what kind of driver you think you are by putting a tick somewhere on each of the lines below.

At either end of each line there is a word that describes a way of driving, and these words are opposites. Put your tick nearer to the word that best describes your driving. The closer your tick is to the word, the more you agree with this description of the way you drive.

	(1)	(2)	(3)	(4)	(5)	(6)	(7)	
Attentive								Inattentive
Careful								Careless
Decisive								Indecisive
Experienced								Inexperienced
Irritable								Placid
Nervous								Confident
Patient								Impatient
Responsible								Irresponsible
Safe								Risky
Selfish								Considerate
Slow								Fast
Tolerant								Intolerant

SECTION E: CONTACT DETAILS

For the success of this important research, we would like to get in touch with you again to see how your driving is going.

Please fill in as many of the contact details below as possible.

All information will be treated in the STRICTEST CONFIDENCE and will not be used for any purpose other than to get in touch with you in relation to this research.

E1 Home phone number: ______________________

E2 Work phone number: ______________________
(*if applicable*)

E3 Mobile phone number: ______________________

E4 Email address: ______________________

If the address that this questionnaire was sent to is incorrect, or if there is a better address to help us contact you in the future, please provide the new address in the space below.

E5 New address: ______________________

____

____

Postcode: ______________________

E6 If you have any further comments to make about this survey and the issues raised, please write in the space provided:

E7 Please now return this questionnaire in the pre-paid envelope to:
NFER, The Mere, Upton Park, Slough, Berkshire, SL1 2DQ

Please return this questionnaire even if you have only answered the first few questions, as this information is equally important to us and will allow us to remove your name from our mailing list for future questionnaires.

Thank you very much for your help.

DTLR
TRANSPORT
LOCAL GOVERNMENT
REGIONS

TRL

LEARNING TO DRIVE QUESTIONNAIRE

Please complete this questionnaire by ticking the appropriate boxes and filling in the spaces as required. Several questions ask for dates or lengths of time etc, if you are unable to give an exact answer to any of these questions please give as good an estimate as you can. It will only take a few minutes to complete this questionnaire and any information you provide will be treated in the **strictest confidence** and will be used for statistical purposes only.

SECTION A: YOUR PRACTICAL DRIVING TEST

A1 Did you take your practical driving test in the week beginning Monday 29th April 2002?

Yes ☐ ➡ *Go to A2*

No ☐ ➡ *Go to E7*

A2 When you took your practical driving test in the week beginning Monday 29th April 2002 what type of licence did you hold?

(Tick ONE box only)

Provisional licence to drive a car ☐ ➡ *Go to A3*

Full licence for another class of vehicle ☐ ➡ *Go to E7*

A3 Had you previously held a FULL (GB or non- GB) car driving licence before taking your practical driving test in the week beginning Monday 29th April 2002?

(Tick ONE box only)

NO ☐ ➡ *Go to A4*

YES – Full GB licence ☐ ➡ *Go to E7*

YES – Full non- GB licence ☐ ➡ *Go to E7*

A4 **How old were you when you got your first provisional car licence?**

____________ years ____________ months

A5 **Before you started learning to drive a car did you regularly ride a motorcycle, moped, or scooter?**

Yes ☐

No ☐

A6 **Did you take your practical driving test in the week beginning Monday 29th April 2002 in:**
(Tick ONE box only)

A driving instructor's car? ☐ 1

A car owned by a friend/relation? ☐ 2

Your own car? ☐ 3

Another car? ☐ 4

A7 **Was this practical driving test your:**
(Tick ONE box only)

First? ☐ 1 ➧ ***Go to A9***

Second? ☐ 2

Third? ☐ 3

Fourth? ☐ 4

More than fourth? ☐ 5

A8 **When did you fail the practical driving test before the one in the week beginning Monday 29th April 2002?**

Day__________ Month ____________________ Year __________

A9 **Did you pass the practical driving test that you took in the week beginning Monday 29th April 2002?**

Yes ☐ ➧ ***Go to B1***

No ☐

A10 **Did you agree with the Examiner's decision to fail you?**

Yes ☐

No ☐

A11 Why do you think you failed on this occasion? ***(Tick ALL that apply)***

You were just 'unlucky' on the day	☐	1
Difficult weather conditions on the day of the test	☐	2
You were not ready for the test	☐	3
Nerves affected your performance	☐	4
Another road user was to blame	☐	5
Your driving was not good enough on the day	☐	6
You could not cope with something unexpected that happened	☐	7
Other (please specify)	☐	8

____

A12 Since failing the practical driving test in the week beginning Monday 29th April 2002, have you applied for another test?

Yes ☐

No ☐

A13 Before your next test do you think that you will... ***(Tick ONE box only)***

Have professional driving lessons MORE OFTEN?	☐	1
Have professional driving lessons AS OFTEN AS BEFORE?	☐	2
Have professional driving lessons LESS OFTEN?	☐	3
START having professional driving lessons?	☐	4
NOT HAVE ANY professional driving lessons?	☐	5

A14 Before your next test do you think that you will... ***(Tick ONE box only)***

Have practice driving sessions with friends or relations MORE OFTEN?	☐	1
Have practice driving sessions with friends or relations AS OFTEN AS BEFORE?	☐	2
Have practice driving sessions with friends or relations LESS OFTEN?	☐	3
START having practice driving sessions with friends or relations?	☐	4
NOT HAVE ANY practice driving sessions with friends or relations?	☐	5

SECTION B: LEARNING TO DRIVE

B1 When did you begin to learn to drive a car?

Month ____________________ Year __________

B2 Since you started learning to drive, was there any period of longer than 6 months during which you did no driving at all?

Yes ☐

No ☐ ➡ *Go to B4*

B3 What was the total of all those periods during which you did no driving at all since you started learning to drive?

__________ years __________ months

B4 How many miles IN TOTAL had you driven a car before you took your practical driving test in the week beginning Monday 29th April 2002?
(If you are not sure, please give your best estimate to the nearest 100 miles)

__________ miles

B5 Please estimate how your total driving time was split. ***(Tick ONE box only)***

All in a DRIVING INSTRUCTOR'S car	Almost all in a DRIVING INSTRUCTOR'S car	More than half in a DRIVING INSTRUCTOR'S car	About half and half	More than half in OTHER car(s)	Almost all in OTHER car(s)	All in OTHER car(s)
(1)	(2)	(3)	(4)	(5)	(6)	(7)
☐	☐	☐	☐	☐	☐	☐

B6 How many hours of lessons from a professional driving instructor have you had:

(a) IN TOTAL since you first started learning to drive?

V00573 __________ hours **None** V00574 ☐ ➡ ***If none, please go to B11***

(b) Between the practical driving test that you took in the week beginning Monday 29th April 2002 and your previous test?

V0245 __________ hours No previous practical driving test V00117 ☐

B7 How many different professional driving instructors have you ever had lessons from?

__________ (Please write number of instructors)

B8 (a) Was your most recent professional driving instructor a DSA Approved Driving Instructor (ADI)?

Yes ☐

No ☐

Don't know ☐

(b) What grade of instructor is he or she? ***(Tick ONE box only)***

Grade 6 ADI ☐ 1

Grade 5 ADI ☐ 2

Grade 4 ADI ☐ 3

Trainee ADI ☐ 4

Don't know ☐ 5

(c) What was your main reason for choosing this instructor/driving school?

(Tick ONE box only)

Good reputation ☐ 1

Personal recommendation ☐ 2

You already knew the person ☐ 3

Cost of lessons ☐ 4

Local advertising ☐ 5

Large company/organisation ☐ 6

You saw them in the phone book ☐ 7

Highly qualified instructors ☐ 8

Other (*please specify*) ☐ 9

____

B9 Thinking about ALL the professional driving lessons that you took from when you started driving up to the time of your driving test in the week beginning Monday 29th April 2002, how much have you driven...

(Tick ONE box per line)

	Never	Less than 2 hours	2-4 hours	More than 4 hours
**(a)** In a busy town centre	☐	☐	☐	☐
**(b)** On country roads	☐	☐	☐	☐
**(c)** On fast dual carriageways	☐	☐	☐	☐
**(d)** In the dark	☐	☐	☐	☐
**(e)** In the rain	☐	☐	☐	☐
**(f)** On snow or ice	☐	☐	☐	☐

B10 When you were preparing for the practical driving test you took in the week beginning Monday 29th April 2002, how did you spread out your professional lessons?

(Tick ONE box only)

Regularly up to the practical driving test just taken	☐	1
Most at the beginning with a few near the test	☐	2
Most near the test with a few at the beginning	☐	3
Some at the beginning and some near the test with a few in between	☐	4
Few at the beginning and few near the test with most in between	☐	5
Other (*please specify*)	☐	6

____

B11 How many hours did you spend practising your driving with friends or relations?

V00455

(a) None ☐ ➡ ***If none, please go to B14***

(b) IN TOTAL since you first started learning to drive?

V00572

______________ hours

(c) Between the practical driving test that you took in the week beginning Monday 29th April 2002 and your previous car driving test?

V00348 V00116

______________ hours No previous practical driving test ☐

B12 Thinking about ALL the practice driving with friends and relatives that you did from when you started driving up to the time of your driving test in the week beginning Monday 29th April 2002, how much have you driven...

(Tick ONE box per line)

		Never	Less than 2 hours	2-4 hours	More than 4 hours
**(a)**	In a busy town centre	☐	☐	☐	☐
**(b)**	On country roads	☐	☐	☐	☐
**(c)**	On fast dual carriageways	☐	☐	☐	☐
**(d)**	In the dark	☐	☐	☐	☐
**(e)**	In the rain	☐	☐	☐	☐
**(f)**	On snow or ice	☐	☐	☐	☐

B13 When you were preparing for the practical driving test you took in the week beginning Monday 29th April 2002, how did you spread out your sessions of driving practice with friends and relations?

(*Tick ONE box only*)

Regularly up to the practical driving test just taken	☐	1
Most at the beginning with a few near the test	☐	2
Most near the test with a few at the beginning	☐	3
Some at the beginning and some near the test with a few in between	☐	4
Few at the beginning and few near the test with most in between	☐	5
Other (*please specify*)	☐	6

____

B14 When you were preparing for the practical driving test you took in the week beginning Monday 29th April 2002:

		Yes	No
**(a)**	Was it hard to find a friend or relation to take you out for driving practice?	☐	☐
**(b)**	Were you able to have as much driving practice with friends or relations as you wanted?	☐	☐
**(c)**	Was it difficult to find time to fit in driving practice with friends or relations?	☐	☐
**(d)**	Did the cost of professional lessons affect the number you took?	☐	☐
**(e)**	Do you feel that you had professional lessons often enough?	☐	☐
**(f)**	Was it difficult to find time to fit in professional lessons?	☐	☐
**(g)**	When you applied for the test did you think you had a good chance of passing it?	☐	☐
**(h)**	When you took the test did you think you had a good chance of passing it?	☐	☐
**(i)**	Were you keen to take your test as soon as possible?	☐	☐
**(j)**	Did you find learning to drive easy?	☐	☐

SECTION C: YOUR THEORY TEST

C1 When did you pass your theory test for car drivers?

_______________ month _______________ year

C2 Was this the first time you had taken the theory test for car drivers?

Yes ☐ ➡ ***Go to C4***

No ☐

C3 (a) In total, how many times have you taken the theory test for car drivers?

_______________ times

(b) When did you take your FIRST theory test for car drivers?

_______________ month _______________ year

PLEASE ANSWER QUESTIONS C4 TO C7 ABOUT THE FIRST THEORY TEST THAT YOU TOOK

C4 Had you driven at all before taking the theory test?

Yes ☐

No ☐ ➡ ***Go to C6***

C5 How many hours driving experience had you had in total when you took your first theory test?
Please include both professional lessons and driving practice with friends or relations.

_______________ **hours**

C6 In your opinion, how ready to apply for your practical driving test were you when you took your theory test?

(Tick ONE box only)

I was ready to apply for my practical driving test ☐

I was nearly ready to apply for my practical driving test ☐

I was not at all ready to apply for my practical driving test ☐

C7 Which of the following did your professional driving instructor do...

	(i) when preparing for your theory test	(ii) between your theory test and practical test
	(Tick ALL that apply)	
Taught or tested you on knowledge of road signs and markings	☐ 1	☐ 1
Taught or tested you on other driving- related knowledge	☐ 2	☐ 2
Taught or tested you on hazard awareness skills	☐ 3	☐ 3
Recommended particular driving-related books or materials	☐ 4	☐ 4
Went through example theory test questions	☐ 5	☐ 5
Did not have any lessons with a professional driving instructor	☐ 6	☐ 6
Other (*please specify*) ______________________	☐ 7	☐ 7
Nothing	☐ 8	☐ 8

C8 Which of the following did you use when preparing for your theory and practical driving tests?

	(i) Theory test	(ii) Practical test
	(Tick ALL that apply)	
DSA 'Official theory Test for Car Drivers'	☐ 1	☐ 1
Other example questions	☐ 2	☐ 2
DSA 'The Driving Manual'	☐ 3	☐ 3
Highway code	☐ 4	☐ 4
Any other driving related text book	☐ 5	☐ 5
Driving related CD-Rom	☐ 6	☐ 6
Driving related videos	☐ 7	☐ 7
Internet website	☐ 8	☐ 8
Other (*please specify*) ______________________	☐ 9	☐ 9
I did not use any material to prepare for the test	☐ 10	☐ 10

SECTION D: ATTITUDES TOWARDS DRIVING

D1 The following questions ask about your confidence in your own driving ability.

(Please tick ONE box per line)

	Very confident	Fairly confident	Not very confident	Not at all confident
(a) How confident were you in your driving ability when you were learning to drive?				
(b) In the week before you took your practical driving test, how confident were you that you would pass?				
(c) In the week before you took your practical driving test, how confident were you that you were a good enough driver to drive on the road unsupervised?				
(d) How confident are you in your driving ability now?				

D2 How much do you think you need to improve your ability on each of the following driving skills?

(Tick ONE box per line)

	No improvement needed	Some improvement needed	A lot of improvement needed
(a) Use of car controls			
(b) Pulling out of junctions			
(c) Reversing			
(d) Parking			
(e) Judging the speed of other traffic			
(f) Judging what other drivers are going to do			
(g) Spotting hazards			
(h) Driving in heavy traffic			
(i) Driving in the dark			
(j) Overtaking			
(k) Using roundabouts			
(l) Joining with moving traffic on a motorway or fast dual carriageway			
(m) Changing lanes on a motorway or fast dual carriageway			
(n) Driving on high speed roads			
(o) Driving on country roads			

D3 How much do you agree or disagree with each of the following statements?

(Tick ONE box on each line)

		Strongly agree (1)	Agree (2)	Neither agree nor disagree (3)	Disagree (4)	Strongly disagree (5)
(a)	Decreasing the motorway speed limit is a good idea					
(b)	Even at night-time on quiet roads it is important to keep within the speed limit					
(c)	Drivers who cause accidents by reckless driving should be banned from driving for life					
(d)	People should drive slower than the speed limit when it is raining					
(e)	Cars should never overtake on the inside lane even if a slow driver is blocking the outside lane					
(f)	In towns where there are a lot of pedestrians the speed limit should be 20mph					
(g)	Penalties for speeding should be more severe					
(h)	Increasing the motorway speed limit is a good idea					

D4 We would like to know what kind of driver you think you are by putting a tick somewhere on each of the lines below.

At either end of each line there is a word that describes a way of driving, and these words are opposites. Put your tick nearer to the word that best describes your driving. The closer your tick is to the word, the more you agree with this description of the way you drive.

D5

	(1)	(2)	(3)	(4)	(5)	(6)	(7)		
Attentive	a							Inattentive	1
Careful	b							Careless	1
Decisive	c							Indecisive	3
Experienced	d							Inexperienced	3
Irritable	e							Placid	-2
Nervous	f							Confident	-3
Patient	g							Impatient	2
Responsible	h							Irresponsible	1
Safe	i							Risky	1
Selfish	j							Considerate	-2
Slow	k							Fast	-3
Tolerant	l							Intolerant	2

SECTION E: CONTACT DETAILS

For the success of this important research, we would like to get in touch with you again to see how your driving is going.

Please fill in as many of the contact details below as possible.

All information will be treated in the STRICTEST CONFIDENCE and will not be used for any purpose other than to get in touch with you in relation to this research.

E1 Home phone number: ______________________________

E2 Work phone number: ______________________________
(*if applicable*)

E3 Mobile phone number: ______________________________

E4 Email address: ______________________________

If the address that this questionnaire was sent to is incorrect, or if there is a better address to help us contact you in the future, please provide the new address in the space below.

E5 New address: ______________________________

____

____

Postcode: ______________________________

E6 If you have any further comments to make about this survey and the issues raised, please write in the space provided:

E7 Please now return this questionnaire in the pre-paid envelope to:
NFER, The Mere, Upton Park, Slough, Berkshire, SL1 2DQ

Please return this questionnaire even if you have only answered the first few questions, as this information is equally important to us and will allow us to remove your name from our mailing list for future questionnaires.

Thank you very much for your help.

LEARNING TO DRIVE QUESTIONNAIRE

Please complete this questionnaire by ticking the appropriate boxes and filling in the spaces as required. Several questions ask for dates or lengths of time etc, if you are unable to give an exact answer to any of these questions please give as good an estimate as you can. It will only take a few minutes to complete this questionnaire and any information you provide will be treated in the **strictest confidence** and will be used for statistical purposes only.

SECTION A: YOUR PRACTICAL DRIVING TEST

A1 Did you take your practical driving test in the week beginning Monday 5th August 2002?

Yes ☐ ➡ ***Go to A2***

No ☐ ➡ ***Go to E7***

A2 When you took your practical driving test in the week beginning Monday 5th August 2002 what type of licence did you hold?

(Tick ONE box only)

Provisional licence to drive a car ☐ ➡ ***Go to A3***

Full licence for another class of vehicle ☐ ➡ ***Go to E7***

A3 Had you previously held a FULL (GB or non- GB) car driving licence before taking your practical driving test in the week beginning Monday 5th August 2002?

(Tick ONE box only)

NO ☐ ➡ ***Go to A4***

YES – Full GB licence ☐ ➡ ***Go to E7***

YES – Full non- GB licence ☐ ➡ ***Go to E7***

For offic use or

A4 How old were you when you got your first provisional car licence?

_______________ years _______________ months

A5 Before you started learning to drive a car did you regularly ride a motorcycle, moped, or scooter?

Yes ☐

No ☐

A6 Did you take your practical driving test in the week beginning Monday 5th August 2002 in:
(Tick ONE box only)

A driving instructor's car? ☐ 1

A car owned by a friend/relation? ☐ 2

Your own car? ☐ 3

Another car? ☐ 4

A7 Was this practical driving test your:
(Tick ONE box only)

First? ☐ 1 ➨ ***Go to A9***

Second? ☐ 2

Third? ☐ 3

Fourth? ☐ 4

More than fourth? ☐ 5

A8 When did you fail the practical driving test before the one in the week beginning Monday 5th August 2002?

Day_______________ Month _______________ Year _______________

A9 Did you pass the practical driving test that you took in the week beginning Monday 5th August 2002?

Yes ☐ ➨ ***Go to B1***

No ☐

A10 Did you agree with the Examiner's decision to fail you?

Yes ☐

No ☐

A11 Why do you think you failed on this occasion? ***(Tick ALL that apply)***

You were just 'unlucky' on the day	☐	1
Difficult weather conditions on the day of the test	☐	2
You were not ready for the test	☐	3
Nerves affected your performance	☐	4
Another road user was to blame	☐	5
Your driving was not good enough on the day	☐	6
You could not cope with something unexpected that happened	☐	7
Other (please specify)	☐	8

____

A12 Since failing the practical driving test in the week beginning Monday 5th August 2002, have you applied for another test?

Yes ☐

No ☐

A13 Before your next test do you think that you will... (***Tick ONE box only***)

Have professional driving lessons MORE OFTEN?	☐	1
Have professional driving lessons AS OFTEN AS BEFORE?	☐	2
Have professional driving lessons LESS OFTEN?	☐	3
START having professional driving lessons?	☐	4
NOT HAVE ANY professional driving lessons?	☐	5

A14 Before your next test do you think that you will... (***Tick ONE box only***)

Have practice driving sessions with friends or relations MORE OFTEN?	☐	1
Have practice driving sessions with friends or relations AS OFTEN AS BEFORE?	☐	2
Have practice driving sessions with friends or relations LESS OFTEN?	☐	3
START having practice driving sessions with friends or relations?	☐	4
NOT HAVE ANY practice driving sessions with friends or relations?	☐	5

For offic use o

SECTION B: LEARNING TO DRIVE

B1 When did you begin to learn to drive a car?

Month ______________________________ Year _______________

B2 Since you started learning to drive, was there any period of longer than 6 months during which you did no driving at all?

Yes ☐

No ☐ ➡ ***Go to B4***

B3 What was the total of all those periods during which you did no driving at all since you started learning to drive?

_______________ years _______________ months

B4 How many miles IN TOTAL had you driven a car before you took your practical driving test in the week beginning Monday 5th August 2002?

(If you are not sure, please give your best estimate to the nearest 100 miles)

_______________ miles

B5 Please estimate how your total driving time was split. ***(Tick ONE box only)***

All in a DRIVING INSTRUCTOR'S car	Almost all in a DRIVING INSTRUCTOR'S car	More than half in a DRIVING INSTRUCTOR'S car	About half and half	More than half in OTHER car(s)	Almost all in OTHER car(s)	All in OTHER car(s)
(1)	(2)	(3)	(4)	(5)	(6)	(7)
☐	☐	☐	☐	☐	☐	☐

B6 How many hours of lessons from a professional driving instructor have you had:

(a) IN TOTAL since you first started learning to drive?

_______________ hours **None** ☐ ➡ ***If none, please go to B11***

(b) Between the practical driving test that you took in the week beginning Monday 5th August 2002 and your previous test?

_______________ hours No previous practical driving test ☐

B7 How many different professional driving instructors have you ever had lessons from?

_______________ (Please write number of instructors)

B8 (a) Was your most recent professional driving instructor a DSA Approved Driving Instructor (ADI)?

Yes ☐
No ☐
Don't know ☐

(b) What grade of instructor is he or she? ***(Tick ONE box only)***

Grade 6 ADI ☐ 1
Grade 5 ADI ☐ 2
Grade 4 ADI ☐ 3
Trainee ADI ☐ 4
Don't know ☐ 5

(c) What was your main reason for choosing this instructor/driving school?

(Tick ONE box only)

Good reputation ☐ 1
Personal recommendation ☐ 2
You already knew the person ☐ 3
Cost of lessons ☐ 4
Local advertising ☐ 5
Large company/organisation ☐ 6
You saw them in the phone book ☐ 7
Highly qualified instructors ☐ 8
Other (*please specify*) ☐ 9

____

B9 Thinking about ALL the professional driving lessons that you took from when you started driving up to the time of your driving test in the week beginning Monday 5th August 2002, how much have you driven...

(Tick ONE box per line)

	**Never**	**Less than 2 hours**	**2-4 hours**	**More than 4 hours**
**(a)** In a busy town centre	☐	☐	☐	☐
**(b)** On country roads	☐	☐	☐	☐
**(c)** On fast dual carriageways	☐	☐	☐	☐
**(d)** In the dark	☐	☐	☐	☐
**(e)** In the rain	☐	☐	☐	☐
**(f)** On snow or ice	☐	☐	☐	☐

B10 When you were preparing for the practical driving test you took in the week beginning Monday 5th August 2002, how did you spread out your professional lessons?

(*Tick ONE box only*)

Regularly up to the practical driving test just taken	☐	1
Most at the beginning with a few near the test	☐	2
Most near the test with a few at the beginning	☐	3
Some at the beginning and some near the test with a few in between	☐	4
Few at the beginning and few near the test with most in between	☐	5
Other (*please specify*)	☐	6

____

B11 How many hours did you spend practising your driving with friends or relations?

(a) None ☐ ➨ ***If none, please go to B14***

(b) IN TOTAL since you first started learning to drive?

__________ hours

(c) Between the practical driving test that you took in the week beginning Monday 5th August 2002 and your previous car driving test?

__________ hours No previous practical driving test ☐

B12 Thinking about ALL the practice driving with friends and relatives that you did from when you started driving up to the time of your driving test in the week beginning Monday 5th August 2002, how much have you driven...

(Tick ONE box per line)

	Never	Less than 2 hours	2-4 hours	More than 4 hours
**(a)** In a busy town centre	☐	☐	☐	☐
**(b)** On country roads	☐	☐	☐	☐
**(c)** On fast dual carriageways	☐	☐	☐	☐
**(d)** In the dark	☐	☐	☐	☐
**(e)** In the rain	☐	☐	☐	☐
**(f)** On snow or ice	☐	☐	☐	☐

For office use only

B13 When you were preparing for the practical driving test you took in the week beginning Monday 5th August 2002, how did you spread out your sessions of driving practice with friends and relations?

(***Tick ONE box only***)

Regularly up to the practical driving test just taken	☐	1
Most at the beginning with a few near the test	☐	2
Most near the test with a few at the beginning	☐	3
Some at the beginning and some near the test with a few in between	☐	4
Few at the beginning and few near the test with most in between	☐	5
Other (*please specify*)	☐	6

____

B14 When you were preparing for the practical driving test you took in the week beginning Monday 5th August 2002:

		**Yes**	**No**
**(a)**	Was it hard to find a friend or relation to take you out for driving practice?	☐	☐
**(b)**	Were you able to have as much driving practice with friends or relations as you wanted?	☐	☐
**(c)**	Was it difficult to find time to fit in driving practice with friends or relations?	☐	☐
**(d)**	Did the cost of professional lessons affect the number you took?	☐	☐
**(e)**	Do you feel that you had professional lessons often enough?	☐	☐
**(f)**	Was it difficult to find time to fit in professional lessons?	☐	☐
**(g)**	When you applied for the test did you think you had a good chance of passing it?	☐	☐
**(h)**	When you took the test did you think you had a good chance of passing it?	☐	☐
**(i)**	Were you keen to take your test as soon as possible?	☐	☐
**(j)**	Did you find learning to drive easy?	☐	☐

SECTION C: YOUR THEORY TEST

C1 When did you pass your theory test for car drivers?

________________ month ________ year

C2 Was this the first time you had taken the theory test for car drivers?

Yes ☐ ➡ ***Go to C4***

No ☐

C3 (a) In total, how many times have you taken the theory test for car drivers?

________ times

(b) When did you take your FIRST theory test for car drivers?

________________ month ________ year

PLEASE ANSWER QUESTIONS C4 TO C7 ABOUT THE FIRST THEORY TEST THAT YOU TOOK

C4 Had you driven at all before taking the theory test?

Yes ☐

No ☐ ➡ ***Go to C6***

C5 How many hours driving experience had you had in total when you took your first theory test?
Please include both professional lessons and driving practice with friends or relations.

________ **hours**

C6 In your opinion, how ready to apply for your practical driving test were you when you took your theory test?

(Tick ONE box only)

I was ready to apply for my practical driving test ☐

I was nearly ready to apply for my practical driving test ☐

I was not at all ready to apply for my practical driving test ☐

C7 Which of the following did your professional driving instructor do…

	(i) when preparing for your theory test	(ii) between your theory test and practical test
	(Tick ALL that apply)	
Taught or tested you on knowledge of road signs and markings	☐ 1	☐ 1
Taught or tested you on other driving- related knowledge	☐ 2	☐ 2
Taught or tested you on hazard awareness skills	☐ 3	☐ 3
Recommended particular driving-related books or materials	☐ 4	☐ 4
Went through example theory test questions	☐ 5	☐ 5
Did not have any lessons with a professional driving instructor	☐ 6	☐ 6
Other (*please specify*) ______________________	☐ 7	☐ 7
Nothing	☐ 8	☐ 8

C8 Which of the following did you use when preparing for your theory and practical driving tests?

	(i) Theory test	(ii) Practical test
	(Tick ALL that apply)	
DSA 'Official theory Test for Car Drivers'	☐ 1	☐ 1
Other example questions	☐ 2	☐ 2
DSA 'The Driving Manual'	☐ 3	☐ 3
Highway code	☐ 4	☐ 4
Any other driving related text book	☐ 5	☐ 5
Driving related CD-Rom	☐ 6	☐ 6
Driving related videos	☐ 7	☐ 7
Internet website	☐ 8	☐ 8
Other (*please specify*) ______________________	☐ 9	☐ 9
I did not use any material to prepare for the test	☐ 10	☐ 10

For office use only

SECTION D: ATTITUDES TOWARDS DRIVING

D1 The following questions ask about your confidence in your own driving ability.

(***Please tick ONE box per line***)

	Very confident	Fairly confident	Not very confident	Not at all confident
**(a)** How confident were you in your driving ability when you were learning to drive?	☐	☐	☐	☐
**(b)** In the week before you took your practical driving test, how confident were you that you would pass?	☐	☐	☐	☐
**(c)** In the week before you took your practical driving test, how confident were you that you were a good enough driver to drive on the road unsupervised?	☐	☐	☐	☐
**(d)** How confident are you in your driving ability now?	☐	☐	☐	☐

D2 How much do you think you need to improve your ability on each of the following driving skills?

(Tick ONE box per line)

	No improvement needed	Some improvement needed	A lot of improvement needed
**(a)** Use of car controls	☐	☐	☐
**(b)** Pulling out of junctions	☐	☐	☐
**(c)** Reversing	☐	☐	☐
**(d)** Parking	☐	☐	☐
**(e)** Judging the speed of other traffic	☐	☐	☐
**(f)** Judging what other drivers are going to do	☐	☐	☐
**(g)** Spotting hazards	☐	☐	☐
**(h)** Driving in heavy traffic	☐	☐	☐
**(i)** Driving in the dark	☐	☐	☐
**(j)** Overtaking	☐	☐	☐
**(k)** Using roundabouts	☐	☐	☐
**(l)** Joining with moving traffic on a motorway or fast dual carriageway	☐	☐	☐
**(m)** Changing lanes on a motorway or fast dual carriageway	☐	☐	☐
**(n)** Driving on high speed roads	☐	☐	☐
**(o)** Driving on country roads	☐	☐	☐

D3 How much do you agree or disagree with each of the following statements?

(*Tick ONE box on each line*)

		Strongly agree (1)	Agree (2)	Neither agree nor disagree (3)	Disagree (4)	Strongly disagree (5)
(a)	Decreasing the motorway speed limit is a good idea					
(b)	Even at night-time on quiet roads it is important to keep within the speed limit					
(c)	Drivers who cause accidents by reckless driving should be banned from driving for life					
(d)	People should drive slower than the speed limit when it is raining					
(e)	Cars should never overtake on the inside lane even if a slow driver is blocking the outside lane					
(f)	In towns where there are a lot of pedestrians the speed limit should be 20mph					
(g)	Penalties for speeding should be more severe					
(h)	Increasing the motorway speed limit is a good idea					

D4 We would like to know what kind of driver you think you are by putting a tick somewhere on each of the lines below.

At either end of each line there is a word that describes a way of driving, and these words are opposites. Put your tick nearer to the word that best describes your driving. The closer your tick is to the word, the more you agree with this description of the way you drive.

	(1)	(2)	(3)	(4)	(5)	(6)	(7)	
Attentive								Inattentive
Careful								Careless
Decisive								Indecisive
Experienced								Inexperienced
Irritable								Placid
Nervous								Confident
Patient								Impatient
Responsible								Irresponsible
Safe								Risky
Selfish								Considerate
Slow								Fast
Tolerant								Intolerant

SECTION E: CONTACT DETAILS

For the success of this important research, we would like to get in touch with you again to see how your driving is going.

Please fill in as many of the contact details below as possible.

All information will be treated in the STRICTEST CONFIDENCE and will not be used for any purpose other than to get in touch with you in relation to this research.

E1 Home phone number: ______________________

E2 Work phone number: ______________________
(*if applicable*)

E3 Mobile phone number: ______________________

E4 Email address: ______________________

If the address that this questionnaire was sent to is incorrect, or if there is a better address to help us contact you in the future, please provide the new address in the space below.

E5 New address: ______________________

____

____

Postcode: ______________________

E6 If you have any further comments to make about this survey and the issues raised, please write in the space provided:

E7 Please now return this questionnaire in the pre-paid envelope to:
NFER, The Mere, Upton Park, Slough, Berkshire, SL1 2DQ

Please return this questionnaire even if you have only answered the first few questions, as this information is equally important to us and will allow us to remove your name from our mailing list for future questionnaires.

Thank you very much for your help.

Department for Transport | DSA | TRL

LEARNING TO DRIVE QUESTIONNAIRE

Please complete this questionnaire by ticking the appropriate boxes and filling in the spaces as required. Several questions ask for dates or lengths of time etc, if you are unable to give an exact answer to any of these questions please give as good an estimate as you can. It will only take a few minutes to complete this questionnaire and any information you provide will be treated in the **strictest confidence** and will be used for statistical purposes only.

SECTION A: YOUR PRACTICAL DRIVING TEST

A1 Did you take your practical driving test in the week beginning Monday 4th November 2002?

Yes ☐ ➡ ***Go to A2***

No ☐ ➡ ***Go to E7***

A2 When you took your practical driving test in the week beginning Monday 4th November 2002 what type of licence did you hold?

(Tick ONE box only)

Provisional licence to drive a car ☐ ➡ ***Go to A3***

Full licence for another class of vehicle ☐ ➡ ***Go to E7***

A3 Had you previously held a FULL (GB or non- GB) car driving licence before taking your practical driving test in the week beginning Monday 4th November 2002?

(Tick ONE box only)

NO ☐ ➡ ***Go to A4***

YES – Full GB licence ☐ ➡ ***Go to E7***

YES – Full non- GB licence ☐ ➡ ***Go to E7***

For office use only

A4 How old were you when you got your first provisional car licence?

____________ years ____________ months

A5 Before you started learning to drive a car did you regularly ride a motorcycle, moped, or scooter?

Yes ☐

No ☐

A6 Did you take your practical driving test in the week beginning Monday 4th November 2002 in:
(Tick ONE box only)

A driving instructor's car? ☐ 1

A car owned by a friend/relation? ☐ 2

Your own car? ☐ 3

Another car? ☐ 4

A7 Was this practical driving test your:
(Tick ONE box only)

First? ☐ 1 ➡ ***Go to A9***

Second? ☐ 2

Third? ☐ 3

Fourth? ☐ 4

More than fourth? ☐ 5

A8 When did you fail the practical driving test before the one in the week beginning Monday 4th November 2002?

Day____________ Month ____________________ Year ____________

A9 Did you pass the practical driving test that you took in the week beginning Monday 4th November 2002?

Yes ☐ ➡ ***Go to B1***

No ☐

A10 Did you agree with the Examiner's decision to fail you?

Yes ☐

No ☐

For office use only

A11 Why do you think you failed on this occasion? ***(Tick ALL that apply)***

You were just 'unlucky' on the day	☐	1
Difficult weather conditions on the day of the test	☐	2
You were not ready for the test	☐	3
Nerves affected your performance	☐	4
Another road user was to blame	☐	5
Your driving was not good enough on the day	☐	6
You could not cope with something unexpected that happened	☐	7
Other (please specify)	☐	8

____

A12 Since failing the practical driving test in the week beginning Monday 4th November 2002, have you applied for another test?

Yes ☐

No ☐

A13 Before your next test do you think that you will... (***Tick ONE box only***)

Have professional driving lessons MORE OFTEN?	☐	1
Have professional driving lessons AS OFTEN AS BEFORE?	☐	2
Have professional driving lessons LESS OFTEN?	☐	3
START having professional driving lessons?	☐	4
NOT HAVE ANY professional driving lessons?	☐	5

A14 Before your next test do you think that you will... (***Tick ONE box only***)

Have practice driving sessions with friends or relations MORE OFTEN?	☐	1
Have practice driving sessions with friends or relations AS OFTEN AS BEFORE?	☐	2
Have practice driving sessions with friends or relations LESS OFTEN?	☐	3
START having practice driving sessions with friends or relations?	☐	4
NOT HAVE ANY practice driving sessions with friends or relations?	☐	5

For office use only

SECTION B: LEARNING TO DRIVE

B1 When did you begin to learn to drive a car?

Month ______________________ Year ______________

B2 Since you started learning to drive, was there any period of longer than 6 months during which you did no driving at all?

Yes ☐

No ☐ ➡ ***Go to B4***

B3 What was the total of all those periods during which you did no driving at all since you started learning to drive?

______________ years ______________ months

B4 How many miles IN TOTAL had you driven a car before you took your practical driving test in the week beginning Monday 4th November 2002?

(If you are not sure, please give your best estimate to the nearest 100 miles)

______________ miles

B5 Please estimate how your total driving time was split. ***(Tick ONE box only)***

All in a DRIVING INSTRUCTOR'S car	Almost all in a DRIVING INSTRUCTOR'S car	More than half in a DRIVING INSTRUCTOR'S car	About half and half	More than half in OTHER car(s)	Almost all in OTHER car(s)	All in OTHER car(s)
(1)	(2)	(3)	(4)	(5)	(6)	(7)
☐	☐	☐	☐	☐	☐	☐

B6 How many hours of lessons from a professional driving instructor have you had:

(a) IN TOTAL since you first started learning to drive?

______________ hours **None** ☐ ➡ ***If none, please go to B11***

(b) Between the practical driving test that you took in the week beginning Monday 4th November 2002 and your previous test?

______________ hours No previous practical driving test ☐

B7 How many different professional driving instructors have you ever had lessons from?

______________ (Please write number of instructors)

For office use only

B8 (a) Was your most recent professional driving instructor a DSA Approved Driving Instructor (ADI)?

Yes ☐
No ☐
Don't know ☐

(b) What grade of instructor is he or she? ***(Tick ONE box only)***

Grade 6 ADI ☐ 1
Grade 5 ADI ☐ 2
Grade 4 ADI ☐ 3
Trainee ADI ☐ 4
Don't know ☐ 5

(c) What was your main reason for choosing this instructor/driving school?

(Tick ONE box only)

Good reputation ☐ 1
Personal recommendation ☐ 2
You already knew the person ☐ 3
Cost of lessons ☐ 4
Local advertising ☐ 5
Large company/organisation ☐ 6
You saw them in the phone book ☐ 7
Highly qualified instructors ☐ 8
Other (*please specify*) ☐ 9

____

B9 Thinking about ALL the professional driving lessons that you took from when you started driving up to the time of your driving test in the week beginning Monday 4th November 2002, how much have you driven...

(Tick ONE box per line)

	Never	Less than 2 hours	2-4 hours	More than 4 hours
**(a)** In a busy town centre	☐	☐	☐	☐
**(b)** On country roads	☐	☐	☐	☐
**(c)** On fast dual carriageways	☐	☐	☐	☐
**(d)** In the dark	☐	☐	☐	☐
**(e)** In the rain	☐	☐	☐	☐
**(f)** On snow or ice	☐	☐	☐	☐

For office use only

B10 When you were preparing for the practical driving test you took in the week beginning Monday 4th November 2002, how did you spread out your professional lessons?
(*Tick ONE box only*)

- Regularly up to the practical driving test just taken ☐ 1
- Most at the beginning with a few near the test ☐ 2
- Most near the test with a few at the beginning ☐ 3
- Some at the beginning and some near the test with a few in between ☐ 4
- Few at the beginning and few near the test with most in between ☐ 5
- Other (*please specify*) ☐ 6

____

B11 How many hours did you spend practising your driving with friends or relations?

(a) None ☐ ➨ ***If none, please go to B14***

(b) IN TOTAL since you first started learning to drive?

____________ hours

(c) Between the practical driving test that you took in the week beginning Monday 4th November 2002 and your previous car driving test?

____________ hours No previous practical driving test ☐

B12 Thinking about ALL the practice driving with friends and relatives that you did from when you started driving up to the time of your driving test in the week beginning Monday 4th November 2002, how much have you driven...
(Tick ONE box per line)

	Never	Less than 2 hours	2-4 hours	More than 4 hours
**(a)** In a busy town centre	☐	☐	☐	☐
**(b)** On country roads	☐	☐	☐	☐
**(c)** On fast dual carriageways	☐	☐	☐	☐
**(d)** In the dark	☐	☐	☐	☐
**(e)** In the rain	☐	☐	☐	☐
**(f)** On snow or ice	☐	☐	☐	☐

For office use only

B13 When you were preparing for the practical driving test you took in the week beginning Monday 4th November 2002, how did you spread out your sessions of driving practice with friends and relations?

(*Tick ONE box only*)

Regularly up to the practical driving test just taken	☐	1
Most at the beginning with a few near the test	☐	2
Most near the test with a few at the beginning	☐	3
Some at the beginning and some near the test with a few in between	☐	4
Few at the beginning and few near the test with most in between	☐	5
Other (*please specify*)	☐	6

____

B14 When you were preparing for the practical driving test you took in the week beginning Monday 4th November 2002:

		Yes	No
**(a)**	Was it hard to find a friend or relation to take you out for driving practice?	☐	☐
**(b)**	Were you able to have as much driving practice with friends or relations as you wanted?	☐	☐
**(c)**	Was it difficult to find time to fit in driving practice with friends or relations?	☐	☐
**(d)**	Did the cost of professional lessons affect the number you took?	☐	☐
**(e)**	Do you feel that you had professional lessons often enough?	☐	☐
**(f)**	Was it difficult to find time to fit in professional lessons?	☐	☐
**(g)**	When you applied for the test did you think you had a good chance of passing it?	☐	☐
**(h)**	When you took the test did you think you had a good chance of passing it?	☐	☐
**(i)**	Were you keen to take your test as soon as possible?	☐	☐
**(j)**	Did you find learning to drive easy?	☐	☐

For office use only

SECTION C: YOUR THEORY TEST

C1 When did you pass your theory test for car drivers?

________________ month ________ year

C2 Was this the first time you had taken the theory test for car drivers?

Yes ☐ ➡ ***Go to C4***

No ☐

C3 (a) In total, how many times have you taken the theory test for car drivers?

________ times

(b) When did you take your FIRST theory test for car drivers?

________________ month ________ year

PLEASE ANSWER QUESTIONS C4 TO C7 ABOUT THE <u>FIRST</u> THEORY TEST THAT YOU TOOK

C4 Had you driven at all before taking the theory test?

Yes ☐

No ☐ ➡ ***Go to C6***

C5 How many hours driving experience had you had in total when you took your first theory test?
Please include both professional lessons and driving practice with friends or relations.

________ **hours**

C6 In your opinion, how ready to apply for your practical driving test were you when you took your theory test?

(Tick ONE box only)

I was ready to apply for my practical driving test ☐

I was nearly ready to apply for my practical driving test ☐

I was not at all ready to apply for my practical driving test ☐

For office use only

C7 Which of the following did your professional driving instructor do…

	(i) when preparing for your theory test	(ii) between your theory test and practical test
	(Tick ALL that apply)	
Taught or tested you on knowledge of road signs and markings	☐ 1	☐ 1
Taught or tested you on other driving- related knowledge	☐ 2	☐ 2
Taught or tested you on hazard awareness skills	☐ 3	☐ 3
Recommended particular driving-related books or materials	☐ 4	☐ 4
Went through example theory test questions	☐ 5	☐ 5
Did not have any lessons with a professional driving instructor	☐ 6	☐ 6
Other (*please specify*) ______________________	☐ 7	☐ 7
Nothing	☐ 8	☐ 8

C8 Which of the following did you use when preparing for your theory and practical driving tests?

	(i) Theory test	(ii) Practical test
	(Tick ALL that apply)	
DSA 'Official theory Test for Car Drivers'	☐ 1	☐ 1
Other example questions	☐ 2	☐ 2
DSA 'The Driving Manual'	☐ 3	☐ 3
Highway code	☐ 4	☐ 4
Any other driving related text book	☐ 5	☐ 5
Driving related CD-Rom	☐ 6	☐ 6
Driving related videos	☐ 7	☐ 7
Internet website	☐ 8	☐ 8
Other (*please specify*) ______________________	☐ 9	☐ 9
I did not use any material to prepare for the test	☐ 10	☐ 10

For office use only

SECTION D: ATTITUDES TOWARDS DRIVING

D1 The following questions ask about your confidence in your own driving ability.

(*Please tick ONE box per line*)

	Very confident	Fairly confident	Not very confident	Not at all confident
**(a)** How confident were you in your driving ability when you were learning to drive?	☐	☐	☐	☐
**(b)** In the week before you took your practical driving test, how confident were you that you would pass?	☐	☐	☐	☐
**(c)** In the week before you took your practical driving test, how confident were you that you were a good enough driver to drive on the road unsupervised?	☐	☐	☐	☐
**(d)** How confident are you in your driving ability now?	☐	☐	☐	☐

D2 How much do you think you need to improve your ability on each of the following driving skills?

(Tick ONE box per line)

	No improvement needed	Some improvement needed	A lot of improvement needed
**(a)** Use of car controls	☐	☐	☐
**(b)** Pulling out of junctions	☐	☐	☐
**(c)** Reversing	☐	☐	☐
**(d)** Parking	☐	☐	☐
**(e)** Judging the speed of other traffic	☐	☐	☐
**(f)** Judging what other drivers are going to do	☐	☐	☐
**(g)** Spotting hazards	☐	☐	☐
**(h)** Driving in heavy traffic	☐	☐	☐
**(i)** Driving in the dark	☐	☐	☐
**(j)** Overtaking	☐	☐	☐
**(k)** Using roundabouts	☐	☐	☐
**(l)** Joining with moving traffic on a motorway or fast dual carriageway	☐	☐	☐
**(m)** Changing lanes on a motorway or fast dual carriageway	☐	☐	☐
**(n)** Driving on high speed roads	☐	☐	☐
**(o)** Driving on country roads	☐	☐	☐

D3 How much do you agree or disagree with each of the following statements?

(Tick ONE box on each line)

		Strongly agree (1)	Agree (2)	Neither agree nor disagree (3)	Disagree (4)	Strongly disagree (5)
(a)	Decreasing the motorway speed limit is a good idea					
(b)	Even at night-time on quiet roads it is important to keep within the speed limit					
(c)	Drivers who cause accidents by reckless driving should be banned from driving for life					
(d)	People should drive slower than the speed limit when it is raining					
(e)	Cars should never overtake on the inside lane even if a slow driver is blocking the outside lane					
(f)	In towns where there are a lot of pedestrians the speed limit should be 20mph					
(g)	Penalties for speeding should be more severe					
(h)	Increasing the motorway speed limit is a good idea					

D4 We would like to know what kind of driver you think you are by putting a tick somewhere on each of the lines below.

At either end of each line there is a word that describes a way of driving, and these words are opposites. Put your tick nearer to the word that best describes your driving. The closer your tick is to the word, the more you agree with this description of the way you drive.

	(1)	(2)	(3)	(4)	(5)	(6)	(7)	
Attentive								Inattentive
Careful								Careless
Decisive								Indecisive
Experienced								Inexperienced
Irritable								Placid
Nervous								Confident
Patient								Impatient
Responsible								Irresponsible
Safe								Risky
Selfish								Considerate
Slow								Fast
Tolerant								Intolerant

SECTION E: CONTACT DETAILS

For the success of this important research, we would like to get in touch with you again to see how your driving is going.

Please fill in as many of the contact details below as possible.

All information will be treated in the STRICTEST CONFIDENCE and will not be used for any purpose other than to get in touch with you in relation to this research.

E1 Home phone number: ______________________

E2 Work phone number: ______________________
(*if applicable*)

E3 Mobile phone number: ______________________

E4 Email address: ______________________

If the address that this questionnaire was sent to is incorrect, or if there is a better address to help us contact you in the future, please provide the new address in the space below.

E5 New address: ______________________

____

____

Postcode: ______________________

E6 If you have any further comments to make about this survey and the issues raised, please write in the space provided:

E7 Please now return this questionnaire in the pre-paid envelope to:
NFER, The Mere, Upton Park, Slough, Berkshire, SL1 2DQ

Please return this questionnaire even if you have only answered the first few questions, as this information is equally important to us and will allow us to remove your name from our mailing list for future questionnaires.

Thank you very much for your help.

LEARNING TO DRIVE QUESTIONNAIRE

Please complete this questionnaire by ticking the appropriate boxes and filling in the spaces as required. Several questions ask for dates or lengths of time etc, if you are unable to give an exact answer to any of these questions please give as good an estimate as you can. It will only take a few minutes to complete this questionnaire and any information you provide will be treated in the **strictest confidence** and will be used for statistical purposes only.

SECTION A: YOUR PRACTICAL DRIVING TEST

A1 Did you take your practical driving test in the week beginning Monday 3rd February 2003?

Yes ☐ ➡ ***Go to A2***

No ☐ ➡ ***Go to E7***

A2 When you took your practical driving test in the week beginning Monday 3rd February 2003 what type of licence did you hold?

(Tick ONE box only)

Provisional licence to drive a car ☐ ➡ ***Go to A3***

Full licence for another class of vehicle ☐ ➡ ***Go to E7***

A3 Had you previously held a FULL (GB or non- GB) car driving licence before taking your practical driving test in the week beginning Monday 3rd February 2003?

(Tick ONE box only)

NO ☐ ➡ ***Go to A4***

YES – Full GB licence ☐ ➡ ***Go to E7***

YES – Full non- GB licence ☐ ➡ ***Go to E7***

For office use only

A4 How old were you when you got your first provisional car licence?

_______________ years _______________ months

A5 Before you started learning to drive a car did you regularly ride a motorcycle, moped, or scooter?

Yes ☐

No ☐

A6 Did you take your practical driving test in the week beginning Monday 3rd February 2003 in:
(Tick ONE box only)

A driving instructor's car? ☐ 1

A car owned by a friend/relation? ☐ 2

Your own car? ☐ 3

Another car? ☐ 4

A7 Was this practical driving test your:
(Tick ONE box only)

First? ☐ 1 ➡ ***Go to A9***

Second? ☐ 2

Third? ☐ 3

Fourth? ☐ 4

More than fourth? ☐ 5

A8 When did you fail the practical driving test before the one in the week beginning Monday 3rd February 2003?

Day_______________ Month ______________________________ Year _______________

A9 Did you pass the practical driving test that you took in the week beginning Monday 3rd February 2003?

Yes ☐ ➡ ***Go to B1***

No ☐

A10 Did you agree with the Examiner's decision to fail you?

Yes ☐

No ☐

A11 Why do you think you failed on this occasion? ***(Tick ALL that apply)***

You were just 'unlucky' on the day	☐	1
Difficult weather conditions on the day of the test	☐	2
You were not ready for the test	☐	3
Nerves affected your performance	☐	4
Another road user was to blame	☐	5
Your driving was not good enough on the day	☐	6
You could not cope with something unexpected that happened	☐	7
Other (please specify)	☐	8

____

A12 Since failing the practical driving test in the week beginning Monday 3rd February 2003, have you applied for another test?

Yes ☐

No ☐

A13 Before your next test do you think that you will... **(*Tick ONE box only*)**

Have professional driving lessons MORE OFTEN?	☐	1
Have professional driving lessons AS OFTEN AS BEFORE?	☐	2
Have professional driving lessons LESS OFTEN?	☐	3
START having professional driving lessons?	☐	4
NOT HAVE ANY professional driving lessons?	☐	5

A14 Before your next test do you think that you will... **(*Tick ONE box only*)**

Have practice driving sessions with friends or relations MORE OFTEN?	☐	1
Have practice driving sessions with friends or relations AS OFTEN AS BEFORE?	☐	2
Have practice driving sessions with friends or relations LESS OFTEN?	☐	3
START having practice driving sessions with friends or relations?	☐	4
NOT HAVE ANY practice driving sessions with friends or relations?	☐	5

SECTION B: LEARNING TO DRIVE

B1 When did you begin to learn to drive a car?

Month ______________________________ Year _______________

B2 Since you started learning to drive, was there any period of longer than 6 months during which you did no driving at all?

Yes ☐

No ☐ ➡ ***Go to B4***

B3 What was the total of all those periods during which you did no driving at all since you started learning to drive?

_______________ years _______________ months

B4 How many miles IN TOTAL had you driven a car before you took your practical driving test in the week beginning Monday 3rd February 2003?

(If you are not sure, please give your best estimate to the nearest 100 miles)

_______________ miles

B5 Please estimate how your total driving time was split. ***(Tick ONE box only)***

All in a DRIVING INSTRUCTOR'S car	Almost all in a DRIVING INSTRUCTOR'S car	More than half in a DRIVING INSTRUCTOR'S car	About half and half	More than half in OTHER car(s)	Almost all in OTHER car(s)	All in OTHER car(s)
(1)	(2)	(3)	(4)	(5)	(6)	(7)
☐	☐	☐	☐	☐	☐	☐

B6 How many hours of lessons from a professional driving instructor have you had:

(a) IN TOTAL since you first started learning to drive?

_______________ hours **None** ☐ ➡ ***If none, please go to B11***

(b) Between the practical driving test that you took in the week beginning Monday 3rd February 2003 and your previous test?

_______________ hours No previous practical driving test ☐

B7 How many different professional driving instructors have you ever had lessons from?

_______________ (Please write number of instructors)

B8 (a) Was your most recent professional driving instructor a DSA Approved Driving Instructor (ADI)?

Yes ☐
No ☐
Don't know ☐

(b) What grade of instructor is he or she? ***(Tick ONE box only)***

Grade 6 ADI ☐ 1
Grade 5 ADI ☐ 2
Grade 4 ADI ☐ 3
Trainee ADI ☐ 4
Don't know ☐ 5

(c) What was your main reason for choosing this instructor/driving school?

(Tick ONE box only)

Good reputation ☐ 1
Personal recommendation ☐ 2
You already knew the person ☐ 3
Cost of lessons ☐ 4
Local advertising ☐ 5
Large company/organisation ☐ 6
You saw them in the phone book ☐ 7
Highly qualified instructors ☐ 8
Other (***please specify***) ☐ 9

____

B9 Thinking about ALL the professional driving lessons that you took from when you started driving up to the time of your driving test in the week beginning Monday 3rd February 2003, how much have you driven...

(Tick ONE box per line)

		Never	Less than 2 hours	2-4 hours	More than 4 hours
**(a)**	In a busy town centre	☐	☐	☐	☐
**(b)**	On country roads	☐	☐	☐	☐
**(c)**	On fast dual carriageways	☐	☐	☐	☐
**(d)**	In the dark	☐	☐	☐	☐
**(e)**	In the rain	☐	☐	☐	☐
**(f)**	On snow or ice	☐	☐	☐	☐

For office use only

B10 When you were preparing for the practical driving test you took in the week beginning Monday 3rd February 2003, how did you spread out your professional lessons?

(*Tick ONE box only*)

Regularly up to the practical driving test just taken	☐	1
Most at the beginning with a few near the test	☐	2
Most near the test with a few at the beginning	☐	3
Some at the beginning and some near the test with a few in between	☐	4
Few at the beginning and few near the test with most in between	☐	5
Other (***please specify***)	☐	6

____

B11 How many hours did you spend practising your driving with friends or relations?

(a) None ☐ ➨ ***If none, please go to B14***

(b) IN TOTAL since you first started learning to drive?

______________ hours

(c) Between the practical driving test that you took in the week beginning Monday 3rd February 2003 and your previous car driving test?

______________ hours No previous practical driving test ☐

B12 Thinking about ALL the practice driving with friends and relatives that you did from when you started driving up to the time of your driving test in the week beginning Monday 3rd February 2003, how much have you driven...

(Tick ONE box per line)

	Never	Less than 2 hours	2-4 hours	More than 4 hours
**(a)** In a busy town centre	☐	☐	☐	☐
**(b)** On country roads	☐	☐	☐	☐
**(c)** On fast dual carriageways	☐	☐	☐	☐
**(d)** In the dark	☐	☐	☐	☐
**(e)** In the rain	☐	☐	☐	☐
**(f)** On snow or ice	☐	☐	☐	☐

For office use only

B13 When you were preparing for the practical driving test you took in the week beginning Monday 3rd February 2003, how did you spread out your sessions of driving practice with friends and relations?

(*Tick ONE box only*)

Regularly up to the practical driving test just taken	☐	1
Most at the beginning with a few near the test	☐	2
Most near the test with a few at the beginning	☐	3
Some at the beginning and some near the test with a few in between	☐	4
Few at the beginning and few near the test with most in between	☐	5
Other (***please specify***)	☐	6

____

B14 When you were preparing for the practical driving test you took in the week beginning Monday 3rd February 2003:

		Yes	No
**(a)**	Was it hard to find a friend or relation to take you out for driving practice?	☐	☐
**(b)**	Were you able to have as much driving practice with friends or relations as you wanted?	☐	☐
**(c)**	Was it difficult to find time to fit in driving practice with friends or relations?	☐	☐
**(d)**	Did the cost of professional lessons affect the number you took?	☐	☐
**(e)**	Do you feel that you had professional lessons often enough?	☐	☐
**(f)**	Was it difficult to find time to fit in professional lessons?	☐	☐
**(g)**	When you applied for the test did you think you had a good chance of passing it?	☐	☐
**(h)**	When you took the test did you think you had a good chance of passing it?	☐	☐
**(i)**	Were you keen to take your test as soon as possible?	☐	☐
**(j)**	Did you find learning to drive easy?	☐	☐

For office use only

SECTION C: YOUR THEORY TEST

C1 When did you pass your theory test for car drivers?

____________________ month __________ year

C2 Did the theory test you passed include a Hazard Perception test using film clips?

Yes ☐

No ☐

C3 Was this the first time you had taken the theory test for car drivers?

Yes ☐ ➡ ***Go to C6***

No ☐

C4 (a) In total, how many times have you taken the theory test for car drivers?

__________ times

(b) When did you take your FIRST theory test for car drivers?

____________________ month __________ year

C5 Did the FIRST theory test you took include a Hazard Perception test using film clips?

Yes ☐

No ☐

PLEASE ANSWER QUESTIONS C6 TO C9 ABOUT THE FIRST THEORY TEST THAT YOU TOOK

C6 Had you driven at all before taking your first theory test?

Yes ☐

No ☐ ➡ ***Go to C8***

C7 How many hours driving experience had you had in total when you took your first theory test? Please include both professional lessons and driving practice with friends or relations.

__________ **hours**

C8 In your opinion, how ready to apply for your practical driving test were you when you took your theory test?

(Tick ONE box only)

I was ready to apply for my practical driving test ☐

I was nearly ready to apply for my practical driving test ☐

I was not at all ready to apply for my practical driving test ☐

C9 Which of the following did your professional driving instructor do…

(Tick ALL that apply)

	(i) when preparing for your theory test	(ii) between your theory test and practical test
Taught or tested you on knowledge of road signs and markings	1	1
Taught or tested you on other driving- related knowledge	2	2
Taught or tested you on hazard awareness skills	3	3
Recommended particular driving-related books or materials	4	4
Went through example theory test questions	5	5
Did not have any lessons with a professional driving instructor	6	6
Other (***please specify***) ______________________	7	7
Nothing	8	8

C10 Which of the following did you use when preparing for your theory and practical driving tests?

(Tick ALL that apply)

	(i) Theory test: Multiple Choice part	(i) Theory test: Hazard Perception part (if included in your theory test)	(ii) Practical test
DSA book 'Official theory Test for Car Drivers'	1	1	1
DSA book 'The Driving Manual'/'Driving-the essential skills'	2	2	2
DSA 'Roadsense'	3	3	3
Highway code	4	4	4
'Know your traffic signs' book	5	5	5
Other books or sets of example questions	6	6	6
An interactive video, CD or DVD based at a driving school	7	7	7
Other driving related video(s), CD(s) or DVD(s)	8	8	8
A website	9	9	9
Other (***please specify***) ______________________	10	10	10
I did not use any material to prepare for the test	11	11	11

For office use only

SECTION D: ATTITUDES TOWARDS DRIVING

D1 The following questions ask about your confidence in your own driving ability.

(*Please tick ONE box per line*)

	Very confident	Fairly confident	Not very confident	Not at all confident
**(a)** How confident were you in your driving ability when you were learning to drive?	☐	☐	☐	☐
**(b)** In the week before you took your practical driving test, how confident were you that you would pass?	☐	☐	☐	☐
**(c)** In the week before you took your practical driving test, how confident were you that you were a good enough driver to drive on the road unsupervised?	☐	☐	☐	☐
**(d)** How confident are you in your driving ability now?	☐	☐	☐	☐

D2 How much do you think you need to improve your ability on each of the following driving skills?

(Tick ONE box per line)

	No improvement needed	Some improvement needed	A lot of improvement needed
**(a)** Use of car controls	☐	☐	☐
**(b)** Pulling out of junctions	☐	☐	☐
**(c)** Reversing	☐	☐	☐
**(d)** Parking	☐	☐	☐
**(e)** Judging the speed of other traffic	☐	☐	☐
**(f)** Judging what other drivers are going to do	☐	☐	☐
**(g)** Spotting hazards	☐	☐	☐
**(h)** Driving in heavy traffic	☐	☐	☐
**(i)** Driving in the dark	☐	☐	☐
**(j)** Overtaking	☐	☐	☐
**(k)** Using roundabouts	☐	☐	☐
**(l)** Joining with moving traffic on a motorway or fast dual carriageway	☐	☐	☐
**(m)** Changing lanes on a motorway or fast dual carriageway	☐	☐	☐
**(n)** Driving on high speed roads	☐	☐	☐
**(o)** Driving on country roads	☐	☐	☐

D3 How much do you agree or disagree with each of the following statements?

(Tick ONE box on each line)

	Strongly agree (1)	Agree (2)	Neither agree nor disagree (3)	Disagree (4)	Strongly disagree (5)
**(a)** Decreasing the motorway speed limit is a good idea					
**(b)** Even at night-time on quiet roads it is important to keep within the speed limit					
**(c)** Drivers who cause accidents by reckless driving should be banned from driving for life					
**(d)** People should drive slower than the speed limit when it is raining					
**(e)** Cars should never overtake on the inside lane even if a slow driver is blocking the outside lane					
**(f)** In towns where there are a lot of pedestrians the speed limit should be 20mph					
**(g)** Penalties for speeding should be more severe					
**(h)** Increasing the motorway speed limit is a good idea					

D4 We would like to know what kind of driver you think you are by putting a tick some-where on each of the lines below.

At either end of each line there is a word that describes a way of driving, and these words are opposites. Put your tick nearer to the word that best describes your driving. The closer your tick is to the word, the more you agree with this description of the way you drive.

	(1)	(2)	(3)	(4)	(5)	(6)	(7)	
Attentive								Inattentive
Careful								Careless
Decisive								Indecisive
Experienced								Inexperienced
Irritable								Placid
Nervous								Confident
Patient								Impatient
Responsible								Irresponsible
Safe								Risky
Selfish								Considerate
Slow								Fast
Tolerant								Intolerant

SECTION E: CONTACT DETAILS

For the success of this important research, we would like to get in touch with you again to see how your driving is going.

Please fill in as many of the contact details below as possible.

All information will be treated in the STRICTEST CONFIDENCE and will not be used for any purpose other than to get in touch with you in relation to this research.

E1 Home phone number: ______________________________

E2 Work phone number: ______________________________
(*if applicable*)

E3 Mobile phone number: ______________________________

E4 Email address: ______________________________

If the address that this questionnaire was sent to is incorrect, or if there is a better address to help us contact you in the future, please provide the new address in the space below.

E5 New address: ______________________________

____

____

Postcode: ______________________________

E6 If you have any further comments to make about this survey and the issues raised, please write in the space provided:

E7 Please now return this questionnaire in the pre-paid envelope to:
NFER, The Mere, Upton Park, Slough, Berkshire, SL1 2DQ

Please return this questionnaire even if you have only answered the first few questions, as this information is equally important to us and will allow us to remove your name from our mailing list for future questionnaires.

Thank you very much for your help.

Department for Transport | DSA | TRL

LEARNING TO DRIVE QUESTIONNAIRE

Please complete this questionnaire by ticking the appropriate boxes and filling in the spaces as required. Several questions ask for dates or lengths of time etc, if you are unable to give an exact answer to any of these questions please give as good an estimate as you can. It will only take a few minutes to complete this questionnaire and any information you provide will be treated in the **strictest confidence** and will be used for statistical purposes only.

SECTION A: YOUR PRACTICAL DRIVING TEST

A1 Did you take your practical driving test in the week beginning Monday 28th April 2003?

Yes ☐ ➡ ***Go to A2***

No ☐ ➡ ***Go to E7***

A2 When you took your practical driving test in the week beginning Monday 28th April 2003 what type of licence did you hold?

(Tick ONE box only)

Provisional licence to drive a car ☐ ➡ ***Go to A3***

Full licence for another class of vehicle ☐ ➡ ***Go to E7***

A3 Had you previously held a FULL (GB or non- GB) car driving licence before taking your practical driving test in the week beginning Monday 28th April 2003?

(Tick ONE box only)

NO ☐ ➡ ***Go to A4***

YES – Full GB licence ☐ ➡ ***Go to E7***

YES – Full non- GB licence ☐ ➡ ***Go to E7***

For office use only

A4 How old were you when you got your first provisional car licence?

____________ years ____________ months

A5 Before you started learning to drive a car did you regularly ride a motorcycle, moped, or scooter?

Yes ☐

No ☐

A6 Did you take your practical driving test in the week beginning Monday 28th April 2003 in:
(Tick ONE box only)

A driving instructor's car? ☐ 1

A car owned by a friend/relation? ☐ 2

Your own car? ☐ 3

Another car? ☐ 4

A7 Was this practical driving test your:
(Tick ONE box only)

First? ☐ 1 ➡ ***Go to A9***

Second? ☐ 2

Third? ☐ 3

Fourth? ☐ 4

More than fourth? ☐ 5

A8 When did you fail the practical driving test before the one in the week beginning Monday 28th April 2003?

Day____________ Month ____________________ Year ____________

A9 Did you pass the practical driving test that you took in the week beginning Monday 28th April 2003?

Yes ☐ ➡ ***Go to B1***

No ☐

A10 Did you agree with the Examiner's decision to fail you?

Yes ☐

No ☐

For office use only

A11 Why do you think you failed on this occasion? ***(Tick ALL that apply)***

You were just 'unlucky' on the day	☐	1
Difficult weather conditions on the day of the test	☐	2
You were not ready for the test	☐	3
Nerves affected your performance	☐	4
Another road user was to blame	☐	5
Your driving was not good enough on the day	☐	6
You could not cope with something unexpected that happened	☐	7
Other (please specify)	☐	8

____

A12 Since failing the practical driving test in the week beginning Monday 28th April 2003, have you applied for another test?

Yes ☐

No ☐

A13 Before your next test do you think that you will... ***(Tick ONE box only)***

Have professional driving lessons MORE OFTEN?	☐	1
Have professional driving lessons AS OFTEN AS BEFORE?	☐	2
Have professional driving lessons LESS OFTEN?	☐	3
START having professional driving lessons?	☐	4
NOT HAVE ANY professional driving lessons?	☐	5

A14 Before your next test do you think that you will... ***(Tick ONE box only)***

Have practice driving sessions with friends or relations MORE OFTEN?	☐	1
Have practice driving sessions with friends or relations AS OFTEN AS BEFORE?	☐	2
Have practice driving sessions with friends or relations LESS OFTEN?	☐	3
START having practice driving sessions with friends or relations?	☐	4
NOT HAVE ANY practice driving sessions with friends or relations?	☐	5

For office use only

SECTION B: LEARNING TO DRIVE

B1 When did you begin to learn to drive a car?

Month ______________________ Year ___________

B2 Since you started learning to drive, was there any period of longer than 6 months during which you did no driving at all?

Yes ☐

No ☐ ➡ ***Go to B4***

B3 What was the total of all those periods during which you did no driving at all since you started learning to drive?

___________ years ___________ months

B4 How many miles IN TOTAL had you driven a car before you took your practical driving test in the week beginning Monday 28th April 2003?

(If you are not sure, please give your best estimate to the nearest 100 miles)

___________ miles

B5 Please estimate how your total driving time was split. ***(Tick ONE box only)***

All in a DRIVING INSTRUCTOR'S car	Almost all in a DRIVING INSTRUCTOR'S car	More than half in a DRIVING INSTRUCTOR'S car	About half and half	More than half in OTHER car(s)	Almost all in OTHER car(s)	All in OTHER car(s)
(1)	(2)	(3)	(4)	(5)	(6)	(7)
☐	☐	☐	☐	☐	☐	☐

B6 How many hours of lessons from a professional driving instructor have you had:

(a) IN TOTAL since you first started learning to drive?

___________ hours **None** ☐ ➡ ***If none, please go to B11***

(b) Between the practical driving test that you took in the week beginning Monday 28th April 2003 and your previous test?

___________ hours No previous practical driving test ☐

B7 How many different professional driving instructors have you ever had lessons from?

___________ (Please write number of instructors)

B8 (a) Was your most recent professional driving instructor a DSA Approved Driving Instructor (ADI)?

Yes ☐

No ☐

Don't know ☐

(b) What grade of instructor is he or she? ***(Tick ONE box only)***

Grade 6 ADI ☐ 1

Grade 5 ADI ☐ 2

Grade 4 ADI ☐ 3

Trainee ADI ☐ 4

Don't know ☐ 5

(c) What was your main reason for choosing this instructor/driving school?

(Tick ONE box only)

Good reputation ☐ 1

Personal recommendation ☐ 2

You already knew the person ☐ 3

Cost of lessons ☐ 4

Local advertising ☐ 5

Large company/organisation ☐ 6

You saw them in the phone book ☐ 7

Highly qualified instructors ☐ 8

Other (***please specify***) ☐ 9

____

B9 Thinking about ALL the professional driving lessons that you took from when you started driving up to the time of your driving test in the week beginning Monday 28th April 2003, how much have you driven...

(Tick ONE box per line)

		Never	Less than 2 hours	2-4 hours	More than 4 hours
**(a)**	In a busy town centre	☐	☐	☐	☐
**(b)**	On country roads	☐	☐	☐	☐
**(c)**	On fast dual carriageways	☐	☐	☐	☐
**(d)**	In the dark	☐	☐	☐	☐
**(e)**	In the rain	☐	☐	☐	☐
**(f)**	On snow or ice	☐	☐	☐	☐

For office use only

B10 When you were preparing for the practical driving test you took in the week beginning Monday 28th April 2003, how did you spread out your professional lessons?

(*Tick ONE box only*)

Regularly up to the practical driving test just taken	☐	1
Most at the beginning with a few near the test	☐	2
Most near the test with a few at the beginning	☐	3
Some at the beginning and some near the test with a few in between	☐	4
Few at the beginning and few near the test with most in between	☐	5
Other (***please specify***)	☐	6

____

B11 How many hours did you spend practising your driving with friends or relations?

(a) None ☐ ➨ ***If none, please go to B14***

(b) IN TOTAL since you first started learning to drive?

_____________ hours

(c) Between the practical driving test that you took in the week beginning Monday 28th April 2003 and your previous car driving test?

_____________ hours No previous practical driving test ☐

B12 Thinking about ALL the practice driving with friends and relatives that you did from when you started driving up to the time of your driving test in the week beginning Monday 28th April 2003, how much have you driven...

(Tick ONE box per line)

	Never	Less than 2 hours	2-4 hours	More than 4 hours
**(a)** In a busy town centre	☐	☐	☐	☐
**(b)** On country roads	☐	☐	☐	☐
**(c)** On fast dual carriageways	☐	☐	☐	☐
**(d)** In the dark	☐	☐	☐	☐
**(e)** In the rain	☐	☐	☐	☐
**(f)** On snow or ice	☐	☐	☐	☐

For office use only

B13 When you were preparing for the practical driving test you took in the week beginning Monday 28th April 2003, how did you spread out your sessions of driving practice with friends and relations?

(***Tick ONE box only***)

Regularly up to the practical driving test just taken	☐	1
Most at the beginning with a few near the test	☐	2
Most near the test with a few at the beginning	☐	3
Some at the beginning and some near the test with a few in between	☐	4
Few at the beginning and few near the test with most in between	☐	5
Other (***please specify***)	☐	6

____

B14 When you were preparing for the practical driving test you took in the week beginning Monday 28th April 2003:

		Yes	No
**(a)**	Was it hard to find a friend or relation to take you out for driving practice?	☐	☐
**(b)**	Were you able to have as much driving practice with friends or relations as you wanted?	☐	☐
**(c)**	Was it difficult to find time to fit in driving practice with friends or relations?	☐	☐
**(d)**	Did the cost of professional lessons affect the number you took?	☐	☐
**(e)**	Do you feel that you had professional lessons often enough?	☐	☐
**(f)**	Was it difficult to find time to fit in professional lessons?	☐	☐
**(g)**	When you applied for the test did you think you had a good chance of passing it?	☐	☐
**(h)**	When you took the test did you think you had a good chance of passing it?	☐	☐
**(i)**	Were you keen to take your test as soon as possible?	☐	☐
**(j)**	Did you find learning to drive easy?	☐	☐

For office use only

SECTION C: YOUR THEORY TEST

C1 When did you pass your theory test for car drivers?

_______________ month _______________ year

C2 Did the theory test you passed include a Hazard Perception test using film clips?

Yes ☐

No ☐

C3 Was this the first time you had taken the theory test for car drivers?

Yes ☐ ➡ ***Go to C6***

No ☐

C4 (a) In total, how many times have you taken the theory test for car drivers?

_______________ times

(b) When did you take your FIRST theory test for car drivers?

_______________ month _______________ year

C5 Did the FIRST theory test you took include a Hazard Perception test using film clips?

Yes ☐

No ☐

PLEASE ANSWER QUESTIONS C6 TO C9 ABOUT THE FIRST THEORY TEST THAT YOU TOOK

C6 Had you driven at all before taking your first theory test?

Yes ☐

No ☐ ➡ ***Go to C8***

C7 How many hours driving experience had you had in total when you took your first theory test? Please include both professional lessons and driving practice with friends or relations.

_______________ **hours**

C8 In your opinion, how ready to apply for your practical driving test were you when you took your theory test?

(Tick ONE box only)

I was ready to apply for my practical driving test ☐

I was nearly ready to apply for my practical driving test ☐

I was not at all ready to apply for my practical driving test ☐

For office use only

C9 Which of the following did your professional driving instructor do…

(Tick ALL that apply)

	(i) when preparing for your theory test	(ii) between your theory test and practical test
Taught or tested you on knowledge of road signs and markings	☐ 1	☐ 1
Taught or tested you on other driving- related knowledge	☐ 2	☐ 2
Taught or tested you on hazard awareness skills	☐ 3	☐ 3
Recommended particular driving-related books or materials	☐ 4	☐ 4
Went through example theory test questions	☐ 5	☐ 5
Did not have any lessons with a professional driving instructor	☐ 6	☐ 6
Other (***please specify***) ____________________	☐ 7	☐ 7
Nothing	☐ 8	☐ 8

C10 Which of the following did you use when preparing for your theory and practical driving tests?

(Tick ALL that apply)

	(i) Theory test: Multiple Choice part	(i) Theory test: Hazard Perception part (if included in your theory test)	(ii) Practical test
DSA book 'Official theory Test for Car Drivers'	☐ 1	☐ 1	☐ 1
DSA book 'The Driving Manual'/'Driving-the essential skills'	☐ 2	☐ 2	☐ 2
DSA 'Roadsense'	☐ 3	☐ 3	☐ 3
Highway code	☐ 4	☐ 4	☐ 4
'Know your traffic signs' book	☐ 5	☐ 5	☐ 5
Other books or sets of example questions	☐ 6	☐ 6	☐ 6
An interactive video, CD or DVD based at a driving school	☐ 7	☐ 7	☐ 7
Other driving related video(s), CD(s) or DVD(s)	☐ 8	☐ 8	☐ 8
A website	☐ 9	☐ 9	☐ 9
Other (***please specify***) ____________________	☐ 10	☐ 10	☐ 10
I did not use any material to prepare for the test	☐ 11	☐ 11	☐ 11

For office use only

SECTION D: ATTITUDES TOWARDS DRIVING

D1 The following questions ask about your confidence in your own driving ability.

(*Please tick ONE box per line*)

	Very confident	Fairly confident	Not very confident	Not at all confident
**(a)** How confident were you in your driving ability when you were learning to drive?	☐	☐	☐	☐
**(b)** In the week before you took your practical driving test, how confident were you that you would pass?	☐	☐	☐	☐
**(c)** In the week before you took your practical driving test, how confident were you that you were a good enough driver to drive on the road unsupervised?	☐	☐	☐	☐
**(d)** How confident are you in your driving ability now?	☐	☐	☐	☐

D2 How much do you think you need to improve your ability on each of the following driving skills?

(Tick ONE box per line)

	No improvement needed	Some improvement needed	A lot of improvement needed
**(a)** Use of car controls	☐	☐	☐
**(b)** Pulling out of junctions	☐	☐	☐
**(c)** Reversing	☐	☐	☐
**(d)** Parking	☐	☐	☐
**(e)** Judging the speed of other traffic	☐	☐	☐
**(f)** Judging what other drivers are going to do	☐	☐	☐
**(g)** Spotting hazards	☐	☐	☐
**(h)** Driving in heavy traffic	☐	☐	☐
**(i)** Driving in the dark	☐	☐	☐
**(j)** Overtaking	☐	☐	☐
**(k)** Using roundabouts	☐	☐	☐
**(l)** Joining with moving traffic on a motorway or fast dual carriageway	☐	☐	☐
**(m)** Changing lanes on a motorway or fast dual carriageway	☐	☐	☐
**(n)** Driving on high speed roads	☐	☐	☐
**(o)** Driving on country roads	☐	☐	☐

For office use only

D3 How much do you agree or disagree with each of the following statements?

(*Tick ONE box on each line*)

		Strongly agree (1)	Agree (2)	Neither agree nor disagree (3)	Disagree (4)	Strongly disagree (5)
**(a)**	Decreasing the motorway speed limit is a good idea					
**(b)**	Even at night-time on quiet roads it is important to keep within the speed limit					
**(c)**	Drivers who cause accidents by reckless driving should be banned from driving for life					
**(d)**	People should drive slower than the speed limit when it is raining					
**(e)**	Cars should never overtake on the inside lane even if a slow driver is blocking the outside lane					
**(f)**	In towns where there are a lot of pedestrians the speed limit should be 20mph					
**(g)**	Penalties for speeding should be more severe					
**(h)**	Increasing the motorway speed limit is a good idea					

D4 We would like to know what kind of driver you think you are by putting a tick somewhere on each of the lines below.

At either end of each line there is a word that describes a way of driving, and these words are opposites. Put your tick nearer to the word that best describes your driving. The closer your tick is to the word, the more you agree with this description of the way you drive.

	(1)	(2)	(3)	(4)	(5)	(6)	(7)	
Attentive								Inattentive
Careful								Careless
Decisive								Indecisive
Experienced								Inexperienced
Irritable								Placid
Nervous								Confident
Patient								Impatient
Responsible								Irresponsible
Safe								Risky
Selfish								Considerate
Slow								Fast
Tolerant								Intolerant

SECTION E: CONTACT DETAILS

For the success of this important research, we would like to get in touch with you again to see how your driving is going.

Please fill in as many of the contact details below as possible.

All information will be treated in the STRICTEST CONFIDENCE and will not be used for any purpose other than to get in touch with you in relation to this research.

E1 Home phone number: ______________________________

E2 Work phone number: ______________________________
(*if applicable*)

E3 Mobile phone number: ______________________________

E4 Email address: ______________________________

If the address that this questionnaire was sent to is incorrect, or if there is a better address to help us contact you in the future, please provide the new address in the space below.

E5 New address: ______________________________

____

____

Postcode: ______________________________

E6 If you have any further comments to make about this survey and the issues raised, please write in the space provided:

E7 Please now return this questionnaire in the pre-paid envelope to:
NFER, The Mere, Upton Park, Slough, Berkshire, SL1 2DQ

Please return this questionnaire even if you have only answered the first few questions, as this information is equally important to us and will allow us to remove your name from our mailing list for future questionnaires.

Thank you very much for your help.

LEARNING TO DRIVE QUESTIONNAIRE

Please complete this questionnaire by ticking the appropriate boxes and filling in the spaces as required. Several questions ask for dates or lengths of time etc, if you are unable to give an exact answer to any of these questions please give as good an estimate as you can. It will only take a few minutes to complete this questionnaire and any information you provide will be treated in the **strictest confidence** and will be used for statistical purposes only.

SECTION A: YOUR PRACTICAL DRIVING TEST

A1 Did you take your practical driving test in the week beginning Monday 4th August 2003?

Yes ☐ ➡ ***Go to A2***

No ☐ ➡ ***Go to E7***

A2 When you took your practical driving test in the week beginning Monday 4th August 2003 what type of licence did you hold?

(Tick ONE box only)

Provisional licence to drive a car ☐ ➡ ***Go to A3***

Full licence for another class of vehicle ☐ ➡ ***Go to E7***

A3 Had you previously held a FULL (GB or non- GB) car driving licence before taking your practical driving test in the week beginning Monday 4th August 2003?

(Tick ONE box only)

NO ☐ ➡ ***Go to A4***

YES – Full GB licence ☐ ➡ ***Go to E7***

YES – Full non- GB licence ☐ ➡ ***Go to E7***

For office use only

A4 How old were you when you got your first provisional car licence?

________________ years ________________ months

A5 Before you started learning to drive a car did you regularly ride a motorcycle, moped, or scooter?

Yes ☐

No ☐

A6 Did you take your practical driving test in the week beginning Monday 4th August 2003 in:
(Tick ONE box only)

A driving instructor's car? ☐ 1

A car owned by a friend/relation? ☐ 2

Your own car? ☐ 3

Another car? ☐ 4

A7 Was this practical driving test your:
(Tick ONE box only)

First? ☐ 1 ➡ ***Go to A9***

Second? ☐ 2

Third? ☐ 3

Fourth? ☐ 4

More than fourth? ☐ 5

A8 When did you fail the practical driving test before the one in the week beginning Monday 4th August 2003?

Day________________ Month ________________________________ Year ________________

A9 Did you pass the practical driving test that you took in the week beginning Monday 4th August 2003?

Yes ☐ ➡ ***Go to B1***

No ☐

A10 Did you agree with the Examiner's decision to fail you?

Yes ☐

No ☐

For office use only

A11 Why do you think you failed on this occasion? *(Tick ALL that apply)*

You were just 'unlucky' on the day	☐	1
Difficult weather conditions on the day of the test	☐	2
You were not ready for the test	☐	3
Nerves affected your performance	☐	4
Another road user was to blame	☐	5
Your driving was not good enough on the day	☐	6
You could not cope with something unexpected that happened	☐	7
Other (please specify)	☐	8

____

A12 Since failing the practical driving test in the week beginning Monday 4th August 2003, have you applied for another test?

Yes ☐

No ☐

A13 Before your next test do you think that you will... (*Tick ONE box only*)

Have professional driving lessons MORE OFTEN?	☐	1
Have professional driving lessons AS OFTEN AS BEFORE?	☐	2
Have professional driving lessons LESS OFTEN?	☐	3
START having professional driving lessons?	☐	4
NOT HAVE ANY professional driving lessons?	☐	5

A14 Before your next test do you think that you will... (*Tick ONE box only*)

Have practice driving sessions with friends or relations MORE OFTEN?	☐	1
Have practice driving sessions with friends or relations AS OFTEN AS BEFORE?	☐	2
Have practice driving sessions with friends or relations LESS OFTEN?	☐	3
START having practice driving sessions with friends or relations?	☐	4
NOT HAVE ANY practice driving sessions with friends or relations?	☐	5

For office use only

SECTION B: LEARNING TO DRIVE

B1 When did you begin to learn to drive a car?

Month ______________________ Year ____________

B2 Since you started learning to drive, was there any period of longer than 6 months during which you did no driving at all?

Yes ☐

No ☐ ➡ ***Go to B4***

B3 What was the total of all those periods during which you did no driving at all since you started learning to drive?

____________ years ____________ months

B4 How many miles IN TOTAL had you driven a car before you took your practical driving test in the week beginning Monday 4th August 2003?
(If you are not sure, please give your best estimate to the nearest 100 miles)

____________ miles

B5 Please estimate how your total driving time was split. ***(Tick ONE box only)***

All in a DRIVING INSTRUCTOR'S car	Almost all in a DRIVING INSTRUCTOR'S car	More than half in a DRIVING INSTRUCTOR'S car	About half and half	More than half in OTHER car(s)	Almost all in OTHER car(s)	All in OTHER car(s)
(1)	(2)	(3)	(4)	(5)	(6)	(7)
☐	☐	☐	☐	☐	☐	☐

B6 How many hours of lessons from a professional driving instructor have you had:

(a) IN TOTAL since you first started learning to drive?

____________ hours **None** ☐ ➡ ***If none, please go to B11***

(b) Between the practical driving test that you took in the week beginning Monday 28th April 2003 and your previous test?

____________ hours No previous practical driving test ☐

B7 How many different professional driving instructors have you ever had lessons from?

____________ (Please write number of instructors)

For office use only

B8 (a) Was your most recent professional driving instructor a DSA Approved Driving Instructor (ADI)?

Yes ☐
No ☐
Don't know ☐

(b) What grade of instructor is he or she? ***(Tick ONE box only)***

Grade 6 ADI ☐ 1
Grade 5 ADI ☐ 2
Grade 4 ADI ☐ 3
Trainee ADI ☐ 4
Don't know ☐ 5

(c) What was your main reason for choosing this instructor/driving school?

(Tick ONE box only)

Good reputation ☐ 1
Personal recommendation ☐ 2
You already knew the person ☐ 3
Cost of lessons ☐ 4
Local advertising ☐ 5
Large company/organisation ☐ 6
You saw them in the phone book ☐ 7
Highly qualified instructors ☐ 8
Other (*please specify*) ☐ 9

____

B9 Thinking about ALL the professional driving lessons that you took from when you started driving up to the time of your driving test in the week beginning Monday 4th August 2003, how much have you driven...

(Tick ONE box per line)

		Never	Less than 2 hours	2-4 hours	More than 4 hours
**(a)**	In a busy town centre	☐	☐	☐	☐
**(b)**	On country roads	☐	☐	☐	☐
**(c)**	On fast dual carriageways	☐	☐	☐	☐
**(d)**	In the dark	☐	☐	☐	☐
**(e)**	In the rain	☐	☐	☐	☐
**(f)**	On snow or ice	☐	☐	☐	☐

For office use only

B10 When you were preparing for the practical driving test you took in the week beginning Monday 4th August 2003, how did you spread out your professional lessons?

(*Tick ONE box only*)

Regularly up to the practical driving test just taken	☐	1
Most at the beginning with a few near the test	☐	2
Most near the test with a few at the beginning	☐	3
Some at the beginning and some near the test with a few in between	☐	4
Few at the beginning and few near the test with most in between	☐	5
Other (*please specify*)	☐	6

____

B11 How many hours did you spend practising your driving with friends or relations?

(a) None ☐ ➡ ***If none, please go to B14***

(b) IN TOTAL since you first started learning to drive?

____________ hours

(c) Between the practical driving test that you took in the week beginning Monday 28th April 2003 and your previous car driving test?

____________ hours No previous practical driving test ☐

B12 Thinking about ALL the practice driving with friends and relatives that you did from when you started driving up to the time of your driving test in the week beginning Monday 4th August 2003, how much have you driven...

(Tick ONE box per line)

	Never	Less than 2 hours	2-4 hours	More than 4 hours
**(a)** In a busy town centre	☐	☐	☐	☐
**(b)** On country roads	☐	☐	☐	☐
**(c)** On fast dual carriageways	☐	☐	☐	☐
**(d)** In the dark	☐	☐	☐	☐
**(e)** In the rain	☐	☐	☐	☐
**(f)** On snow or ice	☐	☐	☐	☐

For office use only

B13 When you were preparing for the practical driving test you took in the week beginning Monday 4th August 2003, how did you spread out your sessions of driving practice with friends and relations?

(***Tick ONE box only***)

Regularly up to the practical driving test just taken	☐	1
Most at the beginning with a few near the test	☐	2
Most near the test with a few at the beginning	☐	3
Some at the beginning and some near the test with a few in between	☐	4
Few at the beginning and few near the test with most in between	☐	5
Other (*please specify*)	☐	6

____

B14 When you were preparing for the practical driving test you took in the week beginning Monday 4th August 2003:

		**Yes**	**No**
**(a)**	Was it hard to find a friend or relation to take you out for driving practice?	☐	☐
**(b)**	Were you able to have as much driving practice with friends or relations as you wanted?	☐	☐
**(c)**	Was it difficult to find time to fit in driving practice with friends or relations?	☐	☐
**(d)**	Did the cost of professional lessons affect the number you took?	☐	☐
**(e)**	Do you feel that you had professional lessons often enough?	☐	☐
**(f)**	Was it difficult to find time to fit in professional lessons?	☐	☐
**(g)**	When you applied for the test did you think you had a good chance of passing it?	☐	☐
**(h)**	When you took the test did you think you had a good chance of passing it?	☐	☐
**(i)**	Were you keen to take your test as soon as possible?	☐	☐
**(j)**	Did you find learning to drive easy?	☐	☐

For office use only

SECTION C: YOUR THEORY TEST

C1 When did you pass your theory test for car drivers?

______________ month ______________ year

C2 Did the theory test you passed include a Hazard Perception test using film clips?

Yes ☐

No ☐

C3 Was this the first time you had taken the theory test for car drivers?

Yes ☐ ➡ ***Go to C6***

No ☐

C4 (a) In total, how many times have you taken the theory test for car drivers?

______________ times

(b) When did you take your FIRST theory test for car drivers?

______________ month ______________ year

C5 Did the FIRST theory test you took include a Hazard Perception test using film clips?

Yes ☐

No ☐

PLEASE ANSWER QUESTIONS C6 TO C9 ABOUT THE <u>FIRST</u> THEORY TEST THAT YOU TOOK

C6 Had you driven at all before taking your first theory test?

Yes ☐

No ☐ ➡ ***Go to C8***

C7 How many hours driving experience had you had in total when you took your first theory test? Please include both professional lessons and driving practice with friends or relations.

______________ **hours**

C8 In your opinion, how ready to apply for your practical driving test were you when you took your theory test?

(Tick ONE box only)

I was ready to apply for my practical driving test ☐

I was nearly ready to apply for my practical driving test ☐

I was not at all ready to apply for my practical driving test ☐

For office use only

C9 Which of the following did your professional driving instructor do…

(Tick ALL that apply)

	(i) when preparing for your theory test	(ii) between your theory test and practical test
Taught or tested you on knowledge of road signs and markings	1	1
Taught or tested you on other driving- related knowledge	2	2
Taught or tested you on hazard awareness skills	3	3
Recommended particular driving-related books or materials	4	4
Went through example theory test questions	5	5
Did not have any lessons with a professional driving instructor	6	6
Other (*please specify*) ________________	7	7
Nothing	8	8

C10 Which of the following did you use when preparing for your theory and practical driving tests?

(Tick ALL that apply)

	(i) Theory test: Multiple Choice part	(i) Theory test: Hazard Perception part (if included in your theory test)	(ii) Practical test
DSA book 'Official theory Test for Car Drivers'	1	1	1
DSA book 'The Driving Manual'/'Driving-the essential skills'	2	2	2
DSA 'Roadsense'	3	3	3
Highway code	4	4	4
'Know your traffic signs' book	5	5	5
Other books or sets of example questions	6	6	6
An interactive video, CD or DVD based at a driving school	7	7	7
Other driving related video(s), CD(s) or DVD(s)	8	8	8
A website	9	9	9
Other (*please specify*) ________________	10	10	10
I did not use any material to prepare for the test	11	11	11

For office use only

SECTION D: ATTITUDES TOWARDS DRIVING

D1 The following questions ask about your confidence in your own driving ability.

(*Please tick ONE box per line*)

	Very confident	Fairly confident	Not very confident	Not at all confident
**(a)** How confident were you in your driving ability when you were learning to drive?	☐	☐	☐	☐
**(b)** In the week before you took your practical driving test, how confident were you that you would pass?	☐	☐	☐	☐
**(c)** In the week before you took your practical driving test, how confident were you that you were a good enough driver to drive on the road unsupervised?	☐	☐	☐	☐
**(d)** How confident are you in your driving ability now?	☐	☐	☐	☐

D2 How much do you think you need to improve your ability on each of the following driving skills?

(Tick ONE box per line)

	No improvement needed	Some improvement needed	A lot of improvement needed
**(a)** Use of car controls	☐	☐	☐
**(b)** Pulling out of junctions	☐	☐	☐
**(c)** Reversing	☐	☐	☐
**(d)** Parking	☐	☐	☐
**(e)** Judging the speed of other traffic	☐	☐	☐
**(f)** Judging what other drivers are going to do	☐	☐	☐
**(g)** Spotting hazards	☐	☐	☐
**(h)** Driving in heavy traffic	☐	☐	☐
**(i)** Driving in the dark	☐	☐	☐
**(j)** Overtaking	☐	☐	☐
**(k)** Using roundabouts	☐	☐	☐
**(l)** Joining with moving traffic on a motorway or fast dual carriageway	☐	☐	☐
**(m)** Changing lanes on a motorway or fast dual carriageway	☐	☐	☐
**(n)** Driving on high speed roads	☐	☐	☐
**(o)** Driving on country roads	☐	☐	☐

D3 How much do you agree or disagree with each of the following statements?

(*Tick ONE box on each line*)

	Strongly agree (1)	Agree (2)	Neither agree nor disagree (3)	Disagree (4)	Strongly disagree (5)
**(a)** Decreasing the motorway speed limit is a good idea					
**(b)** Even at night-time on quiet roads it is important to keep within the speed limit					
**(c)** Drivers who cause accidents by reckless driving should be banned from driving for life					
**(d)** People should drive slower than the speed limit when it is raining					
**(e)** Cars should never overtake on the inside lane even if a slow driver is blocking the outside lane					
**(f)** In towns where there are a lot of pedestrians the speed limit should be 20mph					
**(g)** Penalties for speeding should be more severe					
**(h)** Increasing the motorway speed limit is a good idea					

D4 We would like to know what kind of driver you think you are by putting a tick somewhere on each of the lines below.

At either end of each line there is a word that describes a way of driving, and these words are opposites. Put your tick nearer to the word that best describes your driving. The closer your tick is to the word, the more you agree with this description of the way you drive.

	(1)	(2)	(3)	(4)	(5)	(6)	(7)	
Attentive								Inattentive
Careful								Careless
Decisive								Indecisive
Experienced								Inexperienced
Irritable								Placid
Nervous								Confident
Patient								Impatient
Responsible								Irresponsible
Safe								Risky
Selfish								Considerate
Slow								Fast
Tolerant								Intolerant

SECTION E: CONTACT DETAILS

For the success of this important research, we would like to get in touch with you again to see how your driving is going.

Please fill in as many of the contact details below as possible.

All information will be treated in the STRICTEST CONFIDENCE and will not be used for any purpose other than to get in touch with you in relation to this research.

E1 Home phone number: ______________________________

E2 Work phone number: ______________________________
(*if applicable*)

E3 Mobile phone number: ______________________________

E4 Email address: ______________________________

If the address that this questionnaire was sent to is incorrect, or if there is a better address to help us contact you in the future, please provide the new address in the space below.

E5 New address: ______________________________

____

____

Postcode: ______________________________

E6 If you have any further comments to make about this survey and the issues raised, please write in the space provided:

E7 Please now return this questionnaire in the pre-paid envelope to:
NFER, The Mere, Upton Park, Slough, Berkshire, SL1 2DQ

Please return this questionnaire even if you have only answered the first few questions, as this information is equally important to us and will allow us to remove your name from our mailing list for future questionnaires.

Thank you very much for your help.

LEARNING TO DRIVE QUESTIONNAIRE

Please complete this questionnaire by ticking the appropriate boxes and filling in the spaces as required. Several questions ask for dates or lengths of time etc, if you are unable to give an exact answer to any of these questions please give as good an estimate as you can. It will only take a few minutes to complete this questionnaire and any information you provide will be treated in the **strictest confidence** and will be used for statistical purposes only.

SECTION A: YOUR PRACTICAL DRIVING TEST

A1 Did you take your practical driving test in the week beginning Monday 3rd November 2003?

Yes ☐ ➧ *Go to A2*

No ☐ ➧ *Go to E7*

A2 When you took your practical driving test in the week beginning Monday 3rd November 2003 what type of licence did you hold?

(Tick ONE box only)

Provisional licence to drive a car ☐ ➧ *Go to A3*

Full licence for another class of vehicle ☐ ➧ *Go to E7*

A3 Had you previously held a FULL (GB or non- GB) car driving licence before taking your practical driving test in the week beginning Monday 3rd November 2003?

(Tick ONE box only)

NO ☐ ➧ *Go to A4*

YES – Full GB licence ☐ ➧ *Go to E7*

YES – Full non- GB licence ☐ ➧ *Go to E7*

For office use only

A4 How old were you when you got your first provisional car licence?

_______________ years _______________ months

A5 Before you started learning to drive a car did you regularly ride a motorcycle, moped, or scooter?

Yes ☐

No ☐

A6 Did you take your practical driving test in the week beginning Monday 3rd November 2003 in:
(Tick ONE box only)

A driving instructor's car? ☐ 1

A car owned by a friend/relation? ☐ 2

Your own car? ☐ 3

Another car? ☐ 4

A7 Was this practical driving test your:
(Tick ONE box only)

First? ☐ 1 ➡ ***Go to A9***

Second? ☐ 2

Third? ☐ 3

Fourth? ☐ 4

More than fourth? ☐ 5

A8 When did you fail the practical driving test before the one in the week beginning Monday 3rd November 2003?

Day__________ Month ____________________ Year __________

A9 Did you pass the practical driving test that you took in the week beginning Monday 3rd November 2003?

Yes ☐ ➡ ***Go to B1***

No ☐

A10 Did you agree with the Examiner's decision to fail you?

Yes ☐

No ☐

For office use only

A11 Why do you think you failed on this occasion? ***(Tick ALL that apply)***

You were just 'unlucky' on the day	☐	1
Difficult weather conditions on the day of the test	☐	2
You were not ready for the test	☐	3
Nerves affected your performance	☐	4
Another road user was to blame	☐	5
Your driving was not good enough on the day	☐	6
You could not cope with something unexpected that happened	☐	7
Other (please specify)	☐	8

____

A12 Since failing the practical driving test in the week beginning Monday 3rd November 2003, have you applied for another test?

Yes ☐

No ☐

A13 Before your next test do you think that you will... **(*Tick ONE box only*)**

Have professional driving lessons MORE OFTEN?	☐	1
Have professional driving lessons AS OFTEN AS BEFORE?	☐	2
Have professional driving lessons LESS OFTEN?	☐	3
START having professional driving lessons?	☐	4
NOT HAVE ANY professional driving lessons?	☐	5

A14 Before your next test do you think that you will... **(*Tick ONE box only*)**

Have practice driving sessions with friends or relations MORE OFTEN?	☐	1
Have practice driving sessions with friends or relations AS OFTEN AS BEFORE?	☐	2
Have practice driving sessions with friends or relations LESS OFTEN?	☐	3
START having practice driving sessions with friends or relations?	☐	4
NOT HAVE ANY practice driving sessions with friends or relations?	☐	5

For office use only

SECTION B: LEARNING TO DRIVE

B1 When did you begin to learn to drive a car?

Month ______________________ Year ____________

B2 Since you started learning to drive, was there any period of longer than 6 months during which you did no driving at all?

Yes ☐

No ☐ ➡ ***Go to B4***

B3 What was the total of all those periods during which you did no driving at all since you started learning to drive?

____________ years ____________ months

B4 How many miles IN TOTAL had you driven a car before you took your practical driving test in the week beginning Monday 3rd November 2003?

(If you are not sure, please give your best estimate to the nearest 100 miles)

____________ miles

B5 Please estimate how your total driving time was split. ***(Tick ONE box only)***

All in a DRIVING INSTRUCTOR'S car	Almost all in a DRIVING INSTRUCTOR'S car	More than half in a DRIVING INSTRUCTOR'S car	About half and half	More than half in OTHER car(s)	Almost all in OTHER car(s)	All in OTHER car(s)
(1)	(2)	(3)	(4)	(5)	(6)	(7)
☐	☐	☐	☐	☐	☐	☐

B6 How many hours of lessons from a professional driving instructor have you had:

(a) IN TOTAL since you first started learning to drive?

____________ hours **None** ☐ ➡ ***If none, please go to B11***

(b) Between the practical driving test that you took in the week beginning Monday 3rd November 2003 and your previous test?

____________ hours No previous practical driving test ☐

B7 How many different professional driving instructors have you ever had lessons from?

____________ (Please write number of instructors)

For office use only

B8 (a) Was your most recent professional driving instructor a DSA Approved Driving Instructor (ADI)?

- Yes ☐
- No ☐
- Don't know ☐

(b) What grade of instructor is he or she? ***(Tick ONE box only)***

- Grade 6 ADI ☐ 1
- Grade 5 ADI ☐ 2
- Grade 4 ADI ☐ 3
- Trainee ADI ☐ 4
- Don't know ☐ 5

(c) What was your main reason for choosing this instructor/driving school?

(Tick ONE box only)

- Good reputation ☐ 1
- Personal recommendation ☐ 2
- You already knew the person ☐ 3
- Cost of lessons ☐ 4
- Local advertising ☐ 5
- Large company/organisation ☐ 6
- You saw them in the phone book ☐ 7
- Highly qualified instructors ☐ 8
- Other (*please specify*) ☐ 9

____

B9 Thinking about ALL the professional driving lessons that you took from when you started driving up to the time of your driving test in the week beginning Monday 3rd November 2003, how much have you driven...

(Tick ONE box per line)

	Never	Less than 2 hours	2-4 hours	More than 4 hours
**(a)** In a busy town centre	☐	☐	☐	☐
**(b)** On country roads	☐	☐	☐	☐
**(c)** On fast dual carriageways	☐	☐	☐	☐
**(d)** In the dark	☐	☐	☐	☐
**(e)** In the rain	☐	☐	☐	☐
**(f)** On snow or ice	☐	☐	☐	☐

For office use only

B10 When you were preparing for the practical driving test you took in the week beginning Monday 3rd November 2003, how did you spread out your professional lessons?
(Tick ONE box only)

Regularly up to the practical driving test just taken	☐	1
Most at the beginning with a few near the test	☐	2
Most near the test with a few at the beginning	☐	3
Some at the beginning and some near the test with a few in between	☐	4
Few at the beginning and few near the test with most in between	☐	5
Other (*please specify*)	☐	6

____

B11 How many hours did you spend practising your driving with friends or relations?

(a) None ☐ ➡ ***If none, please go to B14***

(b) IN TOTAL since you first started learning to drive?

______________ hours

(c) Between the practical driving test that you took in the week beginning Monday 3rd November 2003 and your previous car driving test?

______________ hours   No previous practical driving test ☐

B12 Thinking about ALL the practice driving with friends and relatives that you did from when you started driving up to the time of your driving test in the week beginning Monday 3rd November 2003, how much have you driven...
(Tick ONE box per line)

		Never	Less than 2 hours	2-4 hours	More than 4 hours
**(a)**	In a busy town centre	☐	☐	☐	☐
**(b)**	On country roads	☐	☐	☐	☐
**(c)**	On fast dual carriageways	☐	☐	☐	☐
**(d)**	In the dark	☐	☐	☐	☐
**(e)**	In the rain	☐	☐	☐	☐
**(f)**	On snow or ice	☐	☐	☐	☐

For office use only

B13 When you were preparing for the practical driving test you took in the week beginning Monday 3rd November 2003, how did you spread out your sessions of driving practice with friends and relations?

(*Tick ONE box only*)

Regularly up to the practical driving test just taken	☐	1
Most at the beginning with a few near the test	☐	2
Most near the test with a few at the beginning	☐	3
Some at the beginning and some near the test with a few in between	☐	4
Few at the beginning and few near the test with most in between	☐	5
Other (*please specify*)	☐	6

____

B14 When you were preparing for the practical driving test you took in the week beginning Monday 3rd November 2003:

		Yes	No
**(a)**	Was it hard to find a friend or relation to take you out for driving practice?	☐	☐
**(b)**	Were you able to have as much driving practice with friends or relations as you wanted?	☐	☐
**(c)**	Was it difficult to find time to fit in driving practice with friends or relations?	☐	☐
**(d)**	Did the cost of professional lessons affect the number you took?	☐	☐
**(e)**	Do you feel that you had professional lessons often enough?	☐	☐
**(f)**	Was it difficult to find time to fit in professional lessons?	☐	☐
**(g)**	When you applied for the test did you think you had a good chance of passing it?	☐	☐
**(h)**	When you took the test did you think you had a good chance of passing it?	☐	☐
**(i)**	Were you keen to take your test as soon as possible?	☐	☐
**(j)**	Did you find learning to drive easy?	☐	☐

For office use only

SECTION C: YOUR THEORY TEST

C1 When did you pass your theory test for car drivers?

_______________ month _______________ year

C2 Did the theory test you passed include a Hazard Perception test using film clips?

Yes ☐

No ☐

C3 Was this the first time you had taken the theory test for car drivers?

Yes ☐ ➡ ***Go to C6***

No ☐

C4 (a) In total, how many times have you taken the theory test for car drivers?

_______________ times

(b) When did you take your FIRST theory test for car drivers?

_______________ month _______________ year

C5 Did the FIRST theory test you took include a Hazard Perception test using film clips?

Yes ☐

No ☐

PLEASE ANSWER QUESTIONS C6 TO C9 ABOUT THE FIRST THEORY TEST THAT YOU TOOK

C6 Had you driven at all before taking your first theory test?

Yes ☐

No ☐ ➡ ***Go to C8***

C7 How many hours driving experience had you had in total when you took your first theory test? Please include both professional lessons and driving practice with friends or relations.

_______________ **hours**

C8 In your opinion, how ready to apply for your practical driving test were you when you took your theory test?

(Tick ONE box only)

I was ready to apply for my practical driving test ☐

I was nearly ready to apply for my practical driving test ☐

I was not at all ready to apply for my practical driving test ☐

For office use only

C9 Which of the following did your professional driving instructor do…

(Tick ALL that apply)

	(i) when preparing for your theory test	(ii) between your theory test and practical test
Taught or tested you on knowledge of road signs and markings	1	1
Taught or tested you on other driving- related knowledge	2	2
Taught or tested you on hazard awareness skills	3	3
Recommended particular driving-related books or materials	4	4
Went through example theory test questions	5	5
Did not have any lessons with a professional driving instructor	6	6
Other (*please specify*) ______________________	7	7
Nothing	8	8

C10 Which of the following did you use when preparing for your theory and practical driving tests?

(Tick ALL that apply)

	(i) Theory test: Multiple Choice part	(i) Theory test: Hazard Perception part (if included in your theory test)	(ii) Practical test
DSA book 'Official theory Test for Car Drivers'	1	1	1
DSA book 'The Driving Manual'/'Driving-the essential skills'	2	2	2
DSA 'Roadsense'	3	3	3
Highway code	4	4	4
'Know your traffic signs' book	5	5	5
Other books or sets of example questions	6	6	6
An interactive video, CD or DVD based at a driving school	7	7	7
Other driving related video(s), CD(s) or DVD(s)	8	8	8
A website	9	9	9
Other (*please specify*) ______________________	10	10	10
I did not use any material to prepare for the test	11	11	11

For office use only

SECTION D: ATTITUDES TOWARDS DRIVING

D1 The following questions ask about your confidence in your own driving ability.

(*Please tick ONE box per line*)

	Very confident	Fairly confident	Not very confident	Not at all confident
**(a)** How confident were you in your driving ability when you were learning to drive?	☐	☐	☐	☐
**(b)** In the week before you took your practical driving test, how confident were you that you would pass?	☐	☐	☐	☐
**(c)** In the week before you took your practical driving test, how confident were you that you were a good enough driver to drive on the road unsupervised?	☐	☐	☐	☐
**(d)** How confident are you in your driving ability now?	☐	☐	☐	☐

D2 How much do you think you need to improve your ability on each of the following driving skills?

(Tick ONE box per line)

	No improvement needed	Some improvement needed	A lot of improvement needed
**(a)** Use of car controls	☐	☐	☐
**(b)** Pulling out of junctions	☐	☐	☐
**(c)** Reversing	☐	☐	☐
**(d)** Parking	☐	☐	☐
**(e)** Judging the speed of other traffic	☐	☐	☐
**(f)** Judging what other drivers are going to do	☐	☐	☐
**(g)** Spotting hazards	☐	☐	☐
**(h)** Driving in heavy traffic	☐	☐	☐
**(i)** Driving in the dark	☐	☐	☐
**(j)** Overtaking	☐	☐	☐
**(k)** Using roundabouts	☐	☐	☐
**(l)** Joining with moving traffic on a motorway or fast dual carriageway	☐	☐	☐
**(m)** Changing lanes on a motorway or fast dual carriageway	☐	☐	☐
**(n)** Driving on high speed roads	☐	☐	☐
**(o)** Driving on country roads	☐	☐	☐

D3 How much do you agree or disagree with each of the following statements?

(*Tick ONE box on each line*)

		Strongly agree (1)	Agree (2)	Neither agree nor disagree (3)	Disagree (4)	Strongly disagree (5)
**(a)**	Decreasing the motorway speed limit is a good idea					
**(b)**	Even at night-time on quiet roads it is important to keep within the speed limit					
**(c)**	Drivers who cause accidents by reckless driving should be banned from driving for life					
**(d)**	People should drive slower than the speed limit when it is raining					
**(e)**	Cars should never overtake on the inside lane even if a slow driver is blocking the outside lane					
**(f)**	In towns where there are a lot of pedestrians the speed limit should be 20mph					
**(g)**	Penalties for speeding should be more severe					
**(h)**	Increasing the motorway speed limit is a good idea					

D4 We would like to know what kind of driver you think you are by putting a tick somewhere on each of the lines below.

At either end of each line there is a word that describes a way of driving, and these words are opposites. Put your tick nearer to the word that best describes your driving. The closer your tick is to the word, the more you agree with this description of the way you drive.

	(1)	(2)	(3)	(4)	(5)	(6)	(7)	
Attentive								Inattentive
Careful								Careless
Decisive								Indecisive
Experienced								Inexperienced
Irritable								Placid
Nervous								Confident
Patient								Impatient
Responsible								Irresponsible
Safe								Risky
Selfish								Considerate
Slow								Fast
Tolerant								Intolerant

For office use only

SECTION E: CONTACT DETAILS

For the success of this important research, we would like to get in touch with you again to see how your driving is going.

Please fill in as many of the contact details below as possible.

All information will be treated in the STRICTEST CONFIDENCE and will not be used for any purpose other than to get in touch with you in relation to this research.

E1 Home phone number: ______________________________

E2 Work phone number: ______________________________
(*if applicable*)

E3 Mobile phone number: ______________________________

E4 Email address: ______________________________

If the address that this questionnaire was sent to is incorrect, or if there is a better address to help us contact you in the future, please provide the new address in the space below.

E5 New address: ______________________________

____

____

____

Postcode: ______________________________

E6 If you have any further comments to make about this survey and the issues raised, please write in the space provided:

E7 Please now return this questionnaire in the pre-paid envelope to:
NFER, The Mere, Upton Park, Slough, Berkshire, SL1 2DQ

Please return this questionnaire even if you have only answered the first few questions, as this information is equally important to us and will allow us to remove your name from our mailing list for future questionnaires.

Thank you very much for your help.

LEARNING TO DRIVE QUESTIONNAIRE

Please complete this questionnaire by ticking the appropriate boxes and filling in the spaces as required. Several questions ask for dates or lengths of time etc, if you are unable to give an exact answer to any of these questions please give as good an estimate as you can. It will only take a few minutes to complete this questionnaire and any information you provide will be treated in the **strictest confidence** and will be used for statistical purposes only.

SECTION A: YOUR PRACTICAL DRIVING TEST

A1 Did you take your practical driving test in the week beginning Monday 1st November 2004?

Yes ☐ ➡ ***Go to A2***

No ☐ ➡ ***Go to E7***

A2 When you took your practical driving test in the week beginning Monday 1st November 2004 what type of licence did you hold?

(Tick ONE box only)

Provisional licence to drive a car ☐ ➡ ***Go to A3***

Full licence for another class of vehicle ☐ ➡ ***Go to E7***

A3 Had you previously held a FULL (GB or non- GB) car driving licence before taking your practical driving test in the week beginning Monday 1st November 2004?

(Tick ONE box only)

NO ☐ ➡ ***Go to A4***

YES – Full GB licence ☐ ➡ ***Go to E7***

YES – Full non- GB licence ☐ ➡ ***Go to E7***

For office use only

A4 How old were you when you got your first provisional car licence?

_______________ years _______________ months

A5 Before you started learning to drive a car did you regularly ride a motorcycle, moped, or scooter?

Yes ☐

No ☐

A6 Did you take your practical driving test in the week beginning Monday 1st November 2004 in:
(Tick ONE box only)

A driving instructor's car? ☐ 1

A car owned by a friend/relation? ☐ 2

Your own car? ☐ 3

Another car? ☐ 4

A7 Was this practical driving test your:
(Tick ONE box only)

First? ☐ 1 ➡ ***Go to A9***

Second? ☐ 2

Third? ☐ 3

Fourth? ☐ 4

More than fourth? ☐ 5

A8 When did you fail the practical driving test before the one in the week beginning Monday 1st November 2004?

Day_______________ Month ______________________________ Year _______________

A9 Did you pass the practical driving test that you took in the week beginning Monday 1st November 2004?

Yes ☐ ➡ ***Go to B1***

No ☐

A10 Did you agree with the Examiner's decision to fail you?

Yes ☐

No ☐

For office use only

A11 Why do you think you failed on this occasion? ***(Tick ALL that apply)***

You were just 'unlucky' on the day	☐	1
Difficult weather conditions on the day of the test	☐	2
You were not ready for the test	☐	3
Nerves affected your performance	☐	4
Another road user was to blame	☐	5
Your driving was not good enough on the day	☐	6
You could not cope with something unexpected that happened	☐	7
Other (please specify)	☐	8

____

A12 Since failing the practical driving test in the week beginning Monday 1st November 2004, have you applied for another test?

Yes ☐

No ☐

A13 Before your next test do you think that you will... **(*Tick ONE box only*)**

Have professional driving lessons MORE OFTEN?	☐	1
Have professional driving lessons AS OFTEN AS BEFORE?	☐	2
Have professional driving lessons LESS OFTEN?	☐	3
START having professional driving lessons?	☐	4
NOT HAVE ANY professional driving lessons?	☐	5

A14 Before your next test do you think that you will... **(*Tick ONE box only*)**

Have practice driving sessions with friends or relations MORE OFTEN?	☐	1
Have practice driving sessions with friends or relations AS OFTEN AS BEFORE?	☐	2
Have practice driving sessions with friends or relations LESS OFTEN?	☐	3
START having practice driving sessions with friends or relations?	☐	4
NOT HAVE ANY practice driving sessions with friends or relations?	☐	5

For office use only

SECTION B: LEARNING TO DRIVE

B1 When did you begin to learn to drive a car?

Month ______________________________ Year _______________

B2 Since you started learning to drive, was there any period of longer than 6 months during which you did no driving at all?

Yes ☐

No ☐ ➡ ***Go to B4***

B3 What was the total of all those periods during which you did no driving at all since you started learning to drive?

_______________ years _______________ months

B4 How many miles IN TOTAL had you driven a car before you took your practical driving test in the week beginning Monday 1st November 2004?

(If you are not sure, please give your best estimate to the nearest 100 miles)

_______________ miles

B5 Please estimate how your total driving time was split. ***(Tick ONE box only)***

All in a DRIVING INSTRUCTOR'S car	Almost all in a DRIVING INSTRUCTOR'S car	More than half in a DRIVING INSTRUCTOR'S car	About half and half	More than half in OTHER car(s)	Almost all in OTHER car(s)	All in OTHER car(s)
(1)	(2)	(3)	(4)	(5)	(6)	(7)
☐	☐	☐	☐	☐	☐	☐

B6 How many hours of lessons from a professional driving instructor have you had:

(a) IN TOTAL since you first started learning to drive?

_______________ hours **None** ☐ ➡ ***If none, please go to B11***

(b) Between the practical driving test that you took in the week beginning Monday 1st November 2004 and your previous test?

_______________ hours No previous practical driving test ☐

B7 How many different professional driving instructors have you ever had lessons from?

_______________ (Please write number of instructors)

For office use only

B8 What was your main reason for choosing your most recent instructor/driving school?

(Tick ONE box only)

Good reputation	☐	1
Personal recommendation	☐	2
You already knew the person	☐	3
Cost of lessons	☐	4
Local advertising	☐	5
Large company/organisation	☐	6
You saw them in the phone book	☐	7
Highly qualified instructors	☐	8
Other (*please specify*)	☐	9

____

B9 Thinking about ALL the professional driving lessons that you took from when you started driving up to the time of your driving test in the week beginning Monday 1st November 2004, how much have you driven...

(Tick ONE box per line)

		Never	Less than 2 hours	2-4 hours	More than 4 hours
**(a)**	In a busy town centre	☐	☐	☐	☐
**(b)**	On country roads	☐	☐	☐	☐
**(c)**	On fast dual carriageways	☐	☐	☐	☐
**(d)**	In the dark	☐	☐	☐	☐
**(e)**	In the rain	☐	☐	☐	☐
**(f)**	On snow or ice	☐	☐	☐	☐

B10 When you were preparing for the practical driving test you took in the week beginning Monday 1st November 2004, how did you spread out your professional lessons?

(*Tick ONE box only*)

Regularly up to the practical driving test just taken	☐	1
Most at the beginning with a few near the test	☐	2
Most near the test with a few at the beginning	☐	3
Some at the beginning and some near the test with a few in between	☐	4
Few at the beginning and few near the test with most in between	☐	5
Other (*please specify*)	☐	6

____

For office use only

B11 How many hours did you spend practising your driving with friends or relations?

(a) **None** ☐ ➡ ***If none, please go to B14***

(b) **IN TOTAL since you first started learning to drive?**

_______________ hours

(c) **Between the practical driving test that you took in the week beginning Monday 1st November 2004 and your previous car driving test?**

_______________ hours   No previous practical driving test ☐

B12 Thinking about ALL the practice driving with friends and relatives that you did from when you started driving up to the time of your driving test in the week beginning Monday 1st November 2004, how much have you driven...

(Tick ONE box per line)

		Never	Less than 2 hours	2-4 hours	More than 4 hours
**(a)**	In a busy town centre	☐	☐	☐	☐
**(b)**	On country roads	☐	☐	☐	☐
**(c)**	On fast dual carriageways	☐	☐	☐	☐
**(d)**	In the dark	☐	☐	☐	☐
**(e)**	In the rain	☐	☐	☐	☐
**(f)**	On snow or ice	☐	☐	☐	☐

B13 When you were preparing for the practical driving test you took in the week beginning Monday 1st November 2004, how did you spread out your sessions of driving practice with friends and relations?

(*Tick ONE box only*)

Regularly up to the practical driving test just taken	☐	1
Most at the beginning with a few near the test	☐	2
Most near the test with a few at the beginning	☐	3
Some at the beginning and some near the test with a few in between	☐	4
Few at the beginning and few near the test with most in between	☐	5
Other (*please specify*)	☐	6

____

For office use only

B14 When you were preparing for the practical driving test you took in the week beginning Monday 1st November 2004:

		Yes	No
**(a)**	Was it hard to find a friend or relation to take you out for driving practice?	☐	☐
**(b)**	Were you able to have as much driving practice with friends or relations as you wanted?	☐	☐
**(c)**	Was it difficult to find time to fit in driving practice with friends or relations?	☐	☐
**(d)**	Did the cost of professional lessons affect the number you took?	☐	☐
**(e)**	Do you feel that you had professional lessons often enough?	☐	☐
**(f)**	Was it difficult to find time to fit in professional lessons?	☐	☐
**(g)**	When you applied for the test did you think you had a good chance of passing it?	☐	☐
**(h)**	When you took the test did you think you had a good chance of passing it?	☐	☐
**(i)**	Were you keen to take your test as soon as possible?	☐	☐
**(j)**	Did you find learning to drive easy?	☐	☐

B15 When you were learning to drive did you:

(*Tick ALL that apply*)

Have a DSA Driver's Record that was sent from DVLA with your licence? ☐ 1

Have a DSA Driver's Record that you got from DSA? ☐ 2

Have a DSA Driver's Record from your driving instructor? ☐ 3

Have a different driver's record provided by your instructor? ☐ 4

(*please specify*) ______________________________

Not have any written record of your progress while learning to drive. ☐ 5 ➡ ***Go to C1***

B16 When you were learning to drive:

(*Tick ONE box per line*)

		Most of the time	Some of the time	Rarely	Never
**(a)**	How often did you use a DSA Driver's Record?	☐	☐	☐	☐
**(b)**	How often did you use a different driver's record?	☐	☐	☐	☐

For office use only

SECTION C: YOUR THEORY TEST

C1 When did you pass your theory test for car drivers?

________________ month ________ year

C2 Did the theory test you passed include a Hazard Perception test using film clips?

Yes ☐

No ☐

C3 Was this the first time you had taken the theory test for car drivers?

Yes ☐ ➡ ***Go to C6***

No ☐

C4 (a) In total, how many times have you taken the theory test for car drivers?

________ times

(b) When did you take your FIRST theory test for car drivers?

________________ month ________ year

C5 Did the FIRST theory test you took include a Hazard Perception test using film clips?

Yes ☐

No ☐

PLEASE ANSWER QUESTIONS C6 TO C9 ABOUT THE <u>FIRST</u> THEORY TEST THAT YOU TOOK

C6 Had you driven at all before taking your first theory test?

Yes ☐

No ☐ ➡ ***Go to C8***

C7 How many hours driving experience had you had in total when you took your first theory test? Please include both professional lessons and driving practice with friends or relations.

________ **hours**

C8 In your opinion, how ready to apply for your practical driving test were you when you took your theory test?

(Tick ONE box only)

I was ready to apply for my practical driving test ☐

I was nearly ready to apply for my practical driving test ☐

I was not at all ready to apply for my practical driving test ☐

For office use only

C9 Which of the following did your professional driving instructor do…

(Tick ALL that apply)

	(i) when preparing for your theory test	(ii) between your theory test and practical test
Taught or tested you on knowledge of road signs and markings	☐ 1	☐ 1
Taught or tested you on other driving- related knowledge	☐ 2	☐ 2
Taught or tested you on hazard awareness skills	☐ 3	☐ 3
Recommended particular driving-related books or materials	☐ 4	☐ 4
Went through example theory test questions	☐ 5	☐ 5
Did not have any lessons with a professional driving instructor	☐ 6	☐ 6
Other (*please specify*) ______________________	☐ 7	☐ 7
Nothing	☐ 8	☐ 8

C10 Which of the following did you use when preparing for your theory and practical driving tests?

(Tick ALL that apply)

	(i) Theory test: Multiple Choice part	(i) Theory test: Hazard Perception part (if included in your theory test)	(ii) Practical test
DSA book 'Official theory Test for Car Drivers'	☐ 1	☐ 1	☐ 1
DSA book 'The Driving Manual'/'Driving-the essential skills'	☐ 2	☐ 2	☐ 2
DSA 'Roadsense'	☐ 3	☐ 3	☐ 3
Highway code	☐ 4	☐ 4	☐ 4
'Know your traffic signs' book	☐ 5	☐ 5	☐ 5
Other books or sets of example questions	☐ 6	☐ 6	☐ 6
An interactive video, CD or DVD based at a driving school	☐ 7	☐ 7	☐ 7
Other driving related video(s), CD(s) or DVD(s)	☐ 8	☐ 8	☐ 8
A website	☐ 9	☐ 9	☐ 9
Other (*please specify*) ______________________	☐ 10	☐ 10	☐ 10
I did not use any material to prepare for the test	☐ 11	☐ 11	☐ 11

For office use only

SECTION D: ATTITUDES TOWARDS DRIVING

D1 The following questions ask about your confidence in your own driving ability.

(*Please tick ONE box per line*)

	Very confident	Fairly confident	Not very confident	Not at all confident
**(a)** How confident were you in your driving ability when you were learning to drive?	☐	☐	☐	☐
**(b)** In the week before you took your practical driving test, how confident were you that you would pass?	☐	☐	☐	☐
**(c)** In the week before you took your practical driving test, how confident were you that you were a good enough driver to drive on the road unsupervised?	☐	☐	☐	☐
**(d)** How confident are you in your driving ability now?	☐	☐	☐	☐

D2 How much do you think you need to improve your ability on each of the following driving skills?

(Tick ONE box per line)

	No improvement needed	Some improvement needed	A lot of improvement needed
**(a)** Use of car controls	☐	☐	☐
**(b)** Pulling out of junctions	☐	☐	☐
**(c)** Reversing	☐	☐	☐
**(d)** Parking	☐	☐	☐
**(e)** Judging the speed of other traffic	☐	☐	☐
**(f)** Judging what other drivers are going to do	☐	☐	☐
**(g)** Spotting hazards	☐	☐	☐
**(h)** Driving in heavy traffic	☐	☐	☐
**(i)** Driving in the dark	☐	☐	☐
**(j)** Overtaking	☐	☐	☐
**(k)** Using roundabouts	☐	☐	☐
**(l)** Joining with moving traffic on a motorway or fast dual carriageway	☐	☐	☐
**(m)** Changing lanes on a motorway or fast dual carriageway	☐	☐	☐
**(n)** Driving on high speed roads	☐	☐	☐
**(o)** Driving on country roads	☐	☐	☐

D3 How much do you agree or disagree with each of the following statements?

(Tick ONE box on each line)

	Strongly agree (1)	Agree (2)	Neither agree nor disagree (3)	Disagree (4)	Strongly disagree (5)
**(a)** Decreasing the motorway speed limit is a good idea					
**(b)** Even at night-time on quiet roads it is important to keep within the speed limit					
**(c)** Drivers who cause accidents by reckless driving should be banned from driving for life					
**(d)** People should drive slower than the speed limit when it is raining					
**(e)** Cars should never overtake on the inside lane even if a slow driver is blocking the outside lane					
**(f)** In towns where there are a lot of pedestrians the speed limit should be 20mph					
**(g)** Penalties for speeding should be more severe					
**(h)** Increasing the motorway speed limit is a good idea					

D4 We would like to know what kind of driver you think you are by putting a tick somewhere on each of the lines below.

At either end of each line there is a word that describes a way of driving, and these words are opposites. Put your tick nearer to the word that best describes your driving. The closer your tick is to the word, the more you agree with this description of the way you drive.

	(1)	(2)	(3)	(4)	(5)	(6)	(7)	
Attentive								Inattentive
Careful								Careless
Decisive								Indecisive
Experienced								Inexperienced
Irritable								Placid
Nervous								Confident
Patient								Impatient
Responsible								Irresponsible
Safe								Risky
Selfish								Considerate
Slow								Fast
Tolerant								Intolerant

For office use only

SECTION E: CONTACT DETAILS

For the success of this important research, we would like to get in touch with you again to see how your driving is going.

Please fill in as many of the contact details below as possible.

All information will be treated in the STRICTEST CONFIDENCE and will not be used for any purpose other than to get in touch with you in relation to this research.

E1 Home phone number: ______________________

E2 Work phone number: ______________________
(*if applicable*)

E3 Mobile phone number: ______________________

E4 Email address: ______________________

If the address that this questionnaire was sent to is incorrect, or if there is a better address to help us contact you in the future, please provide the new address in the space below.

E5 New address: ______________________

____

____

____

Postcode: ______________________

E6 If you have any further comments to make about this survey and the issues raised, please write in the space provided:

E7 Please now return this questionnaire in the pre-paid envelope to:
NFER, The Mere, Upton Park, Slough, Berkshire, SL1 2DQ

Please return this questionnaire even if you have only answered the first few questions, as this information is equally important to us and will allow us to remove your name from our mailing list for future questionnaires.

Thank you very much for your help.

Department for Transport DSA TRL

LEARNING TO DRIVE QUESTIONNAIRE

Please complete this questionnaire by ticking the appropriate boxes and filling in the spaces as required. Several questions ask for dates or lengths of time etc, if you are unable to give an exact answer to any of these questions please give as good an estimate as you can. It will only take a few minutes to complete this questionnaire and any information you provide will be treated in the **strictest confidence** and will be used for statistical purposes only.

SECTION A: YOUR PRACTICAL DRIVING TEST

A1 Did you take your practical driving test in the week beginning Monday 31st January 2005?

Yes ☐ ➡ ***Go to A2***

No ☐ ➡ ***Go to E7***

A2 When you took your practical driving test in the week beginning Monday 31st January 2005 what type of licence did you hold?

(Tick ONE box only)

Provisional licence to drive a car ☐ ➡ ***Go to A3***

Full licence for another class of vehicle ☐ ➡ ***Go to E7***

A3 Had you previously held a FULL (GB or non- GB) car driving licence before taking your practical driving test in the week beginning Monday 31st January 2005?

(Tick ONE box only)

NO ☐ ➡ ***Go to A4***

YES – Full GB licence ☐ ➡ ***Go to E7***

YES – Full non- GB licence ☐ ➡ ***Go to E7***

For office use only

A4 How old were you when you got your first provisional car licence?

_______________ years _______________ months

A5 Before you started learning to drive a car did you regularly ride a motorcycle, moped, or scooter?

Yes ☐

No ☐

A6 Did you take your practical driving test in the week beginning Monday 31st January 2005 in:
(Tick ONE box only)

A driving instructor's car? ☐ 1

A car owned by a friend/relation? ☐ 2

Your own car? ☐ 3

Another car? ☐ 4

A7 Was this practical driving test your:
(Tick ONE box only)

First? ☐ 1 ➡ ***Go to A9***

Second? ☐ 2

Third? ☐ 3

Fourth? ☐ 4

More than fourth? ☐ 5

A8 When did you fail the practical driving test before the one in the week beginning Monday 31st January 2005?

Day_______________ Month ______________________________ Year _______________

A9 Did you pass the practical driving test that you took in the week beginning Monday 31st January 2005?

Yes ☐ ➡ ***Go to B1***

No ☐

A10 Did you agree with the Examiner's decision to fail you?

Yes ☐

No ☐

For office use only

A11 Why do you think you failed on this occasion? ***(Tick ALL that apply)***

You were just 'unlucky' on the day	☐	1
Difficult weather conditions on the day of the test	☐	2
You were not ready for the test	☐	3
Nerves affected your performance	☐	4
Another road user was to blame	☐	5
Your driving was not good enough on the day	☐	6
You could not cope with something unexpected that happened	☐	7
Other (please specify)	☐	8

____

A12 Since failing the practical driving test in the week beginning Monday 31st January 2005, have you applied for another test?

Yes ☐

No ☐

A13 Before your next test do you think that you will... **(*Tick ONE box only*)**

Have professional driving lessons MORE OFTEN?	☐	1
Have professional driving lessons AS OFTEN AS BEFORE?	☐	2
Have professional driving lessons LESS OFTEN?	☐	3
START having professional driving lessons?	☐	4
NOT HAVE ANY professional driving lessons?	☐	5

A14 Before your next test do you think that you will... **(*Tick ONE box only*)**

Have practice driving sessions with friends or relations MORE OFTEN?	☐	1
Have practice driving sessions with friends or relations AS OFTEN AS BEFORE?	☐	2
Have practice driving sessions with friends or relations LESS OFTEN?	☐	3
START having practice driving sessions with friends or relations?	☐	4
NOT HAVE ANY practice driving sessions with friends or relations?	☐	5

For office use only

SECTION B: LEARNING TO DRIVE

B1 When did you begin to learn to drive a car?

Month ______________________________ Year _______________

B2 Since you started learning to drive, was there any period of longer than 6 months during which you did no driving at all?

Yes ☐

No ☐ ➡ ***Go to B4***

B3 What was the total of all those periods during which you did no driving at all since you started learning to drive?

_______________ years _______________ months

B4 How many miles IN TOTAL had you driven a car before you took your practical driving test in the week beginning Monday 31st January 2005?

(If you are not sure, please give your best estimate to the nearest 100 miles)

_______________ miles

B5 Please estimate how your total driving time was split. ***(Tick ONE box only)***

All in a DRIVING INSTRUCTOR'S car	Almost all in a DRIVING INSTRUCTOR'S car	More than half in a DRIVING INSTRUCTOR'S car	About half and half	More than half in OTHER car(s)	Almost all in OTHER car(s)	All in OTHER car(s)
(1)	(2)	(3)	(4)	(5)	(6)	(7)
☐	☐	☐	☐	☐	☐	☐

B6 How many hours of lessons from a professional driving instructor have you had:

(a) IN TOTAL since you first started learning to drive?

_______________ hours **None** ☐ ➡ ***If none, please go to B11***

(b) Between the practical driving test that you took in the week beginning Monday 31st January 2005 and your previous test?

_______________ hours No previous practical driving test ☐

B7 How many different professional driving instructors have you ever had lessons from?

_______________ (Please write number of instructors)

For office use only

B8 What was your main reason for choosing your most recent instructor/driving school?

(Tick ONE box only)

Good reputation	☐	1
Personal recommendation	☐	2
You already knew the person	☐	3
Cost of lessons	☐	4
Local advertising	☐	5
Large company/organisation	☐	6
You saw them in the phone book	☐	7
Highly qualified instructors	☐	8
Other (*please specify*)	☐	9

____

B9 Thinking about ALL the professional driving lessons that you took from when you started driving up to the time of your driving test in the week beginning Monday 31st January 2005, how much have you driven...

(Tick ONE box per line)

		Never	Less than 2 hours	2-4 hours	More than 4 hours
**(a)**	In a busy town centre	☐	☐	☐	☐
**(b)**	On country roads	☐	☐	☐	☐
**(c)**	On fast dual carriageways	☐	☐	☐	☐
**(d)**	In the dark	☐	☐	☐	☐
**(e)**	In the rain	☐	☐	☐	☐
**(f)**	On snow or ice	☐	☐	☐	☐

B10 When you were preparing for the practical driving test you took in the week beginning Monday 31st January 2005, how did you spread out your professional lessons?

(*Tick ONE box only*)

Regularly up to the practical driving test just taken	☐	1
Most at the beginning with a few near the test	☐	2
Most near the test with a few at the beginning	☐	3
Some at the beginning and some near the test with a few in between	☐	4
Few at the beginning and few near the test with most in between	☐	5
Other (*please specify*)	☐	6

____

B11 How many hours did you spend practising your driving with friends or relations?

(a) None ☐ ➨ ***If none, please go to B14***

(b) IN TOTAL since you first started learning to drive?

_______________ hours

(c) Between the practical driving test that you took in the week beginning Monday 31st January 2005 and your previous car driving test?

_______________ hours No previous practical driving test ☐

B12 Thinking about ALL the practice driving with friends and relatives that you did from when you started driving up to the time of your driving test in the week beginning Monday 31st January 2005, how much have you driven...

(Tick ONE box per line)

		Never	Less than 2 hours	2-4 hours	More than 4 hours
**(a)**	In a busy town centre	☐	☐	☐	☐
**(b)**	On country roads	☐	☐	☐	☐
**(c)**	On fast dual carriageways	☐	☐	☐	☐
**(d)**	In the dark	☐	☐	☐	☐
**(e)**	In the rain	☐	☐	☐	☐
**(f)**	On snow or ice	☐	☐	☐	☐

B13 When you were preparing for the practical driving test you took in the week beginning Monday 31st January 2005, how did you spread out your sessions of driving practice with friends and relations?

(*Tick ONE box only*)

Regularly up to the practical driving test just taken ☐ 1

Most at the beginning with a few near the test ☐ 2

Most near the test with a few at the beginning ☐ 3

Some at the beginning and some near the test with a few in between ☐ 4

Few at the beginning and few near the test with most in between ☐ 5

Other (*please specify*) ☐ 6

____

For office use only

B14 When you were preparing for the practical driving test you took in the week beginning Monday 31st January 2005:

		Yes	No
**(a)**	Was it hard to find a friend or relation to take you out for driving practice?	☐	☐
**(b)**	Were you able to have as much driving practice with friends or relations as you wanted?	☐	☐
**(c)**	Was it difficult to find time to fit in driving practice with friends or relations?	☐	☐
**(d)**	Did the cost of professional lessons affect the number you took?	☐	☐
**(e)**	Do you feel that you had professional lessons often enough?	☐	☐
**(f)**	Was it difficult to find time to fit in professional lessons?	☐	☐
**(g)**	When you applied for the test did you think you had a good chance of passing it?	☐	☐
**(h)**	When you took the test did you think you had a good chance of passing it?	☐	☐
**(i)**	Were you keen to take your test as soon as possible?	☐	☐
**(j)**	Did you find learning to drive easy?	☐	☐

B15 When you were learning to drive did you:

(*Tick ALL that apply*)

Have a DSA Driver's Record that was sent from DVLA with your licence? ☐ 1

Have a DSA Driver's Record that you got from DSA? ☐ 2

Have a DSA Driver's Record from your driving instructor? ☐ 3

Have a different driver's record provided by your instructor? ☐ 4

(*please specify*) ____________________

Not have any written record of your progress while learning to drive. ☐ 5 ➡ ***Go to C1***

B16 When you were learning to drive:

(*Tick ONE box per line*)

		Most of the time	Some of the time	Rarely	Never
**(a)**	How often did you use a DSA Driver's Record?	☐	☐	☐	☐
**(b)**	How often did you use a different driver's record?	☐	☐	☐	☐

For office use only

SECTION C: YOUR THEORY TEST

C1 When did you pass your theory test for car drivers?

____________________ month __________ year

C2 Did the theory test you passed include a Hazard Perception test using film clips?

Yes ☐

No ☐

C3 Was this the first time you had taken the theory test for car drivers?

Yes ☐ ➡ ***Go to C6***

No ☐

C4 (a) In total, how many times have you taken the theory test for car drivers?

__________ times

(b) When did you take your FIRST theory test for car drivers?

____________________ month __________ year

C5 Did the FIRST theory test you took include a Hazard Perception test using film clips?

Yes ☐

No ☐

PLEASE ANSWER QUESTIONS C6 TO C9 ABOUT THE FIRST THEORY TEST THAT YOU TOOK

C6 Had you driven at all before taking your first theory test?

Yes ☐

No ☐ ➡ ***Go to C8***

C7 How many hours driving experience had you had in total when you took your first theory test? Please include both professional lessons and driving practice with friends or relations.

__________ **hours**

C8 In your opinion, how ready to apply for your practical driving test were you when you took your theory test?

(Tick ONE box only)

I was ready to apply for my practical driving test ☐

I was nearly ready to apply for my practical driving test ☐

I was not at all ready to apply for my practical driving test ☐

For office use only

C9 Which of the following did your professional driving instructor do…

(Tick ALL that apply)

	(i) when preparing for your theory test	(ii) between your theory test and practical test
Taught or tested you on knowledge of road signs and markings	1	1
Taught or tested you on other driving- related knowledge	2	2
Taught or tested you on hazard awareness skills	3	3
Recommended particular driving-related books or materials	4	4
Went through example theory test questions	5	5
Did not have any lessons with a professional driving instructor	6	6
Other (*please specify*) ________________	7	7
Nothing	8	8

C10 Which of the following did you use when preparing for your theory and practical driving tests?

(Tick ALL that apply)

	(i) Theory test: Multiple Choice part	(i) Theory test: Hazard Perception part (if included in your theory test)	(ii) Practical test
DSA book 'Official theory Test for Car Drivers'	1	1	1
DSA book 'The Driving Manual'/'Driving-the essential skills'	2	2	2
DSA 'Roadsense'	3	3	3
Highway code	4	4	4
'Know your traffic signs' book	5	5	5
Other books or sets of example questions	6	6	6
An interactive video, CD or DVD based at a driving school	7	7	7
Other driving related video(s), CD(s) or DVD(s)	8	8	8
A website	9	9	9
Other (*please specify*) ________________	10	10	10
I did not use any material to prepare for the test	11	11	11

For office use only

SECTION D: ATTITUDES TOWARDS DRIVING

D1 The following questions ask about your confidence in your own driving ability.

(*Please tick ONE box per line*)

		Very confident	Fairly confident	Not very confident	Not at all confident
**(a)**	How confident were you in your driving ability when you were learning to drive?	☐	☐	☐	☐
**(b)**	In the week before you took your practical driving test, how confident were you that you would pass?	☐	☐	☐	☐
**(c)**	In the week before you took your practical driving test, how confident were you that you were a good enough driver to drive on the road unsupervised?	☐	☐	☐	☐
**(d)**	How confident are you in your driving ability now?	☐	☐	☐	☐

D2 How much do you think you need to improve your ability on each of the following driving skills?

(Tick ONE box per line)

		No improvement needed	Some improvement needed	A lot of improvement needed
**(a)**	Use of car controls	☐	☐	☐
**(b)**	Pulling out of junctions	☐	☐	☐
**(c)**	Reversing	☐	☐	☐
**(d)**	Parking	☐	☐	☐
**(e)**	Judging the speed of other traffic	☐	☐	☐
**(f)**	Judging what other drivers are going to do	☐	☐	☐
**(g)**	Spotting hazards	☐	☐	☐
**(h)**	Driving in heavy traffic	☐	☐	☐
**(i)**	Driving in the dark	☐	☐	☐
**(j)**	Overtaking	☐	☐	☐
**(k)**	Using roundabouts	☐	☐	☐
**(l)**	Joining with moving traffic on a motorway or fast dual carriageway	☐	☐	☐
**(m)**	Changing lanes on a motorway or fast dual carriageway	☐	☐	☐
**(n)**	Driving on high speed roads	☐	☐	☐
**(o)**	Driving on country roads	☐	☐	☐

For office use only

D3 How much do you agree or disagree with each of the following statements?

(Tick ONE box on each line)

	Strongly agree (1)	Agree (2)	Neither agree nor disagree (3)	Disagree (4)	Strongly disagree (5)
**(a)** Decreasing the motorway speed limit is a good idea					
**(b)** Even at night-time on quiet roads it is important to keep within the speed limit					
**(c)** Drivers who cause accidents by reckless driving should be banned from driving for life					
**(d)** People should drive slower than the speed limit when it is raining					
**(e)** Cars should never overtake on the inside lane even if a slow driver is blocking the outside lane					
**(f)** In towns where there are a lot of pedestrians the speed limit should be 20mph					
**(g)** Penalties for speeding should be more severe					
**(h)** Increasing the motorway speed limit is a good idea					

D4 We would like to know what kind of driver you think you are by putting a tick somewhere on each of the lines below.

At either end of each line there is a word that describes a way of driving, and these words are opposites. Put your tick nearer to the word that best describes your driving. The closer your tick is to the word, the more you agree with this description of the way you drive.

	(1)	(2)	(3)	(4)	(5)	(6)	(7)	
Attentive								Inattentive
Careful								Careless
Decisive								Indecisive
Experienced								Inexperienced
Irritable								Placid
Nervous								Confident
Patient								Impatient
Responsible								Irresponsible
Safe								Risky
Selfish								Considerate
Slow								Fast
Tolerant								Intolerant

For office use only

SECTION E: CONTACT DETAILS

For the success of this important research, we would like to get in touch with you again to see how your driving is going.

Please fill in as many of the contact details below as possible.

All information will be treated in the STRICTEST CONFIDENCE and will not be used for any purpose other than to get in touch with you in relation to this research.

E1 Home phone number: ____________________

E2 Work phone number: ____________________
(*if applicable*)

E3 Mobile phone number: ____________________

E4 Email address: ____________________

If the address that this questionnaire was sent to is incorrect, or if there is a better address to help us contact you in the future, please provide the new address in the space below.

E5 New address: ____________________

____

____

____

Postcode: ____________________

E6 If you have any further comments to make about this survey and the issues raised, please write in the space provided:

E7 Please now return this questionnaire in the pre-paid envelope to:
NFER, The Mere, Upton Park, Slough, Berkshire, SL1 2DQ

Please return this questionnaire even if you have only answered the first few questions, as this information is equally important to us and will allow us to remove your name from our mailing list for future questionnaires.

Thank you very much for your help.

LEARNING TO DRIVE QUESTIONNAIRE

Please complete this questionnaire by ticking the appropriate boxes and filling in the spaces as required. Several questions ask for dates or lengths of time etc, if you are unable to give an exact answer to any of these questions please give as good an estimate as you can. It will only take a few minutes to complete this questionnaire and any information you provide will be treated in the **strictest confidence** and will be used for statistical purposes only.

SECTION A: YOUR PRACTICAL DRIVING TEST

A1 Did you take your practical driving test in the week beginning Monday 9th May 2005?

Yes ☐ ➡ ***Go to A2***

No ☐ ➡ ***Go to E7***

A2 When you took your practical driving test in the week beginning Monday 9th May 2005 what type of licence did you hold?

(Tick ONE box only)

Provisional licence to drive a car ☐ ➡ ***Go to A3***

Full licence for another class of vehicle ☐ ➡ ***Go to E7***

A3 Had you previously held a FULL (GB or non- GB) car driving licence before taking your practical driving test in the week beginning Monday 9th May 2005?

(Tick ONE box only)

NO ☐ ➡ ***Go to A4***

YES – Full GB licence ☐ ➡ ***Go to E7***

YES – Full non- GB licence ☐ ➡ ***Go to E7***

For office use only

A4 How old were you when you got your first provisional car licence?

____________ years ____________ months

A5 Before you started learning to drive a car did you regularly ride a motorcycle, moped, or scooter?

Yes ☐

No ☐

A6 Did you take your practical driving test in the week beginning Monday 9th May 2005 in:

(Tick ONE box only)

A driving instructor's car? ☐ 1

A car owned by a friend/relation? ☐ 2

Your own car? ☐ 3

Another car? ☐ 4

A7 Was this practical driving test your:

(Tick ONE box only)

First? ☐ 1 ➡ ***Go to A9***

Second? ☐ 2

Third? ☐ 3

Fourth? ☐ 4

More than fourth? ☐ 5

A8 When did you fail the practical driving test before the one in the week beginning Monday 9th May 2005?

Day____________ Month ____________________ Year ____________

A9 Did you pass the practical driving test that you took in the week beginning Monday 9th May 2005?

Yes ☐ ➡ ***Go to B1***

No ☐

A10 Did you agree with the Examiner's decision to fail you?

Yes ☐

No ☐

For office use only

A11 Why do you think you failed on this occasion? ***(Tick ALL that apply)***

You were just 'unlucky' on the day	☐	1
Difficult weather conditions on the day of the test	☐	2
You were not ready for the test	☐	3
Nerves affected your performance	☐	4
Another road user was to blame	☐	5
Your driving was not good enough on the day	☐	6
You could not cope with something unexpected that happened	☐	7
Other (please specify)	☐	8

____

A12 Since failing the practical driving test in the week beginning Monday 9th May 2005, have you applied for another test?

Yes ☐

No ☐

A13 Before your next test do you think that you will... **(*Tick ONE box only*)**

Have professional driving lessons MORE OFTEN?	☐	1
Have professional driving lessons AS OFTEN AS BEFORE?	☐	2
Have professional driving lessons LESS OFTEN?	☐	3
START having professional driving lessons?	☐	4
NOT HAVE ANY professional driving lessons?	☐	5

A14 Before your next test do you think that you will... **(*Tick ONE box only*)**

Have practice driving sessions with friends or relations MORE OFTEN?	☐	1
Have practice driving sessions with friends or relations AS OFTEN AS BEFORE?	☐	2
Have practice driving sessions with friends or relations LESS OFTEN?	☐	3
START having practice driving sessions with friends or relations?	☐	4
NOT HAVE ANY practice driving sessions with friends or relations?	☐	5

For office use only

SECTION B: LEARNING TO DRIVE

B1 When did you begin to learn to drive a car?

Month ______________________ Year ____________

B2 Since you started learning to drive, was there any period of longer than 6 months during which you did no driving at all?

Yes ☐

No ☐ ➡ *Go to B4*

B3 What was the total of all those periods during which you did no driving at all since you started learning to drive?

____________ years ____________ months

B4 How many miles IN TOTAL had you driven a car before you took your practical driving test in the week beginning Monday 9th May 2005?
(If you are not sure, please give your best estimate to the nearest 100 miles)

____________ miles

B5 Please estimate how your total driving time was split. ***(Tick ONE box only)***

All in a DRIVING INSTRUCTOR'S car	Almost all in a DRIVING INSTRUCTOR'S car	More than half in a DRIVING INSTRUCTOR'S car	About half and half	More than half in OTHER car(s)	Almost all in OTHER car(s)	All in OTHER car(s)
(1)	(2)	(3)	(4)	(5)	(6)	(7)
☐	☐	☐	☐	☐	☐	☐

B6 How many hours of lessons from a professional driving instructor have you had:

(a) IN TOTAL since you first started learning to drive?

____________ hours **None** ☐ ➡ ***If none, please go to B11***

(b) Between the practical driving test that you took in the week beginning Monday 9th May 2005 and your previous test?

____________ hours No previous practical driving test ☐

B7 How many different professional driving instructors have you ever had lessons from?

____________ (Please write number of instructors)

For office use only

B8 What was your main reason for choosing your most recent instructor/driving school?

(Tick ONE box only)

Good reputation	☐	1
Personal recommendation	☐	2
You already knew the person	☐	3
Cost of lessons	☐	4
Local advertising	☐	5
Large company/organisation	☐	6
You saw them in the phone book	☐	7
Highly qualified instructors	☐	8
Other (*please specify*)	☐	9

____

B9 Thinking about ALL the professional driving lessons that you took from when you started driving up to the time of your driving test in the week beginning Monday 9th May 2005, how much have you driven...

(Tick ONE box per line)

		Never	Less than 2 hours	2-4 hours	More than 4 hours
**(a)**	In a busy town centre	☐	☐	☐	☐
**(b)**	On country roads	☐	☐	☐	☐
**(c)**	On fast dual carriageways	☐	☐	☐	☐
**(d)**	In the dark	☐	☐	☐	☐
**(e)**	In the rain	☐	☐	☐	☐
**(f)**	On snow or ice	☐	☐	☐	☐

B10 When you were preparing for the practical driving test you took in the week beginning Monday 9th May 2005, how did you spread out your professional lessons?

(*Tick ONE box only*)

Regularly up to the practical driving test just taken	☐	1
Most at the beginning with a few near the test	☐	2
Most near the test with a few at the beginning	☐	3
Some at the beginning and some near the test with a few in between	☐	4
Few at the beginning and few near the test with most in between	☐	5
Other (*please specify*)	☐	6

____

For office use only

B11 How many hours did you spend practising your driving with friends or relations?

(a) None ☐ ➡ ***If none, please go to B14***

(b) IN TOTAL since you first started learning to drive?

_______________ hours

(c) Between the practical driving test that you took in the week beginning Monday 9th May 2005 and your previous car driving test?

_______________ hours No previous practical driving test ☐

B12 Thinking about ALL the practice driving with friends and relatives that you did from when you started driving up to the time of your driving test in the week beginning Monday 9th May 2005, how much have you driven...

(Tick ONE box per line)

	Never	Less than 2 hours	2-4 hours	More than 4 hours
**(a)** In a busy town centre	☐	☐	☐	☐
**(b)** On country roads	☐	☐	☐	☐
**(c)** On fast dual carriageways	☐	☐	☐	☐
**(d)** In the dark	☐	☐	☐	☐
**(e)** In the rain	☐	☐	☐	☐
**(f)** On snow or ice	☐	☐	☐	☐

B13 When you were preparing for the practical driving test you took in the week beginning Monday 9th May 2005, how did you spread out your sessions of driving practice with friends and relations?

(Tick ONE box only)

Regularly up t the practical driving test just taken ☐ 1

Most at the bginning with a few near the test ☐ 2

Most near the test with a few at the beginning ☐ 3

Some at the beginning and some near the test with a few in between ☐ 4

Few at the beginning and few near the test with most in between ☐ 5

Other (*please specify*) ☐ 6

____

For office use only

B14 When you were preparing for the practical driving test you took in the week beginning Monday 9th May 2005:

		Yes	No
**(a)**	Was it hard to find a friend or relation to take you out for driving practice?		
**(b)**	Were you able to have as much driving practice with friends or relations as you wanted?		
**(c)**	Was it difficult to find time to fit in driving practice with friends or relations?		
**(d)**	Did the cost of professional lessons affect the number you took?		
**(e)**	Do you feel that you had professional lessons often enough?		
**(f)**	Was it difficult to find time to fit in professional lessons?		
**(g)**	When you applied for the test did you think you had a good chance of passing it?		
**(h)**	When you took the test did you think you had a good chance of passing it?		
**(i)**	Were you keen to take your test as soon as possible?		
**(j)**	Did you find learning to drive easy?		

B15 When you were learning to drive did you:

(*Tick ALL that apply*)

Have a DSA Driver's Record that was sent from DVLA with your licence?	☐ 1	
Have a DSA Driver's Record that you got from DSA?	☐ 2	
Have a DSA Driver's Record from your driving instructor?	☐ 3	
Have a different driver's record provided by your instructor?	☐ 4	
(*please specify*) ______________________________		
Not have any written record of your progress while learning to drive.	☐ 5	➡ ***Go to C1***

B16 When you were learning to drive:

(*Tick ONE box per line*)

		Most of the time	Some of the time	Rarely	Never
**(a)**	How often did you use a DSA Driver's Record?				
**(b)**	How often did you use a different driver's record?				

For office use only

SECTION C: YOUR THEORY TEST

C1 When did you pass your theory test for car drivers?

________________ month ________ year

C2 Did the theory test you passed include a Hazard Perception test using film clips?

Yes ☐

No ☐

C3 Was this the first time you had taken the theory test for car drivers?

Yes ☐ ➡ ***Go to C6***

No ☐

C4 (a) In total, how many times have you taken the theory test for car drivers?

________ times

(b) When did you take your FIRST theory test for car drivers?

________________ month ________ year

C5 Did the FIRST theory test you took include a Hazard Perception test using film clips?

Yes ☐

No ☐

PLEASE ANSWER QUESTIONS C6 TO C9 ABOUT THE FIRST THEORY TEST THAT YOU TOOK

C6 Had you driven at all before taking your first theory test?

Yes ☐

No ☐ ➡ ***Go to C8***

C7 How many hours driving experience had you had in total when you took your first theory test? Please include both professional lessons and driving practice with friends or relations.

________ **hours**

C8 In your opinion, how ready to apply for your practical driving test were you when you took your theory test?

(Tick ONE box only)

I was ready to apply for my practical driving test ☐

I was nearly ready to apply for my practical driving test ☐

I was not at all ready to apply for my practical driving test ☐

For office use only

C9 Which of the following did your professional driving instructor do…

(Tick ALL that apply)

	(i) when preparing for your theory test	(ii) between your theory test and practical test
Taught or tested you on knowledge of road signs and markings	1	1
Taught or tested you on other driving- related knowledge	2	2
Taught or tested you on hazard awareness skills	3	3
Recommended particular driving-related books or materials	4	4
Went through example theory test questions	5	5
Did not have any lessons with a professional driving instructor	6	6
Other (*please specify*) ________________	7	7
Nothing	8	8

C10 Which of the following did you use when preparing for your theory and practical driving tests?

(Tick ALL that apply)

	(i) Theory test: Multiple Choice part	(i) Theory test: Hazard Perception part (if included in your theory test)	(ii) Practical test
DSA book 'Official theory Test for Car Drivers'	1	1	1
DSA book 'The Driving Manual'/'Driving-the essential skills'	2	2	2
DSA 'Roadsense'	3	3	3
Highway code	4	4	4
'Know your traffic signs' book	5	5	5
Other books or sets of example questions	6	6	6
An interactive video, CD or DVD based at a driving school	7	7	7
Other driving related video(s), CD(s) or DVD(s)	8	8	8
A website	9	9	9
Other (*please specify*) ________________	10	10	10
I did not use any material to prepare for the test	11	11	11

For office use only

SECTION D: ATTITUDES TOWARDS DRIVING

D1 The following questions ask about your confidence in your own driving ability.

(***Please tick ONE box per line***)

	Very confident	Fairly confident	Not very confident	Not at all confident
**(a)** How confident were you in your driving ability when you were learning to drive?	☐	☐	☐	☐
**(b)** In the week before you took your practical driving test, how confident were you that you would pass?	☐	☐	☐	☐
**(c)** In the week before you took your practical driving test, how confident were you that you were a good enough driver to drive on the road unsupervised?	☐	☐	☐	☐
**(d)** How confident are you in your driving ability now?	☐	☐	☐	☐

D2 How much do you think you need to improve your ability on each of the following driving skills?

(Tick ONE box per line)

	No improvement needed	Some improvement needed	A lot of improvement needed
**(a)** Use of car controls	☐	☐	☐
**(b)** Pulling out of junctions	☐	☐	☐
**(c)** Reversing	☐	☐	☐
**(d)** Parking	☐	☐	☐
**(e)** Judging the speed of other traffic	☐	☐	☐
**(f)** Judging what other drivers are going to do	☐	☐	☐
**(g)** Spotting hazards	☐	☐	☐
**(h)** Driving in heavy traffic	☐	☐	☐
**(i)** Driving in the dark	☐	☐	☐
**(j)** Overtaking	☐	☐	☐
**(k)** Using roundabouts	☐	☐	☐
**(l)** Joining with moving traffic on a motorway or fast dual carriageway	☐	☐	☐
**(m)** Changing lanes on a motorway or fast dual carriageway	☐	☐	☐
**(n)** Driving on high speed roads	☐	☐	☐
**(o)** Driving on country roads	☐	☐	☐

For office use only

D3 How much do you agree or disagree with each of the following statements?

(*Tick ONE box on each line*)

		Strongly agree (1)	Agree (2)	Neither agree nor disagree (3)	Disagree (4)	Strongly disagree (5)
(a)	Decreasing the motorway speed limit is a good idea					
(b)	Even at night-time on quiet roads it is important to keep within the speed limit					
(c)	Drivers who cause accidents by reckless driving should be banned from driving for life					
(d)	People should drive slower than the speed limit when it is raining					
(e)	Cars should never overtake on the inside lane even if a slow driver is blocking the outside lane					
(f)	In towns where there are a lot of pedestrians the speed limit should be 20mph					
(g)	Penalties for speeding should be more severe					
(h)	Increasing the motorway speed limit is a good idea					

D4 We would like to know what kind of driver you think you are by putting a tick somewhere on each of the lines below.

At either end of each line there is a word that describes a way of driving, and these words are opposites. Put your tick nearer to the word that best describes your driving. The closer your tick is to the word, the more you agree with this description of the way you drive.

	(1)	(2)	(3)	(4)	(5)	(6)	(7)	
Attentive								Inattentive
Careful								Careless
Decisive								Indecisive
Experienced								Inexperienced
Irritable								Placid
Nervous								Confident
Patient								Impatient
Responsible								Irresponsible
Safe								Risky
Selfish								Considerate
Slow								Fast
Tolerant								Intolerant

SECTION E: CONTACT DETAILS

For the success of this important research, we would like to get in touch with you again to see how your driving is going.

Please fill in as many of the contact details below as possible.

All information will be treated in the STRICTEST CONFIDENCE and will not be used for any purpose other than to get in touch with you in relation to this research.

E1 Home phone number: ______________________

E2 Work phone number: ______________________
(*if applicable*)

E3 Mobile phone number: ______________________

E4 Email address: ______________________

If the address that this questionnaire was sent to is incorrect, or if there is a better address to help us contact you in the future, please provide the new address in the space below.

E5 New address: ______________________

____

____

____

Postcode: ______________________

E6 If you have any further comments to make about this survey and the issues raised, please write in the space provided:

E7 Please now return this questionnaire in the pre-paid envelope to:
NFER, The Mere, Upton Park, Slough, Berkshire, SL1 2DQ

Please return this questionnaire even if you have only answered the first few questions, as this information is equally important to us and will allow us to remove your name from our mailing list for future questionnaires.

Thank you very much for your help.

Department for Transport

LEARNING TO DRIVE QUESTIONNAIRE

Please complete this questionnaire by ticking the appropriate boxes and filling in the spaces as required. Several questions ask for dates or lengths of time etc, if you are unable to give an exact answer to any of these questions please give as good an estimate as you can. It will only take a few minutes to complete this questionnaire and any information you provide will be treated in the **strictest confidence** and will be used for statistical purposes only.

SECTION A: YOUR PRACTICAL DRIVING TEST

A1 Did you take your practical driving test in the week beginning Monday 8th August 2005?

Yes ☐ ➡ *Go to A2*

No ☐ ➡ *Go to E7*

A2 When you took your practical driving test in the week beginning Monday 8th August 2005 what type of licence did you hold?

(Tick ONE box only)

Provisional licence to drive a car ☐ ➡ *Go to A3*

Full licence for another class of vehicle ☐ ➡ *Go to E7*

A3 Had you previously held a FULL (GB or non- GB) car driving licence before taking your practical driving test in the week beginning Monday 8th August 2005?

(Tick ONE box only)

NO ☐ ➡ *Go to A4*

YES – Full GB licence ☐ ➡ *Go to E7*

YES – Full non- GB licence ☐ ➡ *Go to E7*

A4 How old were you when you got your first provisional car licence?

__________ years __________ months

A5 Before you started learning to drive a car did you regularly ride a motorcycle, moped, or scooter?

Yes ☐

No ☐

A6 Did you take your practical driving test in the week beginning Monday 8th August 2005 in:
(Tick ONE box only)

A driving instructor's car? ☐ 1

A car owned by a friend/relation? ☐ 2

Your own car? ☐ 3

Another car? ☐ 4

A7 Was this practical driving test your:
(Tick ONE box only)

First? ☐ 1 ➡ ***Go to A9***

Second? ☐ 2

Third? ☐ 3

Fourth? ☐ 4

More than fourth? ☐ 5

A8 When did you fail the practical driving test before the one in the week beginning Monday 8th August 2005?

Day__________ Month ____________________ Year __________

A9 Did you pass the practical driving test that you took in the week beginning Monday 8th August 2005?

Yes ☐ ➡ ***Go to B1***

No ☐

A10 Did you agree with the Examiner's decision to fail you?

Yes ☐

No ☐

For office use only

A11 Why do you think you failed on this occasion? *(Tick ALL that apply)*

You were just 'unlucky' on the day	☐	1
Difficult weather conditions on the day of the test	☐	2
You were not ready for the test	☐	3
Nerves affected your performance	☐	4
Another road user was to blame	☐	5
Your driving was not good enough on the day	☐	6
You could not cope with something unexpected that happened	☐	7
Other (please specify)	☐	8

____

A12 Since failing the practical driving test in the week beginning Monday 8th August 2005, have you applied for another test?

Yes	☐
No	☐

A13 Before your next test do you think that you will... (*Tick ONE box only*)

Have professional driving lessons MORE OFTEN?	☐	1
Have professional driving lessons AS OFTEN AS BEFORE?	☐	2
Have professional driving lessons LESS OFTEN?	☐	3
START having professional driving lessons?	☐	4
NOT HAVE ANY professional driving lessons?	☐	5

A14 Before your next test do you think that you will... (*Tick ONE box only*)

Have practice driving sessions with friends or relations MORE OFTEN?	☐	1
Have practice driving sessions with friends or relations AS OFTEN AS BEFORE?	☐	2
Have practice driving sessions with friends or relations LESS OFTEN?	☐	3
START having practice driving sessions with friends or relations?	☐	4
NOT HAVE ANY practice driving sessions with friends or relations?	☐	5

For office use only

SECTION B: LEARNING TO DRIVE

B1 When did you begin to learn to drive a car?

Month ______________________ Year ____________

B2 Since you started learning to drive, was there any period of longer than 6 months during which you did no driving at all?

Yes ☐

No ☐ ➡ ***Go to B4***

B3 What was the total of all those periods during which you did no driving at all since you started learning to drive?

____________ years ____________ months

B4 How many miles IN TOTAL had you driven a car before you took your practical driving test in the week beginning Monday 8th August 2005?
(If you are not sure, please give your best estimate to the nearest 100 miles)

____________ miles

B5Please estimate how your total driving time was split. ***(Tick ONE box only)***

All in a DRIVING INSTRUCTOR'S car	Almost all in a DRIVING INSTRUCTOR'S car	More than half in a DRIVING INSTRUCTOR'S car	About half and half	More than half in OTHER car(s)	Almost all in OTHER car(s)	All in OTHER car(s)
(1)	(2)	(3)	(4)	(5)	(6)	(7)
☐	☐	☐	☐	☐	☐	☐

B6 How many hours of lessons from a professional driving instructor have you had:

(a) IN TOTAL since you first started learning to drive?

____________ hours **None** ☐ ➡ ***If none, please go to B11***

(b) Between the practical driving test that you took in the week beginning Monday 8th August 2005 and your previous test?

____________ hours No previous practical driving test ☐

B7 How many different professional driving instructors have you ever had lessons from?

____________ (Please write number of instructors)

For office use only

B8 What was your main reason for choosing your most recent instructor/driving school?

(Tick ONE box only)

Good reputation	☐	1
Personal recommendation	☐	2
You already knew the person	☐	3
Cost of lessons	☐	4
Local advertising	☐	5
Large company/organisation	☐	6
You saw them in the phone book	☐	7
Highly qualified instructors	☐	8
Other (*please specify*)	☐	9

____

B9 Thinking about ALL the professional driving lessons that you took from when you started driving up to the time of your driving test in the week beginning Monday 8th August 2005, how much have you driven...

(Tick ONE box per line)

		Never	Less than 2 hours	2-4 hours	More than 4 hours
**(a)**	In a busy town centre	☐	☐	☐	☐
**(b)**	On country roads	☐	☐	☐	☐
**(c)**	On fast dual carriageways	☐	☐	☐	☐
**(d)**	In the dark	☐	☐	☐	☐
**(e)**	In the rain	☐	☐	☐	☐
**(f)**	On snow or ice	☐	☐	☐	☐

B10 When you were preparing for the practical driving test you took in the week beginning Monday 8th August 2005, how did you spread out your professional lessons?

(*Tick ONE box only*)

Regularly up to the practical driving test just taken	☐	1
Most at the beginning with a few near the test	☐	2
Most near the test with a few at the beginning	☐	3
Some at the beginning and some near the test with a few in between	☐	4
Few at the beginning and few near the test with most in between	☐	5
Other (*please specify*)	☐	6

____

For office use only

B11 How many hours did you spend practising your driving with friends or relations?

(a) **None** ☐ ➡ ***If none, please go to B14***

(b) **IN TOTAL since you first started learning to drive?**

_______________ hours

(c) **Between the practical driving test that you took in the week beginning Monday 8th August 2005 and your previous car driving test?**

_______________ hours No previous practical driving test ☐

B12 Thinking about ALL the practice driving with friends and relatives that you did from when you started driving up to the time of your driving test in the week beginning Monday 8th August 2005, how much have you driven...

(Tick ONE box per line)

	Never	Less than 2 hours	2-4 hours	More than 4 hours
**(a)** In a busy town centre	☐	☐	☐	☐
**(b)** On country roads	☐	☐	☐	☐
**(c)** On fast dual carriageways	☐	☐	☐	☐
**(d)** In the dark	☐	☐	☐	☐
**(e)** In the rain	☐	☐	☐	☐
**(f)** On snow or ice	☐	☐	☐	☐

B13 When you were preparing for the practical driving test you took in the week beginning Monday 8th August 2005, how did you spread out your sessions of driving practice with friends and relations?

(Tick ONE box only)

- Regularly up to the practical driving test just taken ☐ 1
- Most at the beginning with a few near the test ☐ 2
- Most near the test with a few at the beginning ☐ 3
- Some at the beginning and some near the test with a few in between ☐ 4
- Few at the beginning and few near the test with most in between ☐ 5
- Other (*please specify*) ☐ 6

____

B14 When you were preparing for the practical driving test you took in the week beginning Monday 8th August 2005:

	Yes	No
**(a)** Was it hard to find a friend or relation to take you out for driving practice?	☐	☐
**(b)** Were you able to have as much driving practice with friends or relations as you wanted?	☐	☐
**(c)** Was it difficult to find time to fit in driving practice with friends or relations?	☐	☐
**(d)** Did the cost of professional lessons affect the number you took?	☐	☐
**(e)** Do you feel that you had professional lessons often enough?	☐	☐
**(f)** Was it difficult to find time to fit in professional lessons?	☐	☐
**(g)** When you applied for the test did you think you had a good chance of passing it?	☐	☐
**(h)** When you took the test did you think you had a good chance of passing it?	☐	☐
**(i)** Were you keen to take your test as soon as possible?	☐	☐
**(j)** Did you find learning to drive easy?	☐	☐

B15 When you were learning to drive did you:

(*Tick ALL that apply*)

Have a DSA Driver's Record that was sent from DVLA with your licence? ☐ 1

Have a DSA Driver's Record that you got from DSA? ☐ 2

Have a DSA Driver's Record from your driving instructor? ☐ 3

Have a different driver's record provided by your instructor? ☐ 4

(*please specify*) ____________________

Not have any written record of your progress while learning to drive. ☐ 5 ➡ ***Go to C1***

B16 When you were learning to drive:

(*Tick ONE box per line*)

	Most of the time	Some of the time	Rarely	Never
**(a)** How often did you use a DSA Driver's Record?	☐	☐	☐	☐
**(b)** How often did you use a different driver's record?	☐	☐	☐	☐

For office use only

SECTION C: YOUR THEORY TEST

C1 When did you pass your theory test for car drivers?

________________ month ________ year

C2 Did the theory test you passed include a Hazard Perception test using film clips?

Yes ☐

No ☐

C3 Was this the first time you had taken the theory test for car drivers?

Yes ☐ ➡ ***Go to C6***

No ☐

C4 (a) In total, how many times have you taken the theory test for car drivers?

________ times

(b) When did you take your FIRST theory test for car drivers?

________________ month ________ year

C5 Did the FIRST theory test you took include a Hazard Perception test using film clips?

Yes ☐

No ☐

PLEASE ANSWER QUESTIONS C6 TO C9 ABOUT THE FIRST THEORY TEST THAT YOU TOOK

C6 Had you driven at all before taking your first theory test?

Yes ☐

No ☐ ➡ ***Go to C8***

C7 How many hours driving experience had you had in total when you took your first theory test? Please include both professional lessons and driving practice with friends or relations.

________ **hours**

C8 In your opinion, how ready to apply for your practical driving test were you when you took your theory test?

(Tick ONE box only)

I was ready to apply for my practical driving test ☐

I was nearly ready to apply for my practical driving test ☐

I was not at all ready to apply for my practical driving test ☐

For office use only

C9 Which of the following did your professional driving instructor do…

(Tick ALL that apply)

	(i) when preparing for your theory test	(ii) between your theory test and practical test
Taught or tested you on knowledge of road signs and markings	☐ 1	☐ 1
Taught or tested you on other driving- related knowledge	☐ 2	☐ 3
Taught or tested you on hazard awareness skills	☐ 3	☐ 3
Recommended particular driving-related books or materials	☐ 4	☐ 4
Went through example theory test questions	☐ 5	☐ 5
Did not have any lessons with a professional driving instructor	☐ 6	☐ 6
Other (*please specify*) ________________	☐ 7	☐ 7
Nothing	☐ 8	☐ 8

C10 Which of the following did you use when preparing for your theory and practical driving tests?

(Tick ALL that apply)

	(i) Theory test		(ii) Practical test
	Multiple Choice part	Hazard Perception part (if included in your theory test)	
DSA book 'Official theory Test for Car Drivers'	☐ 1	☐ 1	☐ 1
DSA book 'The Driving Manual'/'Driving-the essential skills'	☐ 2	☐ 2	☐ 2
DSA 'Roadsense'	☐ 2	☐ 2	☐ 2
Highway code	☐ 2	☐ 2	☐ 2
'Know your traffic signs' book	☐ 2	☐ 2	☐ 2
Other books or sets of example questions	☐ 2	☐ 2	☐ 2
An interactive video, CD or DVD based at a driving school	☐ 2	☐ 2	☐ 2
Other driving related video(s), CD(s) or DVD(s)	☐ 2	☐ 2	☐ 2
A website	☐ 2	☐ 2	☐ 2
Other (*please specify*) ________________	☐ 2	☐ 2	☐ 2
I did not use any material to prepare for the test	☐ 2	☐ 2	☐ 2

For office use only

SECTION D: ATTITUDES TOWARDS DRIVING

D1 The following questions ask about your confidence in your own driving ability.

(*Please tick ONE box per line*)

	Very confident	Fairly confident	Not very confident	Not at all confident
**(a)** How confident were you in your driving ability when you were learning to drive?	☐	☐	☐	☐
**(b)** In the week before you took your practical driving test, how confident were you that you would pass?	☐	☐	☐	☐
**(c)** In the week before you took your practical driving test, how confident were you that you were a good enough driver to drive on the road unsupervised?	☐	☐	☐	☐
**(d)** How confident are you in your driving ability now?	☐	☐	☐	☐

D2 How much do you think you need to improve your ability on each of the following driving skills?

(Tick ONE box per line)

	No improvement needed	Some improvement needed	A lot of improvement needed
**(a)** Use of car controls	☐	☐	☐
**(b)** Pulling out of junctions	☐	☐	☐
**(c)** Reversing	☐	☐	☐
**(d)** Parking	☐	☐	☐
**(e)** Judging the speed of other traffic	☐	☐	☐
**(f)** Judging what other drivers are going to do	☐	☐	☐
**(g)** Spotting hazards	☐	☐	☐
**(h)** Driving in heavy traffic	☐	☐	☐
**(i)** Driving in the dark	☐	☐	☐
**(j)** Overtaking	☐	☐	☐
**(k)** Using roundabouts	☐	☐	☐
**(l)** Joining with moving traffic on a motorway or fast dual carriageway	☐	☐	☐
**(m)** Changing lanes on a motorway or fast dual carriageway	☐	☐	☐
**(n)** Driving on high speed roads	☐	☐	☐
**(o)** Driving on country roads	☐	☐	☐

For office use only

D3 How much do you agree or disagree with each of the following statements?

(*Tick ONE box on each line*)

	Strongly agree (1)	Agree (2)	Neither agree nor disagree (3)	Disagree (4)	Strongly disagree (5)
**(a)** Decreasing the motorway speed limit is a good idea	☐	☐	☐	☐	☐
**(b)** Even at night-time on quiet roads it is important to keep within the speed limit	☐	☐	☐	☐	☐
**(c)** Drivers who cause accidents by reckless driving should be banned from driving for life	☐	☐	☐	☐	☐
**(d)** People should drive slower than the speed limit when it is raining	☐	☐	☐	☐	☐
**(e)** Cars should never overtake on the inside lane even if a slow driver is blocking the outside lane	☐	☐	☐	☐	☐
**(f)** In towns where there are a lot of pedestrians the speed limit should be 20mph	☐	☐	☐	☐	☐
**(g)** Penalties for speeding should be more severe	☐	☐	☐	☐	☐
**(h)** Increasing the motorway speed limit is a good idea	☐	☐	☐	☐	☐

D4 We would like to know what kind of driver you think you are by putting a tick somewhere on each of the lines below.

At either end of each line there is a word that describes a way of driving, and these words are opposites. Put your tick nearer to the word that best describes your driving. The closer your tick is to the word, the more you agree with this description of the way you drive.

	(1)	(2)	(3)	(4)	(5)	(6)	(7)	
Attentive	☐	☐	☐	☐	☐	☐	☐	Inattentive
Careful	☐	☐	☐	☐	☐	☐	☐	Careless
Decisive	☐	☐	☐	☐	☐	☐	☐	Indecisive
Experienced	☐	☐	☐	☐	☐	☐	☐	Inexperienced
Irritable	☐	☐	☐	☐	☐	☐	☐	Placid
Nervous	☐	☐	☐	☐	☐	☐	☐	Confident
Patient	☐	☐	☐	☐	☐	☐	☐	Impatient
Responsible	☐	☐	☐	☐	☐	☐	☐	Irresponsible
Safe	☐	☐	☐	☐	☐	☐	☐	Risky
Selfish	☐	☐	☐	☐	☐	☐	☐	Considerate
Slow	☐	☐	☐	☐	☐	☐	☐	Fast
Tolerant	☐	☐	☐	☐	☐	☐	☐	Intolerant

For office use only

SECTION E: CONTACT DETAILS

For the success of this important research, we would like to get in touch with you again to see how your driving is going.

Please fill in as many of the contact details below as possible.

All information will be treated in the STRICTEST CONFIDENCE and will not be used for any purpose other than to get in touch with you in relation to this research.

E1 Home phone number: ______________________

E2 Work phone number: ______________________
(*if applicable*)

E3 Mobile phone number: ______________________

E4 Email address: ______________________

If the address that this questionnaire was sent to is incorrect, or if there is a better address t[illegible] help us contact you in the future, please provide the new address in the space below.

E5 New address: ______________________

____

____

____

Postcode: ______________________

E6 If you have any further comments to make about this survey and the issues raised, please write in the space provided:

E7 Please now return this questionnaire in the pre-paid envelope to:
NFER, The Mere, Upton Park, Slough, Berkshire, SL1 2DQ

Please return this questionnaire even if you have only answered the first few questions, as this information is equally important to us and will allow us to remove your name from our mailing list for future questionnaires.

Thank you very much for your help.

DRIVING EXPERIENCE QUESTIONNAIRE

This questionnaire asks you about your driving in the 6 months since passing your driving test. Please answer the questions by ticking the appropriate boxes and writing in the spaces as required. Please read carefully the instructions telling you which questions you should answer next. It may be that only a few of the questions apply to you. This questionnaire will only take a few minutes to complete and any information you provide will be treated in the **strictest confidence** and will be used for statistical purposes only.

SECTION A: YOUR DRIVING

A1 On average, how often did you drive in last 6 months?

(Tick ONE box only)

Every day	☐ 1	
4-6 days a week	☐ 2	
1-3 days a week	☐ 3	
About once a fortnight	☐ 4	
About once a month	☐ 5	
Less than once a month	☐ 6	
Never	☐ 7	➡ *Go to E1*

A2 About how many miles did you drive in a car or van in the last 6 months? (If you are not certain, please give as good an estimate as you can) ***(Write in)***

______________________ miles

A3 About how many times did you drive more than 100 miles in a single day in the last 6 months? ***(Write in)***

______________________ times

For office use only

A4 In the last 6 months, how often did you drive…. (*Tick ONE box per line)*

	Never (1)	Less than once a month (2)	About once a fortnight (3)	1-3 days a week (4)	4-6 days a week (5)	Every day (6)
**(a)** In a busy town or city centre						
**(b)** In quiet parts of towns or cities						
**(c)** On country roads						
**(d)** On fast dual carriageways						
**(e)** On motorways						
**(f)** In the dark						
**(g)** In the rain						
(**h**) In fog						
**(i)** In snow or ice						
**(j)** To and from your place of work or study						
**(k)** On your employer's business						

A5 Who owned the vehicle you drove most often in the last 6 months? *(Tick ONE box only)*

- You personally ☐ 1
- Your employer (including cars leased to your employer) ☐ 2
- A member of your family ☐ 3
- A friend ☐ 4
- Some other person/organisation ☐ 5

A6 What further driver training have you had in the last 6 months? (*Tick ALL that apply*)

- None ☐ 1 ➡ ***Go to B1***
- Pass Plus ☐ 2
- Motorway lessons ☐ 3
- Driver training for company car drivers ☐ 4
- Other advanced driver training (e.g IAM, RoSPA, Diamond) ☐ 5
- Other (please specify) ____________________ ☐ 6

A7 Who paid for you to have this training? *(Tick ALL that apply)*

- Yourself ☐ 1
- A member of your family ☐ 2
- Your employer ☐ 3
- Someone else ☐ 4
- It was free ☐ 5

For office use only

SECTION B: ACCIDENTS

NEAR MISSES

B1 Many drivers have had the impression of only just avoiding an accident. How many times has this happened to you in the last 6 months? ***(Tick ONE box only)***

Never	☐	1
1 or 2 times	☐	2
3 to 5 times	☐	3
6 to 10 times	☐	4
More than 10 times	☐	5

ACCIDENTS

B2 How many accidents were you actually involved in when driving a car or van in the last 6 months? (Please include all accidents, regardless of how they were caused, how slight they were or where they happened) ***(Tick ONE box only)***

None	☐	1	➡ ***Go to C1***
One	☐	2	
Two	☐	3	
Three	☐	4	
More than three	☐	5	

Please now give us further details of your accident(s)

B3 When did the accident(s) happen? ***(Please enter time, month and year below)***

Most recent accident:	Time of day: ______ am/pm	Month: ______________	Year: ________
Next most recent:	Time of day: ______ am/pm	Month: ______________	Year: ________
One before that:	Time of day: ______ am/pm	Month: ______________	Year: ________

B4a Did the accident(s) happen ***(Tick ONE box for each accident)***

	Most recent accident	Next most recent	One before that
on a public road?	☐	☐	☐
in a car park, service area or petrol station?	☐	☐	☐
on a private driveway?	☐	☐	☐
on a private road?	☐	☐	☐
somewhere else? *(please specify)* ______________	☐	☐	☐

B4b Would you describe the accident(s) as: a 'low speed manoeuvring accident'? ***(Tick ONE box for each accident)***

Yes	☐	☐	☐
No	☐	☐	☐

B4c Would you describe the accident(s) as: a 'minor bump or scrape'? ***(Tick ONE box for each accident)***

Yes	☐	☐	☐
No	☐	☐	☐

For office use only

We are interested in the type(s) of accident(s) you were involved in while driving a car or van in the last 6 months.

B5 Apart from your vehicle, what else was involved in the accident(s)?

(Tick ALL boxes that apply for each accident)

	Most recent accident	Next most recent	One before that
Nothing/no-one	☐ 1	☐ 1	☐ 1
Other car(s) or van(s)	☐ 2	☐ 2	☐ 2
Motorbike(s)	☐ 3	☐ 3	☐ 3
HGV(s)	☐ 4	☐ 4	☐ 4
Bus(es)	☐ 5	☐ 5	☐ 5
Pedal cyclist(s)	☐ 6	☐ 6	☐ 6
Pedestrian(s)	☐ 7	☐ 7	☐ 7
Roadside objects	☐ 8	☐ 8	☐ 8
Other *(please specify)* ________	☐ 9	☐ 9	☐ 9

B6 What injuries were there to yourself or any others as a result of the accident(s)?

(Tick ONE box for each accident)

None	☐	☐	☐
Slight (e.g. cuts and bruises)	☐	☐	☐
Serious (needing hospital care)	☐	☐	☐

B7 To what extent do you think you were to blame for the accident(s)?

(Tick ONE box for each accident)

Not at all	☐	☐	☐
A little	☐	☐	☐
Quite a lot	☐	☐	☐
Entirely	☐	☐	☐

B8 What was the weather like at the time of the accident(s)?

(Tick ALL boxes that apply for each accident)

Dry	☐	☐	☐
Raining	☐	☐	☐
Fog	☐	☐	☐
Snow or ice on the road	☐	☐	☐

B9 Did the accident(s) happen

(Tick ONE box for each accident)

in daylight?	☐	☐	☐
at dawn or dusk?	☐	☐	☐
in the dark?	☐	☐	☐

For office use only

B10 Did the accident(s) happen *(Tick ONE box for each accident)*

	Most recent accident	Next most recent	One before that
in a town or city centre?	☐	☐	☐
on country roads?	☐	☐	☐
on fast dual carriageways?	☐	☐	☐
on motorways?	☐	☐	☐
somewhere else? *(please specify)* ______	☐	☐	☐

B11 What was the purpose of your journey when you had the accident(s)? *(Tick ONE box for each accident)*

Travelling to or from your place of work or study	☐	☐	☐
Travelling as part of your work	☐	☐	☐
Travelling for personal reasons	☐	☐	☐
Travelling for another reason *(please specify)* ______	☐	☐	☐

B12 How busy were the traffic conditions when the accident(s) happened? *(Tick ONE box for each accident)*

Very busy	☐	☐	☐
Busy	☐	☐	☐
Not very busy	☐	☐	☐
Not at all busy	☐	☐	☐

B13 What happened first in the accident(s)? *(Tick ONE box for each accident)*

Another vehicle hit your vehicle while it was parked	☐ 1	☐ 1	☐ 1
Your vehicle hit a pedestrian	☐ 2	☐ 2	☐ 2
Your vehicle hit a cyclist	☐ 3	☐ 3	☐ 3
Your vehicle hit the rear of another vehicle	☐ 4	☐ 4	☐ 4
Your vehicle hit the side of another vehicle	☐ 5	☐ 5	☐ 5
Another vehicle hit the rear of your vehicle	☐ 6	☐ 6	☐ 6
Another vehicle hit the side of your vehicle	☐ 7	☐ 7	☐ 7
Your vehicle was hit by an on-coming vehicle in *your* lane	☐ 8	☐ 8	☐ 8
Your vehicle was hit by an on-coming vehicle in *their* lane	☐ 9	☐ 9	☐ 9
Your vehicle hit a roadside object	☐ 10	☐ 10	☐ 10
Your vehicle left the road without hitting any other object	☐ 11	☐ 11	☐ 11
Other (*please specify*) ______	☐ 12	☐ 12	☐ 12

For office use only

B14a Apart from yourself were there any other people in your vehicle at the time of the accident(s)?

(Tick ONE box for each accident)

	Most recent accident	Next most recent	One before that
Yes	☐	☐	☐
No	☐	☐	☐

B14b If there were other people in your vehicle how many were MALE?

(Write in for each accident)

________ ________ ________

B14c If there were other people in your vehicle how many were FEMALE?

(Write in for each accident)

________ ________ ________

B14d If there were other people in your vehicle at the time of the accident(s) what was the age of the oldest person?

(Write in for each accident)

________ ________ ________

SECTION C: DRIVING OFFENCES

C1 Many drivers are warned by the Police for motoring offences without any other action being taken.

Apart from parking offences, has this happened to you in the last 6 months?

Yes ☐ No ☐ If *YES*, how many times? ____________ ____

C2 Have you received any fixed penalty notices or summonses for motoring offences, apart from parking offences, in the last 6 months?

Yes ☐ No ☐ If *YES*, how many times? ____________ ____

C3 Are you waiting for a summons, or waiting to hear whether you might be issued with a summons, for a motoring offence in the last 6 months?

Yes ☐ No ☐ If *YES*, how many times? ____________ ____

C4 Have you been banned from driving during the last 6 months?

Yes ☐ No ☐

C5 Have you been told that you must go back to being a learner because you have too many penalty points on your licence in the last 6 months?

Yes ☐ No ☐

For office use only

SECTION D: YOU AS A DRIVER

D1 Compared with other drivers of your age and sex:

(a) How likely are you to be being involved in an accident when driving a car or van? *(Tick ONE box only)*

- More likely than others ☐
- As likely as others ☐
- Less likely than others ☐

(b) How skilled a driver are you?

- More skilful than others ☐
- As skilful as others ☐
- Less skilful than others ☐

(c) How cautious a driver are you?

- More cautious than others ☐
- As cautious as others ☐
- Less cautious than others ☐

(d) How likely are you to avoid risky driving situations?

- More likely than others ☐
- As likely as others ☐
- Less likely than others ☐

(e) How fast do you drive?

- A little faster than others ☐
- About the same speed as others ☐
- A little slower than others ☐
- Much slower than others ☐

D2 How do you compare your driving to that of other drivers generally? *(Tick ONE box only)*

- Much better than average ☐
- A bit better than average ☐
- About average ☐
- A bit worse than average ☐
- Much worse than average ☐

D3 In general, how confident are you in your driving ability? *(Tick ONE box only)*

- Very confident ☐
- Fairly confident ☐
- Not very confident ☐
- Not at all confident ☐

D4 How much do you think you need to improve your ability on each of the following driving skills? ***(Tick ONE box per line)***

	No improvement needed (1)	Some improvement needed (2)	A lot of improvement needed (3)
**(a)** Use of car controls	☐	☐	☐
**(b)** Pulling out of junctions	☐	☐	☐
**(c)** Reversing	☐	☐	☐
**(d)** Parking	☐	☐	☐
**(e)** Judging the speed of other traffic	☐	☐	☐
**(f)** Judging what other drivers are going to do	☐	☐	☐
**(g)** Spotting hazards	☐	☐	☐
**(h)** Driving in heavy traffic	☐	☐	☐
**(i)** Driving in the dark	☐	☐	☐
**(j)** Overtaking	☐	☐	☐
**(k)** Using roundabouts	☐	☐	☐
**(l)** Joining with moving traffic on a motorway or fast dual carriageway	☐	☐	☐
**(m)** Changing lanes on a motorway or fast dual carriageway	☐	☐	☐
**(n)** Driving on high speed roads	☐	☐	☐
**(o)** Driving on country roads	☐	☐	☐
**(p)** Driving in heavy rain	☐	☐	☐
**(q)** Driving in thick fog	☐	☐	☐
**(r)** Driving on snow or ice	☐	☐	☐
**(s)** Turning right	☐	☐	☐
**(t)** Knowing what speed is safe	☐	☐	☐

D5 How often do you exceed speed limits? ***(Tick ONE box only)***

- Never ☐ 1
- Rarely ☐ 2
- Occasionally ☐ 3
- Fairly often ☐ 4
- Very often ☐ 5
- Always ☐ 6

For office use only

D6 When driving, how often do you do each of the following?

(*Tick ONE box on each line*)

	Never (1)	Hardly ever (2)	Occas-ionally (3)	Quite often (4)	Frequ-ently (5)	Nearly all the time (6)
**(a)** Attempt to drive away from traffic lights in too high a gear						
**(b)** Overtake a slow driver on the inside						
**(c)** Have to confirm you are in the right gear						
**(d)** Attempt to overtake someone you hadn't noticed to be signalling a right turn						
**(e)** Forget where you left your car in a car park						
**(f)** Sound your horn to indicate your annoyance with another road user						
**(g)** Switch on one thing, such as the headlights, when you meant to switch on something else, such as the wipers						
**(h)** Change into the wrong gear when driving along						
**(i)** Pull out of a junction so far that the driver with the right of way has to stop and let you out						
**(j)** Use P Plates or Green L Plates to warn other road users that you are a new driver						

D7 And how often do you do each of these?

(*Tick ONE box on each line*)

	Never (1)	Hardly ever (2)	Occas-ionally (3)	Quite often (4)	Frequ-ently (5)	Nearly all the time (6)
**(a)** Realise you have no clear recollection of the road along which you have just been travelling						
**(b)** Cross a junction knowing that the traffic lights have already turned against you						
**(c)** Fail to notice that pedestrians are crossing when turning into a side street from a main road						
**(d)** Become angered by another driver and give chase with the intention of giving him/her a piece of your mind						
**(e)** Misread signs and take the wrong turning off a roundabout						
**(f)** Drive in either too high or too low a gear for the conditions						
**(g)** Disregard the speed limit on a residential road						
**(h)** On turning left, nearly hit a cyclist who has come up on your inside						
**(I)** Use a mobile phone without a hands free kit						

For office use only

D8 And how often do you do each of these? (*Tick ONE box on each line*)

	Never (1)	Hardly ever (2)	Occas-ionally (3)	Quite often (4)	Frequ-ently (5)	Nearly all the time (6)
**(a)** Stay in a motorway lane that you know will be closed before forcing your way in at the last minute						
**(b)** Queuing to turn left onto a main road, you pay such close attention to the main stream of traffic that you nearly hit the car in front						
**(c)** Drive when you suspect you may be over the legal alcohol limit						
**(d)** Forget to take the handbrake off before moving off						
**(e)** Become angered by a particular type of driver, and indicate your hostility by whatever means you can						
**(f)** Underestimate the speed of an oncoming vehicle when overtaking						
**(g)** Hit something when reversing that you had not previously seen						
**(h)** Race away from traffic lights with the intention of beating the driver next to you						
**(i)** Use a hands free mobile phone						

D9 And how often do you do each of these? (*Tick ONE box on each line*)

	Never (1)	Hardly ever (2)	Occas-ionally (3)	Quite often (4)	Frequ-ently (5)	Nearly all the time (6)
**(a)** Select the wrong gear when wanting to go into reverse						
**(b)** Intending to drive to destination A, you suddenly notice that you are on the road to destination B, perhaps because the latter is your more usual destination						
**(c)** Get into the wrong lane when approaching a roundabout or junction						
**(d)** Drive so close to the car in front that it would be difficult to stop in an emergency						
**(e)** Forget that the headlights are on full beam						
**(f)** Miss *Give Way* signs and narrowly avoid colliding with traffic having the right of way						
**(g)** Disregard the speed limit on a motorway						
**(h)** Fail to check your rear-view mirror before pulling out, changing lanes, etc						
**(i)** Brake too quickly on a slippery road, or steer the wrong way into a skid						
**(j)** Drive after taking drugs when you think you might still be affected by them						

For office use only

D10 When driving, how often do each of the following things happen to you?

(*Tick ONE box on each line*)

	Never (1)	Very rarely (2)	Occas-ionally (3)	Fairly often (4)	Very often (5)	Nearly all the time (6)
**(a)** You have to brake sharply to avoid a collision with the vehicle ahead of you because it has slowed						
**(b)** You pull out to overtake or turn right not noticing another vehicle in your 'blind spot'						
**(c)** You fail to notice someone waiting at a pedestrian crossing						
**(d)** You misjudge the gaps in main road traffic when pulling out of a side road						
**(e)** When cornering, you find you are travelling too fast to negotiate the bend safely and have to brake						
**(f)** You fail to give way when entering a roundabout to a vehicle already on the roundabout						
**(g)** You have to brake or swerve suddenly to avoid an accident						

D11 Please show what kind of driver you think you are by putting a tick somewhere on each of the lines below.

At either end of each line there is a word that describes a way of driving, and these words are opposites. Put your tick nearer to the word that best describes your driving. The closer your tick is to the word, the more you agree with this description of the way you drive.

	(1)	(2)	(3)	(4)	(5)	(6)	(7)	
Attentive								Inattentive
Careful								Careless
Decisive								Indecisive
Experienced								Inexperienced
Irritable								Placid
Nervous								Confident
Patient								Impatient
Responsible								Irresponsible
Safe								Risky
Selfish								Considerate
Slow								Fast
Tolerant								Intolerant

For office use only

SECTION E: YOUR DETAILS

E1 Please tell us when you passed your practical driving test

Month: ______________________ **Year:** _____________

For the success of this important research, we would like to get in touch with you again to see how your driving is going. Please fill in as many of the contact details below as possible. All information will be treated in the **strictest confidence** and will not be used for any purpose other than to get in touch with you in relation to this research.

E2 Home phone number: ______________________________

E3 Work phone number: ______________________________

E4 Mobile phone number: ______________________________

E5 Email address: ______________________________

If the address that this questionnaire was sent to is incorrect, or if there is a better address to help us contact you in the future, please provide the new address in the space below.

E6 New address: ____

____

____

____

____

Postcode: ______________________

E7 If you have any further comments to make about this survey and the issues raised, please write in the space provided:

E8 Please now return this questionnaire in the pre-paid envelope to:

NFER, The Mere, Upton Park, Slough, Berkshire, SL1 2DQ

Please return this questionnaire even if you have only answered the first few questions, as this information is very important to us.

Thank you very much for your help.

DRIVING EXPERIENCE QUESTIONNAIRE

This questionnaire asks you about your driving in the last 6 months. Please answer the questions by ticking the appropriate boxes and writing in the spaces as required. Please read carefully the instructions telling you which questions you should answer next. It may be that only a few of the questions apply to you. This questionnaire will only take a few minutes to complete and any information you provide will be treated in the **strictest confidence** and will be used for statistical purposes only.

SECTION A: YOUR DRIVING

A1 On average, how often did you drive in last 6 months?

(Tick ONE box only)

Every day	☐ 1	
4-6 days a week	☐ 2	
1-3 days a week	☐ 3	
About once a fortnight	☐ 4	
About once a month	☐ 5	
Less than once a month	☐ 6	
Never	☐ 7	➡ ***Go to E1***

A2 About how many miles did you drive in a car or van in the last 6 months? (If you are not certain, please give as good an estimate as you can) ***(Write in)***

____________________ miles

A3 About how many times did you drive more than 100 miles in a single day in the last 6 months? ***(Write in)***

____________________ times (PLEASE answer ‘0’ if you did not do this)

For office use only

A4 In the last 6 months, how often did you drive…. *(Tick ONE box per line)*

	Never (1)	Less than once a month (2)	About once a fortnight (3)	1-3 days a week (4)	4-6 days a week (5)	Every day (6)
**(a)** In a busy town or city centre	☐	☐	☐	☐	☐	☐
**(b)** In quiet parts of towns or cities	☐	☐	☐	☐	☐	☐
**(c)** On country roads	☐	☐	☐	☐	☐	☐
**(d)** On fast dual carriageways	☐	☐	☐	☐	☐	☐
**(e)** On motorways	☐	☐	☐	☐	☐	☐
**(f)** In the dark	☐	☐	☐	☐	☐	☐
**(g)** In the rain	☐	☐	☐	☐	☐	☐
(**h**) In fog	☐	☐	☐	☐	☐	☐
**(i)** In snow or ice	☐	☐	☐	☐	☐	☐
**(j**) To and from your place of work or study	☐	☐	☐	☐	☐	☐
**(k)** On your employer's business	☐	☐	☐	☐	☐	☐

A5 Who owned the vehicle you drove most often in the last 6 months? *(Tick ONE box only)*

- You personally ☐ 1
- Your employer (including cars leased to your employer) ☐ 2
- A member of your family ☐ 3
- A friend ☐ 4
- Some other person/organisation ☐ 5

A6 What further driver training have you had in the last 6 months? (*Tick ALL that apply*)

- None ☐ 1 ➡ ***Go to B1***
- Pass Plus ☐ 2
- Motorway lessons ☐ 3
- Driver training for company car drivers ☐ 4
- Other advanced driver training (e.g IAM, RoSPA, Diamond) ☐ 5
- Other (please specify) ______________________ ☐ 6

A7 Who paid for you to have this training? *(Tick ALL that apply)*

- Yourself ☐ 1
- A member of your family ☐ 2
- Your employer ☐ 3
- Someone else ☐ 4
- It was free ☐ 5

For office use only

SECTION B: ACCIDENTS

NEAR MISSES

B1 Many drivers have had the impression of only just avoiding an accident. How many times has this happened to you in the last 6 months? ***(Tick ONE box only)***

Never	☐	1
1 or 2 times	☐	2
3 to 5 times	☐	3
6 to 10 times	☐	4
More than 10 times	☐	5

ACCIDENTS

B2 How many accidents were you actually involved in when driving a car or van in the last 6 months? (Please include all accidents, regardless of how they were caused, how slight they were or where they happened) ***(Tick ONE box only)***

None	☐	1	➨ ***Go to C1***
One	☐	2	
Two	☐	3	
Three	☐	4	
More than three	☐	5	

Please now give us further details of your accident(s)

B3 When did the accident(s) happen? ***(Please enter time, month and year below)***

Most recent accident:	Time of day: ______ am/pm	Month: ____________	Year: ______
Next most recent:	Time of day: ______ am/pm	Month: ____________	Year: ______
One before that:	Time of day: ______ am/pm	Month: ____________	Year: ______

B4a Did the accident(s) happen ***(Tick ONE box for each accident)***

	Most recent accident	Next most recent	One before that
on a public road?	☐	☐	☐
in a car park, service area or petrol station?	☐	☐	☐
on a private driveway?	☐	☐	☐
on a private road?	☐	☐	☐
somewhere else? *(please specify)* ____________	☐	☐	☐

B4b Would you describe the accident(s) as: a 'low speed manoeuvring accident'? ***(Tick ONE box for each accident)***

Yes	☐	☐	☐
No	☐	☐	☐

B4c Would you describe the accident(s) as: a 'minor bump or scrape'? ***(Tick ONE box for each accident)***

Yes	☐	☐	☐
No	☐	☐	☐

For office use only

We are interested in the type(s) of accident(s) you were involved in while driving a car or van in the last 6 months.

B5 Apart from your vehicle, what else was involved in the accident(s)?

(Tick ALL boxes that apply for each accident)

	Most recent accident	Next most recent	One before that
Nothing/no-one	☐ 1	☐ 1	☐ 1
Other car(s) or van(s)	☐ 2	☐ 2	☐ 2
Motorbike(s)	☐ 3	☐ 3	☐ 3
HGV(s)	☐ 4	☐ 4	☐ 4
Bus(es)	☐ 5	☐ 5	☐ 5
Pedal cyclist(s)	☐ 6	☐ 6	☐ 6
Pedestrian(s)	☐ 7	☐ 7	☐ 7
Roadside objects	☐ 8	☐ 8	☐ 8
Other *(please specify)* ______	☐ 9	☐ 9	☐ 9

B6 What injuries were there to yourself or any others as a result of the accident(s)?

(Tick ONE box for each accident)

None	☐	☐	☐
Slight (e.g. cuts and bruises)	☐	☐	☐
Serious (needing hospital care)	☐	☐	☐
Someone was killed	☐	☐	☐

B7 To what extent do you think you were to blame for the accident(s)?

(Tick ONE box for each accident)

Not at all	☐	☐	☐
A little	☐	☐	☐
Quite a lot	☐	☐	☐
Entirely	☐	☐	☐

B8 What was the weather like at the time of the accident(s)?

(Tick ALL boxes that apply for each accident)

Dry	☐	☐	☐
Raining	☐	☐	☐
Fog	☐	☐	☐
Snow or ice on the road	☐	☐	☐

B9 Did the accident(s) happen

(Tick ONE box for each accident)

in daylight?	☐	☐	☐
at dawn or dusk?	☐	☐	☐
in the dark?	☐	☐	☐

For office use only

B10 Did the accident(s) happen *(Tick ONE box for each accident)*

	Most recent accident	Next most recent	One before that
in a town or city centre?	☐	☐	☐
on country roads?	☐	☐	☐
on fast dual carriageways?	☐	☐	☐
on motorways?	☐	☐	☐
somewhere else? *(please specify)* ________	☐	☐	☐

B11 What was the purpose of your journey when you had the accident(s)? *(Tick ONE box for each accident)*

Travelling to or from your place of work or study	☐	☐	☐
Travelling as part of your work	☐	☐	☐
Travelling for personal reasons	☐	☐	☐
Travelling for another reason *(please specify)* ________	☐	☐	☐

B12 How busy were the traffic conditions when the accident(s) happened? *(Tick ONE box for each accident)*

Very busy	☐	☐	☐
Busy	☐	☐	☐
Not very busy	☐	☐	☐
Not at all busy	☐	☐	☐

B13 What happened first in the accident(s)? *(Tick ONE box for each accident)*

Another vehicle hit your vehicle while it was parked	☐ 1	☐ 1	☐ 1
Your vehicle hit a pedestrian	☐ 2	☐ 2	☐ 2
Your vehicle hit a cyclist	☐ 3	☐ 3	☐ 3
Your vehicle hit the rear of another vehicle	☐ 4	☐ 4	☐ 4
Your vehicle hit the side of another vehicle	☐ 5	☐ 5	☐ 5
Another vehicle hit the rear of your vehicle	☐ 6	☐ 6	☐ 6
Another vehicle hit the side of your vehicle	☐ 7	☐ 7	☐ 7
Your vehicle was hit by an on-coming vehicle in *your* lane	☐ 8	☐ 8	☐ 8
Your vehicle was hit by an on-coming vehicle in *their* lane	☐ 9	☐ 9	☐ 9
Your vehicle hit a roadside object	☐ 10	☐ 10	☐ 10
Your vehicle left the road without hitting any other object	☐ 11	☐ 11	☐ 11
Other *(please specify)* ________	☐ 12	☐ 12	☐ 12

For office use only

B14a **Apart from yourself were there any other people in your vehicle at the time of the accident(s)?**

(Tick ONE box for each accident)

	Most recent accident	Next most recent	One before that
Yes	☐	☐	☐
No	☐	☐	☐

B14b **If there were other people in your vehicle how many were MALE?**

(Write in for each accident)

________ ________ ________

B14c **If there were other people in your vehicle how many were FEMALE?**

(Write in for each accident)

________ ________ ________

B14d **If there were other people in your vehicle at the time of the accident(s) what was the age of the oldest person?**

(Write in for each accident)

________ ________ ________

SECTION C: DRIVING OFFENCES

C1 **Many drivers are warned by the Police for motoring offences without any other action being taken.**

Apart from parking offences, has this happened to you in the last 6 months?

Yes ☐ No ☐ If *YES*, how many times? ____________________

C2 **Have you received any fixed penalty notices or summonses for motoring offences, apart from parking offences, in the last 6 months?**

Yes ☐ No ☐ If *YES*, how many times? ____________________

C3 **Are you waiting for a summons, or waiting to hear whether you might be issued with a summons, for a motoring offence in the last 6 months?**

Yes ☐ No ☐ If *YES*, how many times? ____________________

C4 **Have you been banned from driving during the last 6 months?**

Yes ☐ No ☐

C5 **Have you been told that you must go back to being a learner because you have too many penalty points on your licence in the last 6 months?**

Yes ☐ No ☐

For office use only

SECTION D: YOU AS A DRIVER

D1 Compared with other drivers of your age and sex:

(a) How likely are you to be being involved in an accident when driving a car or van? *(Tick ONE box only)*

- More likely than others ☐
- As likely as others ☐
- Less likely than others ☐

(b) How skilled a driver are you?

- More skilful than others ☐
- As skilful as others ☐
- Less skilful than others ☐

(c) How cautious a driver are you?

- More cautious than others ☐
- As cautious as others ☐
- Less cautious than others ☐

(d) How likely are you to avoid risky driving situations?

- More likely than others ☐
- As likely as others ☐
- Less likely than others ☐

(e) How fast do you drive?

- A little faster than others ☐
- About the same speed as others ☐
- A little slower than others ☐
- Much slower than others ☐

D2 How do you compare your driving to that of other drivers generally? *(Tick ONE box only)*

- Much better than average ☐
- A bit better than average ☐
- About average ☐
- A bit worse than average ☐
- Much worse than average ☐

D3 In general, how confident are you in your driving ability? *(Tick ONE box only)*

- Very confident ☐
- Fairly confident ☐
- Not very confident ☐
- Not at all confident ☐

For office use only

D4 How much do you think you need to improve your ability on each of the following driving skills? ***(Tick ONE box per line)***

	No improvement needed (1)	Some improvement needed (2)	A lot of improvement needed (3)
**(a)** Use of car controls	☐	☐	☐
**(b)** Pulling out of junctions	☐	☐	☐
**(c)** Reversing	☐	☐	☐
**(d)** Parking	☐	☐	☐
**(e)** Judging the speed of other traffic	☐	☐	☐
**(f)** Judging what other drivers are going to do	☐	☐	☐
**(g)** Spotting hazards	☐	☐	☐
**(h)** Driving in heavy traffic	☐	☐	☐
**(i)** Driving in the dark	☐	☐	☐
**(j)** Overtaking	☐	☐	☐
**(k)** Using roundabouts	☐	☐	☐
**(l)** Joining with moving traffic on a motorway or fast dual carriageway	☐	☐	☐
**(m)** Changing lanes on a motorway or fast dual carriageway	☐	☐	☐
**(n)** Driving on high speed roads	☐	☐	☐
**(o)** Driving on country roads	☐	☐	☐
**(p)** Driving in heavy rain	☐	☐	☐
**(q)** Driving in thick fog	☐	☐	☐
**(r)** Driving on snow or ice	☐	☐	☐
**(s)** Turning right	☐	☐	☐
**(t)** Knowing what speed is safe	☐	☐	☐

D5 How often do you exceed speed limits? ***(Tick ONE box only)***

Never	☐	1
Rarely	☐	2
Occasionally	☐	3
Fairly often	☐	4
Very often	☐	5
Always	☐	6

For office use only

D6 When driving, how often do you do each of the following?

(*Tick ONE box on each line*)

	Never (1)	Hardly ever (2)	Occas-ionally (3)	Quite often (4)	Frequ-ently (5)	Nearly all the time (6)
**(a)** Attempt to drive away from traffic lights in too high a gear						
**(b)** Overtake a slow driver on the inside						
**(c)** Have to confirm you are in the right gear						
**(d)** Attempt to overtake someone you hadn't noticed to be signalling a right turn						
**(e)** Forget where you left your car in a car park						
**(f)** Sound your horn to indicate your annoyance with another road user						
**(g)** Switch on one thing, such as the headlights, when you meant to switch on something else, such as the wipers						
**(h)** Change into the wrong gear when driving along						
**(i)** Pull out of a junction so far that the driver with the right of way has to stop and let you out						
**(j)** Use P Plates or Green L Plates to warn other road users that you are a new driver						

D7 And how often do you do each of these?

(*Tick ONE box on each line*)

	Never (1)	Hardly ever (2)	Occas-ionally (3)	Quite often (4)	Frequ-ently (5)	Nearly all the time (6)
**(a)** Realise you have no clear recollection of the road along which you have just been travelling						
**(b)** Cross a junction knowing that the traffic lights have already turned against you						
**(c)** Fail to notice that pedestrians are crossing when turning into a side street from a main road						
**(d)** Become angered by another driver and give chase with the intention of giving him/her a piece of your mind						
**(e)** Misread signs and take the wrong turning off a roundabout						
**(f)** Drive in either too high or too low a gear for the conditions						
**(g)** Disregard the speed limit on a residential road						
**(h)** On turning left, nearly hit a cyclist who has come up on your inside						
**(I)** Use a mobile phone without a hands free kit						

For office use only

D8 And how often do you do each of these? (*Tick ONE box on each line*)

	Never (1)	Hardly ever (2)	Occas-ionally (3)	Quite often (4)	Frequ-ently (5)	Nearly all the time (6)
**(a)** Stay in a motorway lane that you know will be closed before forcing your way in at the last minute						
**(b)** Queuing to turn left onto a main road, you pay such close attention to the main stream of traffic that you nearly hit the car in front						
**(c)** Drive when you suspect you may be over the legal alcohol limit						
**(d)** Forget to take the handbrake off before moving off						
**(e)** Become angered by a particular type of driver, and indicate your hostility by whatever means you can						
**(f)** Underestimate the speed of an oncoming vehicle when overtaking						
**(g)** Hit something when reversing that you had not previously seen						
**(h)** Race away from traffic lights with the intention of beating the driver next to you						
**(i)** Use a hands free mobile phone						

D9 And how often do you do each of these? (*Tick ONE box on each line*)

	Never (1)	Hardly ever (2)	Occas-ionally (3)	Quite often (4)	Frequ-ently (5)	Nearly all the time (6)
**(a)** Select the wrong gear when wanting to go into reverse						
**(b)** Intending to drive to destination A, you suddenly notice that you are on the road to destination B, perhaps because the latter is your more usual destination						
**(c)** Get into the wrong lane when approaching a roundabout or junction						
**(d)** Drive so close to the car in front that it would be difficult to stop in an emergency						
**(e)** Forget that the headlights are on full beam						
**(f)** Miss *Give Way* signs and narrowly avoid colliding with traffic having the right of way						
**(g)** Disregard the speed limit on a motorway						
**(h)** Fail to check your rear-view mirror before pulling out, changing lanes, etc						
**(i)** Brake too quickly on a slippery road, or steer the wrong way into a skid						
**(j)** Drive after taking drugs when you think you might still be affected by them						

For office use only

D10 When driving, how often do each of the following things happen to you?

(*Tick ONE box on each line*)

	Never (1)	Very rarely (2)	Occas-ionally (3)	Fairly often (4)	Very often (5)	Nearly all the time (6)
**(a)** You have to brake sharply to avoid a collision with the vehicle ahead of you because it has slowed						
**(b)** You pull out to overtake or turn right not noticing another vehicle in your 'blind spot'						
**(c)** You fail to notice someone waiting at a pedestrian crossing						
**(d)** You misjudge the gaps in main road traffic when pulling out of a side road						
**(e)** When cornering, you find you are travelling too fast to negotiate the bend safely and have to brake						
**(f)** You fail to give way when entering a roundabout to a vehicle already on the roundabout						
**(g)** You have to brake or swerve suddenly to avoid an accident						

D11 Please show what kind of driver you think you are by putting a tick somewhere on each of the lines below.

At either end of each line there is a word that describes a way of driving, and these words are opposites. Put your tick nearer to the word that best describes your driving. The closer your tick is to the word, the more you agree with this description of the way you drive.

	(1)	(2)	(3)	(4)	(5)	(6)	(7)	
Attentive								Inattentive
Careful								Careless
Decisive								Indecisive
Experienced								Inexperienced
Irritable								Placid
Nervous								Confident
Patient								Impatient
Responsible								Irresponsible
Safe								Risky
Selfish								Considerate
Slow								Fast
Tolerant								Intolerant

For office use only

SECTION E: YOUR DETAILS

E1 Please tell us when you passed your practical driving test

Month: ________________________ **Year:** ______________

For the success of this important research, we would like to get in touch with you again to see how your driving is going. Please fill in as many of the contact details below as possible. All information will be treated in the **strictest confidence** and will not be used for any purpose other than to get in touch with you in relation to this research.

E2 Home phone number: ________________________________

E3 Work phone number: ________________________________

E4 Mobile phone number: ________________________________

E5 Email address: ________________________________

If the address that this questionnaire was sent to is incorrect, or if there is a better address to help us contact you in the future, please provide the new address in the space below.

E6 New address: ____

____

____

____

____

Postcode: ______________________

E7 If you have any further comments to make about this survey and the issues raised, please write in the space provided:

E8 Please now return this questionnaire in the pre-paid envelope to:

NFER, The Mere, Upton Park, Slough, Berkshire, SL1 2DQ

Please return this questionnaire even if you have only answered the first few questions, as this information is very important to us.

Thank you very much for your help.

DRIVING EXPERIENCE QUESTIONNAIRE

This questionnaire asks you about your driving **in the last 12 months**. Please answer the questions by ticking the appropriate boxes and writing in the spaces as required. Please read carefully the instructions telling you which questions you should answer next. It may be that only a few of the questions apply to you. This questionnaire will only take a few minutes to complete and any information you provide will be treated in the **strictest confidence** and will be used for statistical purposes only.

SECTION A: YOUR DRIVING

A1 On average, how often did you drive in the last 12 months?

(Tick ONE box only)

- Every day ☐ 1
- 4-6 days a week ☐ 2
- 1-3 days a week ☐ 3
- About once a fortnight ☐ 4
- About once a month ☐ 5
- Less than once a month ☐ 6
- Never ☐ 7 ➡ *Go to E1*

A2 About how many miles did you drive in a car or van in the last 12 months? (If you are not certain, please give as good an estimate as you can) ***(Write in)***

_______________ miles

A3 About how many times did you drive more than 100 miles in a single day in the last 12 months? ***(Write in)***

_______________ times (PLEASE answer '0' if you did not do this)

For office use only

A4 In the last 12 months, how often did you drive…. *(Tick ONE box per line)*

	Never (1)	Less than once a month (2)	About once a fortnight (3)	1-3 days a week (4)	4-6 days a week (5)	Every day (6)
**(a)** In a busy town or city centre						
**(b)** In quiet parts of towns or cities						
**(c)** On country roads						
**(d)** On fast dual carriageways						
**(e)** On motorways						
**(f)** In the dark						
**(g)** In the rain						
**(h)** In fog						
**(i)** In snow or ice						
**(j)** To and from your place of work or study						
**(k)** On your employer's business						

A5 Who owned the vehicle you drove most often in the last 12 months? *(Tick ONE box only)*

- You personally ☐ 1
- Your employer (including cars leased to your employer) ☐ 2
- A member of your family ☐ 3
- A friend ☐ 4
- Some other person/organisation ☐ 5

A6 What further driver training have you had in the last 12 months? *(Tick ALL that apply)*

- None ☐ 1 ➡ ***Go to B1***
- Pass Plus ☐ 2
- Motorway lessons ☐ 3
- Driver training for company car drivers ☐ 4
- Other advanced driver training (e.g IAM, RoSPA, Diamond) ☐ 5
- Other (please specify) ______________________ ☐ 6

A7 Who paid for you to have this training? *(Tick ALL that apply)*

- Yourself ☐ 1
- A member of your family ☐ 2
- Your employer ☐ 3
- Someone else ☐ 4
- It was free ☐ 5

For office use only

SECTION B: ACCIDENTS

NEAR MISSES

B1 Many drivers have had the impression of only just avoiding an accident. How many times has this happened to you in the last 12 months? ***(Tick ONE box only)***

Never	☐	1
1 or 2 times	☐	2
3 to 5 times	☐	3
6 to 10 times	☐	4
More than 10 times	☐	5

ACCIDENTS

B2 How many accidents were you actually involved in when driving a car or van in the last 12 months? (Please include all accidents, regardless of how they were caused, how slight they were or where they happened) ***(Tick ONE box only)***

None	☐	1	➡ ***Go to C1***
One	☐	2	
Two	☐	3	
Three	☐	4	
More than three	☐	5	

Please now give us further details of your accident(s)

B3 When did the accident(s) happen? ***(Please enter time, month and year below)***

Most recent accident: Time of day: ______ am/pm Month: ______________ Year: _______

Next most recent: Time of day: ______ am/pm Month: ______________ Year: _______

One before that: Time of day: ______ am/pm Month: ______________ Year: _______

B4a Did the accident(s) happen ***(Tick ONE box for each accident)***

	Most recent accident	Next most recent	One before that
on a public road?	☐	☐	☐
in a car park, service area or petrol station?	☐	☐	☐
on a private driveway?	☐	☐	☐
on a private road?	☐	☐	☐
somewhere else? *(please specify)* ______________	☐	☐	☐

B4b Would you describe the accident(s) as: a 'low speed manoeuvring accident'? ***(Tick ONE box for each accident)***

Yes	☐	☐	☐
No	☐	☐	☐

B4c Would you describe the accident(s) as: a 'minor bump or scrape'? ***(Tick ONE box for each accident)***

Yes	☐	☐	☐
No	☐	☐	☐

For office use only

We are interested in the type(s) of accident(s) you were involved in while driving a car or van in the last 12 months.

B5 Apart from your vehicle, what else was involved in the accident(s)?

(Tick ALL boxes that apply for each accident)

	Most recent accident	Next most recent	One before that
Nothing/no-one	☐ 1	☐ 1	☐ 1
Other car(s) or van(s)	☐ 2	☐ 2	☐ 2
Motorbike(s)	☐ 3	☐ 3	☐ 3
HGV(s)	☐ 4	☐ 4	☐ 4
Bus(es)	☐ 5	☐ 5	☐ 5
Pedal cyclist(s)	☐ 6	☐ 6	☐ 6
Pedestrian(s)	☐ 7	☐ 7	☐ 7
Roadside objects	☐ 8	☐ 8	☐ 8
Other *(please specify)* ____________________	☐ 9	☐ 9	☐ 9

B6 What injuries were there to yourself or any others as a result of the accident(s)?

(Tick ONE box for each accident)

None	☐	☐	☐
Slight (e.g. cuts and bruises)	☐	☐	☐
Serious (needing hospital care)	☐	☐	☐
Someone was killed	☐	☐	☐

B7 To what extent do you think you were to blame for the accident(s)?

(Tick ONE box for each accident)

Not at all	☐	☐	☐
A little	☐	☐	☐
Quite a lot	☐	☐	☐
Entirely	☐	☐	☐

B8 What was the weather like at the time of the accident(s)?

(Tick ALL boxes that apply for each accident)

Dry	☐	☐	☐
Raining	☐	☐	☐
Fog	☐	☐	☐
Snow or ice on the road	☐	☐	☐

B9 Did the accident(s) happen

(Tick ONE box for each accident)

in daylight?	☐	☐	☐
at dawn or dusk?	☐	☐	☐
in the dark?	☐	☐	☐

For office use only

B10 Did the accident(s) happen

(Tick ONE box for each accident)

	Most recent accident	Next most recent	One before that
in a town or city centre?			
on country roads?			
on fast dual carriageways?			
on motorways?			
somewhere else? *(please specify)* ____________			

B11 What was the purpose of your journey when you had the accident(s)?

(Tick ONE box for each accident)

Travelling to or from your place of work or study			
Travelling as part of your work			
Travelling for personal reasons			
Travelling for another reason *(please specify)* ____________			

B12 How busy were the traffic conditions when the accident(s) happened?

(Tick ONE box for each accident)

Very busy			
Busy			
Not very busy			
Not at all busy			

B13 What happened first in the accident(s)?

(Tick ONE box for each accident)

Another vehicle hit your vehicle while it was parked	1	1	1
Your vehicle hit a pedestrian	2	2	2
Your vehicle hit a cyclist	3	3	3
Your vehicle hit the rear of another vehicle	4	4	4
Your vehicle hit the side of another vehicle	5	5	5
Another vehicle hit the rear of your vehicle	6	6	6
Another vehicle hit the side of your vehicle	7	7	7
Your vehicle was hit by an on-coming vehicle in *your* lane	8	8	8
Your vehicle was hit by an on-coming vehicle in *their* lane	9	9	9
Your vehicle hit a roadside object	10	10	10
Your vehicle left the road without hitting any other object	11	11	11
Other (*please specify*) ____________	12	12	12

For office use only

B14a **Apart from yourself were there any other people in your vehicle at the time of the accident(s)?**

(Tick ONE box for each accident)

	Most recent accident	Next most recent	One before that
Yes	☐	☐	☐
No	☐	☐	☐

B14b **If there were other people in your vehicle how many were MALE?**

(Write in for each accident)

________ ________ ________

B14c **If there were other people in your vehicle how many were FEMALE?**

(Write in for each accident)

________ ________ ________

B14d **If there were other people in your vehicle at the time of the accident(s) what was the age of the oldest person?**

(Write in for each accident)

________ ________ ________

SECTION C: DRIVING OFFENCES

C1 **Many drivers are warned by the Police for motoring offences without any other action being taken.**
Apart from parking offences, has this happened to you in the last 12 months?

Yes ☐ No ☐ If *YES*, how many times? ____________

C2 **Have you received any fixed penalty notices or summonses for motoring offences, apart from parking offences, in the last 12 months?**

Yes ☐ No ☐ If *YES*, how many times? ____________

C3 **Are you waiting for a summons, or waiting to hear whether you might be issued with a summons, for a motoring offence in the last 12 months?**

Yes ☐ No ☐ If *YES*, how many times? ____________

C4 **Have you been banned from driving during the last 12 months?**

Yes ☐ No ☐

C5 **Have you been told that you must go back to being a learner because you have too many penalty points on your licence in the last 12 months?**

Yes ☐ No ☐

For office use only

SECTION D: YOU AS A DRIVER

D1 Compared with other drivers of your age and sex:

(a) How likely are you to be being involved in an accident when driving a car or van? *(Tick ONE box only)*

- More likely than others ☐
- As likely as others ☐
- Less likely than others ☐

(b) How skilled a driver are you?

- More skilful than others ☐
- As skilful as others ☐
- Less skilful than others ☐

(c) How cautious a driver are you?

- More cautious than others ☐
- As cautious as others ☐
- Less cautious than others ☐

(d) How likely are you to avoid risky driving situations?

- More likely than others ☐
- As likely as others ☐
- Less likely than others ☐

(e) How fast do you drive?

- A little faster than others ☐
- About the same speed as others ☐
- A little slower than others ☐
- Much slower than others ☐

D2 How do you compare your driving to that of other drivers generally? *(Tick ONE box only)*

- Much better than average ☐
- A bit better than average ☐
- About average ☐
- A bit worse than average ☐
- Much worse than average ☐

D3 In general, how confident are you in your driving ability? *(Tick ONE box only)*

- Very confident ☐
- Fairly confident ☐
- Not very confident ☐
- Not at all confident ☐

For office use only

D4 How much do you think you need to improve your ability on each of the following driving skills? *(Tick ONE box per line)*

	No improvement needed (1)	Some improvement needed (2)	A lot of improvement needed (3)
**(a)** Use of car controls			
**(b)** Pulling out of junctions			
**(c)** Reversing			
**(d)** Parking			
**(e)** Judging the speed of other traffic			
**(f)** Judging what other drivers are going to do			
**(g)** Spotting hazards			
**(h)** Driving in heavy traffic			
**(i)** Driving in the dark			
**(j)** Overtaking			
**(k)** Using roundabouts			
**(l)** Joining with moving traffic on a motorway or fast dual carriageway			
**(m)** Changing lanes on a motorway or fast dual carriageway			
**(n)** Driving on high speed roads			
**(o)** Driving on country roads			
**(p)** Driving in heavy rain			
**(q)** Driving in thick fog			
**(r)** Driving on snow or ice			
**(s)** Turning right			
**(t)** Knowing what speed is safe			

D5 How often do you exceed speed limits? *(Tick ONE box only)*

Never	☐	1
Rarely	☐	2
Occasionally	☐	3
Fairly often	☐	4
Very often	☐	5
Always	☐	6

For office use only

D6 When driving, how often do you do each of the following?

(*Tick ONE box on each line*)

	Never (1)	Hardly ever (2)	Occas-ionally (3)	Quite often (4)	Frequ-ently (5)	Nearly all the time (6)
**(a)** Attempt to drive away from traffic lights in too high a gear						
**(b)** Overtake a slow driver on the inside						
**(c)** Have to confirm you are in the right gear						
**(d)** Attempt to overtake someone you hadn't noticed to be signalling a right turn						
**(e)** Forget where you left your car in a car park						
**(f)** Sound your horn to indicate your annoyance with another road user						
**(g)** Switch on one thing, such as the headlights, when you meant to switch on something else, such as the wipers						
**(h)** Change into the wrong gear when driving along						
**(i)** Pull out of a junction so far that the driver with the right of way has to stop and let you out						
**(j)** Use P Plates or Green L Plates to warn other road users that you are a new driver						

D7 And how often do you do each of these?

(*Tick ONE box on each line*)

	Never (1)	Hardly ever (2)	Occas-ionally (3)	Quite often (4)	Frequ-ently (5)	Nearly all the time (6)
**(a)** Realise you have no clear recollection of the road along which you have just been travelling						
**(b)** Cross a junction knowing that the traffic lights have already turned against you						
**(c)** Fail to notice that pedestrians are crossing when turning into a side street from a main road						
**(d)** Become angered by another driver and give chase with the intention of giving him/her a piece of your mind						
**(e)** Misread signs and take the wrong turning off a roundabout						
**(f)** Drive in either too high or too low a gear for the conditions						
**(g)** Disregard the speed limit on a residential road						
**(h)** On turning left, nearly hit a cyclist who has come up on your inside						
**(I)** Use a mobile phone without a hands free kit						

For office use only

D8 And how often do you do each of these? (*Tick ONE box on each line*)

	Never (1)	Hardly ever (2)	Occasionally (3)	Quite often (4)	Frequently (5)	Nearly all the time (6)
**(a)** Stay in a motorway lane that you know will be closed before forcing your way in at the last minute						
**(b)** Queuing to turn left onto a main road, you pay such close attention to the main stream of traffic that you nearly hit the car in front						
**(c)** Drive when you suspect you may be over the legal alcohol limit						
**(d)** Forget to take the handbrake off before moving off						
**(e)** Become angered by a particular type of driver, and indicate your hostility by whatever means you can						
**(f)** Underestimate the speed of an oncoming vehicle when overtaking						
**(g)** Hit something when reversing that you had not previously seen						
**(h)** Race away from traffic lights with the intention of beating the driver next to you						
**(i)** Use a hands free mobile phone						

D9 And how often do you do each of these? (*Tick ONE box on each line*)

	Never (1)	Hardly ever (2)	Occasionally (3)	Quite often (4)	Frequently (5)	Nearly all the time (6)
**(a)** Select the wrong gear when wanting to go into reverse						
**(b)** Intending to drive to destination A, you suddenly notice that you are on the road to destination B, perhaps because the latter is your more usual destination						
**(c)** Get into the wrong lane when approaching a roundabout or junction						
**(d)** Drive so close to the car in front that it would be difficult to stop in an emergency						
**(e)** Forget that the headlights are on full beam						
**(f)** Miss *Give Way* signs and narrowly avoid colliding with traffic having the right of way						
**(g)** Disregard the speed limit on a motorway						
**(h)** Fail to check your rear-view mirror before pulling out, changing lanes, etc						
**(i)** Brake too quickly on a slippery road, or steer the wrong way into a skid						
**(j)** Drive after taking drugs when you think you might still be affected by them						

D10 When driving, how often do each of the following things happen to you?

(*Tick ONE box on each line*)

	Never (1)	Very rarely (2)	Occas-ionally (3)	Fairly often (4)	Very often (5)	Nearly all the time (6)
**(a)** You have to brake sharply to avoid a collision with the vehicle ahead of you because it has slowed						
**(b)** You pull out to overtake or turn right not noticing another vehicle in your 'blind spot'						
**(c)** You fail to notice someone waiting at a pedestrian crossing						
**(d)** You misjudge the gaps in main road traffic when pulling out of a side road						
**(e)** When cornering, you find you are travelling too fast to negotiate the bend safely and have to brake						
**(f)** You fail to give way when entering a roundabout to a vehicle already on the roundabout						
**(g)** You have to brake or swerve suddenly to avoid an accident						

D11 Please show what kind of driver you think you are by putting a tick somewhere on each of the lines below.

At either end of each line there is a word that describes a way of driving, and these words are opposites. Put your tick nearer to the word that best describes your driving. The closer your tick is to the word, the more you agree with this description of the way you drive.

	(1)	(2)	(3)	(4)	(5)	(6)	(7)	
Attentive								Inattentive
Careful								Careless
Decisive								Indecisive
Experienced								Inexperienced
Irritable								Placid
Nervous								Confident
Patient								Impatient
Responsible								Irresponsible
Safe								Risky
Selfish								Considerate
Slow								Fast
Tolerant								Intolerant

For office use only

SECTION E: YOUR DETAILS

E1 Please tell us when you passed your practical driving test

Month: ______________________ **Year:** _____________

For the success of this important research, we would like to get in touch with you again to see how your driving is going. Please fill in as many of the contact details below as possible. All information will be treated in the **strictest confidence** and will not be used for any purpose other than to get in touch with you in relation to this research.

E2 Home phone number: _______________________________

E3 Work phone number: _______________________________

E4 Mobile phone number: _______________________________

E5 Email address: _______________________________

If the address that this questionnaire was sent to is incorrect, or if there is a better address to help us contact you in the future, please provide the new address in the space below.

E6 New address: ____

____

____

____

____

Postcode: ______________________

E7 If you have any further comments to make about this survey and the issues raised, please write in the space provided:

E8 Please now return this questionnaire in the pre-paid envelope to:

NFER, The Mere, Upton Park, Slough, Berkshire, SL1 2DQ

Please return this questionnaire even if you have only answered the first few questions, as this information is very important to us.

Thank you very much for your help.

DRIVING EXPERIENCE QUESTIONNAIRE

This questionnaire asks you about your driving **in the last 12 months**. Please answer the questions by ticking the appropriate boxes and writing in the spaces as required. Please read carefully the instructions telling you which questions you should answer next. It may be that only a few of the questions apply to you. This questionnaire will only take a few minutes to complete and any information you provide will be treated in the **strictest confidence** and will be used for statistical purposes only.

SECTION A: YOUR DRIVING

A1 On average, how often did you drive in the last 12 months?

(Tick ONE box only)

Every day	☐ 1	
4-6 days a week	☐ 2	
1-3 days a week	☐ 3	
About once a fortnight	☐ 4	
About once a month	☐ 5	
Less than once a month	☐ 6	
Never	☐ 7	➡ ***Go to E1***

A2 About how many miles did you drive in a car or van in the last 12 months? (If you are not certain, please give as good an estimate as you can) ***(Write in)***

________________________ miles

A3 About how many times did you drive more than 100 miles in a single day in the last 12 months? ***(Write in)***

________________________ times (PLEASE answer ‘0’ if you did not do this)

For office use only

A4 **In the last 12 months, how often did you drive….** *(Tick ONE box per line)*

	Never (1)	Less than once a month (2)	About once a fortnight (3)	1-3 days a week (4)	4-6 days a week (5)	Every day (6)
**(a)** In a busy town or city centre						
**(b)** In quiet parts of towns or cities						
**(c)** On country roads						
**(d)** On fast dual carriageways						
**(e)** On motorways						
**(f)** In the dark						
**(g)** In the rain						
**(h)** In fog						
**(i)** In snow or ice						
**(j)** To and from your place of work or study						
**(k)** On your employer's business						

A5 **Who owned the vehicle you drove most often in the last 12 months?** *(Tick ONE box only)*

- You personally ☐ 1
- Your employer (including cars leased to your employer) ☐ 2
- A member of your family ☐ 3
- A friend ☐ 4
- Some other person/organisation ☐ 5

A6 **What further driver training have you had in the last 12 months?** *(Tick ALL that apply)*

- None ☐ 1 ➡ ***Go to B1***
- Pass Plus ☐ 2
- Motorway lessons ☐ 3
- Driver training for company car drivers ☐ 4
- Other advanced driver training (e.g IAM, RoSPA, Diamond) ☐ 5
- Other (please specify) ______________________ ☐ 6

A7 **Who paid for you to have this training?** *(Tick ALL that apply)*

- Yourself ☐ 1
- A member of your family ☐ 2
- Your employer ☐ 3
- Someone else ☐ 4
- It was free ☐ 5

For office use only

SECTION B: ACCIDENTS

NEAR MISSES

B1 Many drivers have had the impression of only just avoiding an accident. How many times has this happened to you in the last 12 months? ***(Tick ONE box only)***

Never	☐	1
1 or 2 times	☐	2
3 to 5 times	☐	3
6 to 10 times	☐	4
More than 10 times	☐	5

ACCIDENTS

B2 How many accidents were you actually involved in when driving a car or van in the last 12 months? (Please include all accidents, regardless of how they were caused, how slight they were or where they happened) ***(Tick ONE box only)***

None	☐	1	➡ ***Go to C1***
One	☐	2	
Two	☐	3	
Three	☐	4	
More than three	☐	5	

Please now give us further details of your accident(s)

B3 When did the accident(s) happen? ***(Please enter time, month and year below)***

Most recent accident:	Time of day: ______ am/pm	Month: ____________	Year: ______
Next most recent:	Time of day: ______ am/pm	Month: ____________	Year: ______
One before that:	Time of day: ______ am/pm	Month: ____________	Year: ______

B4a Did the accident(s) happen ***(Tick ONE box for each accident)***

	Most recent accident	Next most recent	One before that
on a public road?	☐	☐	☐
in a car park, service area or petrol station?	☐	☐	☐
on a private driveway?	☐	☐	☐
on a private road?	☐	☐	☐
somewhere else? *(please specify)* ____________	☐	☐	☐

B4b Would you describe the accident(s) as: a 'low speed manoeuvring accident'? ***(Tick ONE box for each accident)***

Yes	☐	☐	☐
No	☐	☐	☐

B4c Would you describe the accident(s) as: a 'minor bump or scrape'? ***(Tick ONE box for each accident)***

Yes	☐	☐	☐
No	☐	☐	☐

For office use only

We are interested in the type(s) of accident(s) you were involved in while driving a car or van in the last 12 months.

B5 Apart from your vehicle, what else was involved in the accident(s)?

(Tick ALL boxes that apply for each accident)

	Most recent accident	Next most recent	One before that
Nothing/no-one	☐ 1	☐ 1	☐ 1
Other car(s) or van(s)	☐ 2	☐ 2	☐ 2
Motorbike(s)	☐ 3	☐ 3	☐ 3
HGV(s)	☐ 4	☐ 4	☐ 4
Bus(es)	☐ 5	☐ 5	☐ 5
Pedal cyclist(s)	☐ 6	☐ 6	☐ 6
Pedestrian(s)	☐ 7	☐ 7	☐ 7
Roadside objects	☐ 8	☐ 8	☐ 8
Other *(please specify)* ____________	☐ 9	☐ 9	☐ 9

B6 What injuries were there to yourself or any others as a result of the accident(s)?

(Tick ONE box for each accident)

None	☐	☐	☐
Slight (e.g. cuts and bruises)	☐	☐	☐
Serious (needing hospital care)	☐	☐	☐
Someone was killed	☐	☐	☐

B7 To what extent do you think you were to blame for the accident(s)?

(Tick ONE box for each accident)

Not at all	☐	☐	☐
A little	☐	☐	☐
Quite a lot	☐	☐	☐
Entirely	☐	☐	☐

B8 What was the weather like at the time of the accident(s)?

(Tick ALL boxes that apply for each accident)

Dry	☐	☐	☐
Raining	☐	☐	☐
Fog	☐	☐	☐
Snow or ice on the road	☐	☐	☐

B9 Did the accident(s) happen

(Tick ONE box for each accident)

in daylight?	☐	☐	☐
at dawn or dusk?	☐	☐	☐
in the dark?	☐	☐	☐

For office use only

B10 Did the accident(s) happen

(Tick ONE box for each accident)

	Most recent accident	Next most recent	One before that
in a town or city centre?	☐	☐	☐
on country roads?	☐	☐	☐
on fast dual carriageways?	☐	☐	☐
on motorways?	☐	☐	☐
somewhere else? *(please specify)* ________________	☐	☐	☐

B11 What was the purpose of your journey when you had the accident(s)?

(Tick ONE box for each accident)

Travelling to or from your place of work or study	☐	☐	☐
Travelling as part of your work	☐	☐	☐
Travelling for personal reasons	☐	☐	☐
Travelling for another reason *(please specify)* ________________	☐	☐	☐

B12 How busy were the traffic conditions when the accident(s) happened?

(Tick ONE box for each accident)

Very busy	☐	☐	☐
Busy	☐	☐	☐
Not very busy	☐	☐	☐
Not at all busy	☐	☐	☐

B13 What happened first in the accident(s)?

(Tick ONE box for each accident)

Another vehicle hit your vehicle while it was parked	☐ 1	☐ 1	☐ 1
Your vehicle hit a pedestrian	☐ 2	☐ 2	☐ 2
Your vehicle hit a cyclist	☐ 3	☐ 3	☐ 3
Your vehicle hit the rear of another vehicle	☐ 4	☐ 4	☐ 4
Your vehicle hit the side of another vehicle	☐ 5	☐ 5	☐ 5
Another vehicle hit the rear of your vehicle	☐ 6	☐ 6	☐ 6
Another vehicle hit the side of your vehicle	☐ 7	☐ 7	☐ 7
Your vehicle was hit by an on-coming vehicle in *your* lane	☐ 8	☐ 8	☐ 8
Your vehicle was hit by an on-coming vehicle in *their* lane	☐ 9	☐ 9	☐ 9
Your vehicle hit a roadside object	☐ 10	☐ 10	☐ 10
Your vehicle left the road without hitting any other object	☐ 11	☐ 11	☐ 11
Other (*please specify*) ________________	☐ 12	☐ 12	☐ 12

For office use only

B14a **Apart from yourself were there any other people in your vehicle at the time of the accident(s)?**

(Tick ONE box for each accident)

	Most recent accident	Next most recent	One before that
Yes	☐	☐	☐
No	☐	☐	☐

B14b **If there were other people in your vehicle how many were MALE?**

(Write in for each accident)

________ ________ ________

B14c **If there were other people in your vehicle how many were FEMALE?**

(Write in for each accident)

________ ________ ________

B14d **If there were other people in your vehicle at the time of the accident(s) what was the age of the oldest person?**

(Write in for each accident)

________ ________ ________

SECTION C: DRIVING OFFENCES

C1 **Many drivers are warned by the Police for motoring offences without any other action being taken.**

Apart from parking offences, has this happened to you in the last 12 months?

Yes ☐ No ☐ If *YES*, how many times? ____________________

C2 **Have you received any fixed penalty notices or summonses for motoring offences, apart from parking offences, in the last 12 months?**

Yes ☐ No ☐ If *YES*, how many times? ____________________

C3 **Are you waiting for a summons, or waiting to hear whether you might be issued with a summons, for a motoring offence in the last 12 months?**

Yes ☐ No ☐ If *YES*, how many times? ____________________

C4 **Have you been banned from driving during the last 12 months?**

Yes ☐ No ☐

C5 **Have you been told that you must go back to being a learner because you have too many penalty points on your licence in the last 12 months?**

Yes ☐ No ☐

For office use only

SECTION D: YOU AS A DRIVER

D1 Compared with other drivers of your age and sex:

(a) How likely are you to be being involved in an accident when driving a car or van? *(Tick ONE box only)*

- More likely than others ☐
- As likely as others ☐
- Less likely than others ☐

(b) How skilled a driver are you?

- More skilful than others ☐
- As skilful as others ☐
- Less skilful than others ☐

(c) How cautious a driver are you?

- More cautious than others ☐
- As cautious as others ☐
- Less cautious than others ☐

(d) How likely are you to avoid risky driving situations?

- More likely than others ☐
- As likely as others ☐
- Less likely than others ☐

(e) How fast do you drive?

- A little faster than others ☐
- About the same speed as others ☐
- A little slower than others ☐
- Much slower than others ☐

D2 How do you compare your driving to that of other drivers generally? *(Tick ONE box only)*

- Much better than average ☐
- A bit better than average ☐
- About average ☐
- A bit worse than average ☐
- Much worse than average ☐

D3 In general, how confident are you in your driving ability? *(Tick ONE box only)*

- Very confident ☐
- Fairly confident ☐
- Not very confident ☐
- Not at all confident ☐

For office use only

D4 How much do you think you need to improve your ability on each of the following driving skills? ***(Tick ONE box per line)***

	No improvement needed (1)	Some improvement needed (2)	A lot of improvement needed (3)
**(a)** Use of car controls			
**(b)** Pulling out of junctions			
**(c)** Reversing			
**(d)** Parking			
**(e)** Judging the speed of other traffic			
**(f)** Judging what other drivers are going to do			
**(g)** Spotting hazards			
**(h)** Driving in heavy traffic			
**(i)** Driving in the dark			
**(j)** Overtaking			
**(k)** Using roundabouts			
**(l)** Joining with moving traffic on a motorway or fast dual carriageway			
**(m)** Changing lanes on a motorway or fast dual carriageway			
**(n)** Driving on high speed roads			
**(o)** Driving on country roads			
**(p)** Driving in heavy rain			
**(q)** Driving in thick fog			
**(r)** Driving on snow or ice			
**(s)** Turning right			
**(t)** Knowing what speed is safe			

D5 How often do you exceed speed limits? ***(Tick ONE box only)***

Never	1
Rarely	2
Occasionally	3
Fairly often	4
Very often	5
Always	6

For office use only

D6 When driving, how often do you do each of the following?

(*Tick ONE box on each line*)

	Never (1)	Hardly ever (2)	Occas-ionally (3)	Quite often (4)	Frequ-ently (5)	Nearly all the time (6)
**(a)** Attempt to drive away from traffic lights in too high a gear						
**(b)** Overtake a slow driver on the inside						
**(c)** Have to confirm you are in the right gear						
**(d)** Attempt to overtake someone you hadn't noticed to be signalling a right turn						
**(e)** Forget where you left your car in a car park						
**(f)** Sound your horn to indicate your annoyance with another road user						
**(g)** Switch on one thing, such as the headlights, when you meant to switch on something else, such as the wipers						
**(h)** Change into the wrong gear when driving along						
**(i)** Pull out of a junction so far that the driver with the right of way has to stop and let you out						
**(j)** Use P Plates or Green L Plates to warn other road users that you are a new driver						

D7 And how often do you do each of these?

(*Tick ONE box on each line*)

	Never (1)	Hardly ever (2)	Occas-ionally (3)	Quite often (4)	Frequ-ently (5)	Nearly all the time (6)
**(a)** Realise you have no clear recollection of the road along which you have just been travelling						
**(b)** Cross a junction knowing that the traffic lights have already turned against you						
**(c)** Fail to notice that pedestrians are crossing when turning into a side street from a main road						
**(d)** Become angered by another driver and give chase with the intention of giving him/her a piece of your mind						
**(e)** Misread signs and take the wrong turning off a roundabout						
**(f)** Drive in either too high or too low a gear for the conditions						
**(g)** Disregard the speed limit on a residential road						
**(h)** On turning left, nearly hit a cyclist who has come up on your inside						
**(I)** Use a mobile phone without a hands free kit						

For office use only

D8 And how often do you do each of these? (*Tick ONE box on each line*)

	Never (1)	Hardly ever (2)	Occas-ionally (3)	Quite often (4)	Frequ-ently (5)	Nearly all the time (6)
**(a)** Stay in a motorway lane that you know will be closed before forcing your way in at the last minute						
**(b)** Queuing to turn left onto a main road, you pay such close attention to the main stream of traffic that you nearly hit the car in front						
**(c)** Drive when you suspect you may be over the legal alcohol limit						
**(d)** Forget to take the handbrake off before moving off						
**(e)** Become angered by a particular type of driver, and indicate your hostility by whatever means you can						
**(f)** Underestimate the speed of an oncoming vehicle when overtaking						
**(g)** Hit something when reversing that you had not previously seen						
**(h)** Race away from traffic lights with the intention of beating the driver next to you						
**(i)** Use a hands free mobile phone						

D9 And how often do you do each of these? (*Tick ONE box on each line*)

	Never (1)	Hardly ever (2)	Occas-ionally (3)	Quite often (4)	Frequ-ently (5)	Nearly all the time (6)
**(a)** Select the wrong gear when wanting to go into reverse						
**(b)** Intending to drive to destination A, you suddenly notice that you are on the road to destination B, perhaps because the latter is your more usual destination						
**(c)** Get into the wrong lane when approaching a roundabout or junction						
**(d)** Drive so close to the car in front that it would be difficult to stop in an emergency						
**(e)** Forget that the headlights are on full beam						
**(f)** Miss *Give Way* signs and narrowly avoid colliding with traffic having the right of way						
**(g)** Disregard the speed limit on a motorway						
**(h)** Fail to check your rear-view mirror before pulling out, changing lanes, etc						
**(i)** Brake too quickly on a slippery road, or steer the wrong way into a skid						
**(j)** Drive after taking drugs when you think you might still be affected by them						

For office use only

D10 When driving, how often do each of the following things happen to you?

(*Tick ONE box on each line*)

	Never (1)	Very rarely (2)	Occas-ionally (3)	Fairly often (4)	Very often (5)	Nearly all the time (6)
**(a)** You have to brake sharply to avoid a collision with the vehicle ahead of you because it has slowed						
**(b)** You pull out to overtake or turn right not noticing another vehicle in your 'blind spot'						
**(c)** You fail to notice someone waiting at a pedestrian crossing						
**(d)** You misjudge the gaps in main road traffic when pulling out of a side road						
**(e)** When cornering, you find you are travelling too fast to negotiate the bend safely and have to brake						
**(f)** You fail to give way when entering a roundabout to a vehicle already on the roundabout						
**(g)** You have to brake or swerve suddenly to avoid an accident						

D11 Please show what kind of driver you think you are by putting a tick somewhere on each of the lines below.

At either end of each line there is a word that describes a way of driving, and these words are opposites. Put your tick nearer to the word that best describes your driving. The closer your tick is to the word, the more you agree with this description of the way you drive.

	(1)	(2)	(3)	(4)	(5)	(6)	(7)	
Attentive								Inattentive
Careful								Careless
Decisive								Indecisive
Experienced								Inexperienced
Irritable								Placid
Nervous								Confident
Patient								Impatient
Responsible								Irresponsible
Safe								Risky
Selfish								Considerate
Slow								Fast
Tolerant								Intolerant

For office use only

SECTION E: YOUR DETAILS

E1 Please tell us when you passed your practical driving test

Month: ______________________ **Year:** _____________

For the success of this important research, we would like to get in touch with you again to see how your driving is going. Please fill in as many of the contact details below as possible. All information will be treated in the **strictest confidence** and will not be used for any purpose other than to get in touch with you in relation to this research.

E2 Home phone number: ______________________________

E3 Work phone number: ______________________________

E4 Mobile phone number: ______________________________

E5 Email address: ______________________________

If the address that this questionnaire was sent to is incorrect, or if there is a better address to help us contact you in the future, please provide the new address in the space below.

E6 New address: ____

____

____

____

____

Postcode: ______________________

E7 If you have any further comments to make about this survey and the issues raised, please write in the space provided:

E8 Please now return this questionnaire in the pre-paid envelope to:

NFER, The Mere, Upton Park, Slough, Berkshire, SL1 2DQ

Please return this questionnaire even if you have only answered the first few questions, as this information is very important to us.

Thank you very much for your help.
