# Supplementary material for: Network models of driver behavior
Source: PeerJ. 2019 Jan 10;6:e6119. doi: 10.7717/peerj.6119 (PMC6330205; doi:10.7717/peerj.6119)
Supplement: Supplemental Information 5 [file peerj-07-6119-s005.pdf]

Variable	v1	v2	v3	v4	v5	v6	v7	v8	v9	v10	v11	v12	v13	v14	v15	v16	v17	v18	v19	v20	v21	v22	v23	v24	v25	v26	v27	v28	v29	v30
v1	1.00	0.16	0.24	0.27	0.12	0.37	0.26	0.32	0.33	0.36	0.10	0.33	0.21	0.18	0.12	0.16	0.36	0.03	0.13	0.27	0.23	0.15	0.26	0.37	0.29	0.30	0.11	0.28	0.26	0.02
v2	0.16	1.00	0.27	0.11	0.39	0.08	0.27	0.17	0.37	0.19	0.37	0.08	0.40	0.16	0.30	0.41	0.28	0.18	0.35	0.23	0.12	0.42	0.13	0.10	0.31	0.16	0.41	0.20	0.25	0.12
v3	0.24	0.27	1.00	0.21	0.21	0.29	0.30	0.22	0.35	0.39	0.28	0.27	0.23	0.34	0.17	0.23	0.33	0.11	0.19	0.41	0.25	0.17	0.27	0.29	0.31	0.37	0.16	0.24	0.32	0.13
v4	0.27	0.11	0.21	1.00	0.20	0.26	0.19	0.36	0.26	0.28	0.11	0.34	0.15	0.25	0.15	0.13	0.30	0.06	0.22	0.22	0.18	0.12	0.42	0.34	0.20	0.25	0.11	0.17	0.21	0.06
v5	0.12	0.39	0.21	0.20	1.00	0.09	0.27	0.25	0.33	0.25	0.47	0.15	0.34	0.19	0.33	0.30	0.28	0.18	0.68	0.26	0.16	0.40	0.21	0.13	0.29	0.17	0.30	0.23	0.25	0.09
v6	0.37	0.08	0.29	0.26	0.09	1.00	0.29	0.26	0.26	0.36	0.06	0.37	0.20	0.20	0.10	0.12	0.34	0.04	0.16	0.32	0.23	0.07	0.36	0.43	0.28	0.30	0.10	0.30	0.27	0.10
v7	0.26	0.27	0.30	0.19	0.27	0.29	1.00	0.26	0.40	0.41	0.23	0.26	0.33	0.30	0.22	0.34	0.44	0.12	0.28	0.36	0.26	0.28	0.25	0.32	0.40	0.35	0.25	0.34	0.32	0.09
v8	0.32	0.17	0.22	0.36	0.25	0.26	0.26	1.00	0.39	0.37	0.11	0.32	0.34	0.22	0.25	0.20	0.38	0.13	0.27	0.31	0.19	0.27	0.45	0.38	0.37	0.27	0.30	0.34	0.28	0.15
v9	0.33	0.37	0.35	0.26	0.33	0.26	0.40	0.39	1.00	0.46	0.32	0.29	0.51	0.28	0.38	0.39	0.44	0.24	0.37	0.38	0.28	0.43	0.34	0.36	0.49	0.34	0.42	0.38	0.35	0.16
v10	0.36	0.19	0.39	0.28	0.25	0.36	0.41	0.37	0.46	1.00	0.24	0.40	0.36	0.40	0.24	0.29	0.49	0.15	0.28	0.43	0.31	0.29	0.36	0.44	0.49	0.49	0.26	0.45	0.41	0.15
v11	0.10	0.37	0.28	0.11	0.47	0.06	0.23	0.11	0.32	0.24	1.00	0.09	0.30	0.24	0.25	0.28	0.24	0.18	0.46	0.30	0.12	0.38	0.10	0.07	0.26	0.21	0.26	0.19	0.27	0.13
v12	0.33	0.08	0.27	0.34	0.15	0.37	0.26	0.32	0.29	0.40	0.09	1.00	0.22	0.26	0.16	0.20	0.39	0.09	0.20	0.36	0.22	0.16	0.44	0.61	0.30	0.33	0.17	0.24	0.30	0.05
v13	0.21	0.40	0.23	0.15	0.34	0.20	0.33	0.34	0.51	0.36	0.30	0.22	1.00	0.24	0.46	0.38	0.43	0.35	0.43	0.31	0.19	0.51	0.24	0.27	0.58	0.27	0.62	0.44	0.38	0.16
v14	0.18	0.16	0.34	0.25	0.19	0.20	0.30	0.22	0.28	0.40	0.24	0.26	0.24	1.00	0.16	0.29	0.43	0.16	0.25	0.31	0.27	0.20	0.24	0.25	0.32	0.44	0.19	0.29	0.38	0.14
v15	0.12	0.30	0.17	0.15	0.33	0.10	0.22	0.25	0.38	0.24	0.25	0.16	0.46	0.16	1.00	0.41	0.33	0.39	0.35	0.22	0.14	0.41	0.20	0.15	0.40	0.13	0.43	0.24	0.26	0.20
v16	0.16	0.41	0.23	0.13	0.30	0.12	0.34	0.20	0.39	0.29	0.28	0.20	0.38	0.29	0.41	1.00	0.33	0.25	0.33	0.27	0.15	0.42	0.19	0.20	0.38	0.19	0.43	0.23	0.27	0.12
v17	0.36	0.28	0.33	0.30	0.28	0.34	0.44	0.38	0.44	0.49	0.24	0.39	0.43	0.43	0.33	0.33	1.00	0.18	0.33	0.44	0.33	0.33	0.39	0.43	0.52	0.45	0.31	0.43	0.48	0.08
v18	0.03	0.18	0.11	0.06	0.18	0.04	0.12	0.13	0.24	0.15	0.18	0.09	0.35	0.16	0.39	0.25	0.18	1.00	0.23	0.15	0.09	0.24	0.08	0.06	0.25	0.10	0.32	0.20	0.21	0.26
v19	0.13	0.35	0.19	0.22	0.68	0.16	0.28	0.27	0.37	0.28	0.46	0.20	0.43	0.25	0.35	0.33	0.33	0.23	1.00	0.35	0.19	0.42	0.20	0.19	0.38	0.23	0.40	0.29	0.29	0.11
v20	0.27	0.23	0.41	0.22	0.26	0.32	0.36	0.31	0.38	0.43	0.30	0.36	0.31	0.31	0.22	0.27	0.44	0.15	0.35	1.00	0.25	0.28	0.28	0.37	0.42	0.42	0.26	0.37	0.46	0.10
v21	0.23	0.12	0.25	0.18	0.16	0.23	0.26	0.19	0.28	0.31	0.12	0.22	0.19	0.27	0.14	0.15	0.33	0.09	0.19	0.25	1.00	0.13	0.25	0.27	0.26	0.32	0.10	0.24	0.21	0.04
v22	0.15	0.42	0.17	0.12	0.40	0.07	0.28	0.27	0.43	0.29	0.38	0.16	0.51	0.20	0.41	0.42	0.33	0.24	0.42	0.28	0.13	1.00	0.21	0.16	0.43	0.19	0.51	0.29	0.32	0.15
v23	0.26	0.13	0.27	0.42	0.21	0.36	0.25	0.45	0.34	0.36	0.10	0.44	0.24	0.24	0.20	0.19	0.39	0.08	0.20	0.28	0.25	0.21	1.00	0.45	0.32	0.30	0.19	0.25	0.31	0.06
v24	0.37	0.10	0.29	0.34	0.13	0.43	0.32	0.38	0.36	0.44	0.07	0.61	0.27	0.25	0.15	0.20	0.43	0.06	0.19	0.37	0.27	0.16	0.45	1.00	0.41	0.34	0.20	0.38	0.29	0.05
v25	0.29	0.31	0.31	0.20	0.29	0.28	0.40	0.37	0.49	0.49	0.26	0.30	0.58	0.32	0.40	0.38	0.52	0.25	0.38	0.42	0.26	0.43	0.32	0.41	1.00	0.42	0.46	0.50	0.45	0.16
v26	0.30	0.16	0.37	0.25	0.17	0.30	0.35	0.27	0.34	0.49	0.21	0.33	0.27	0.44	0.13	0.19	0.45	0.10	0.23	0.42	0.32	0.19	0.30	0.34	0.42	1.00	0.17	0.35	0.49	0.09
v27	0.11	0.41	0.16	0.11	0.30	0.10	0.25	0.30	0.42	0.26	0.26	0.17	0.62	0.19	0.43	0.43	0.31	0.32	0.40	0.26	0.10	0.51	0.19	0.20	0.46	0.17	1.00	0.31	0.31	0.18
v28	0.28	0.20	0.24	0.17	0.23	0.30	0.34	0.34	0.38	0.45	0.19	0.24	0.44	0.29	0.24	0.23	0.43	0.20	0.29	0.37	0.24	0.29	0.25	0.38	0.50	0.35	0.31	1.00	0.41	0.11
v29	0.26	0.25	0.32	0.21	0.25	0.27	0.32	0.28	0.35	0.41	0.27	0.30	0.38	0.38	0.26	0.27	0.48	0.21	0.29	0.46	0.21	0.32	0.31	0.29	0.45	0.49	0.31	0.41	1.00	0.14
v30	0.02	0.12	0.13	0.06	0.09	0.10	0.09	0.15	0.16	0.15	0.13	0.05	0.16	0.14	0.20	0.12	0.08	0.26	0.11	0.10	0.04	0.15	0.06	0.05	0.16	0.09	0.18	0.11	0.14	1.00
v31	0.28	0.25	0.30	0.23	0.30	0.25	0.32	0.32	0.41	0.42	0.21	0.32	0.41	0.32	0.24	0.28	0.45	0.15	0.34	0.39	0.26	0.31	0.32	0.37	0.48	0.38	0.32	0.31	0.41	0.12

v31
0.28
0.25
0.30
0.23
0.30
0.25
0.32
0.32
0.41
0.42
0.21
0.32
0.41
0.32
0.24
0.28
0.45
0.15
0.34
0.39
0.26
0.31
0.32
0.37
0.48
0.38
0.32
0.31
0.41
0.12
1.00
