# Supplementary material for: Network models of driver behavior
Source: PeerJ. 2019 Jan 10;6:e6119. doi: 10.7717/peerj.6119 (PMC6330205; doi:10.7717/peerj.6119)
Supplement: Supplemental Information 6 [file peerj-07-6119-s006.pdf]

Variable	v1	v2	v3	v4	v5	v6	v7	v8	v9	v10	v11	v12	v13	v14	v15	v16	v17	v18	v19	v20	v21	v22	v23	v24	v25	v26	v27	v28	v29	v30
v1	0.00	0.00	0.00	0.05	0.00	0.15	0.01	0.07	0.06	0.06	0.00	0.04	0.00	0.00	0.00	0.00	0.07	0.00	0.00	0.00	0.03	0.00	0.00	0.07	0.00	0.03	0.00	0.02	0.00	0.00
v2	0.00	0.00	0.05	0.00	0.10	0.00	0.02	0.00	0.06	0.00	0.12	0.00	0.05	0.00	0.00	0.16	0.00	0.00	0.00	0.00	0.00	0.10	0.00	0.00	0.00	0.00	0.10	0.00	0.00	0.00
v3	0.00	0.05	0.00	0.00	0.00	0.07	0.03	0.00	0.06	0.08	0.07	0.00	0.00	0.09	0.00	0.00	0.00	0.00	0.00	0.15	0.04	0.00	0.02	0.01	0.00	0.08	0.00	0.00	0.01	0.01
v4	0.05	0.00	0.00	0.00	0.02	0.02	0.00	0.12	0.01	0.00	0.00	0.07	0.00	0.05	0.00	0.00	0.02	0.00	0.02	0.00	0.00	0.00	0.19	0.05	0.00	0.02	0.00	0.00	0.00	0.00
v5	0.00	0.10	0.00	0.02	0.00	0.00	0.02	0.01	0.00	0.00	0.17	0.00	0.00	0.00	0.05	0.00	0.00	0.00	0.48	0.00	0.00	0.06	0.00	0.00	0.00	0.00	0.00	0.00	0.00	0.00
v6	0.15	0.00	0.07	0.02	0.00	0.00	0.04	0.00	0.00	0.04	0.00	0.07	0.00	0.00	0.00	0.00	0.01	0.00	0.00	0.04	0.02	-0.02	0.10	0.13	0.00	0.02	0.00	0.04	0.00	0.00
v7	0.01	0.02	0.03	0.00	0.02	0.04	0.00	0.00	0.09	0.08	0.00	0.00	0.00	0.01	0.00	0.10	0.12	0.00	0.00	0.05	0.03	0.00	0.00	0.02	0.05	0.04	0.00	0.04	0.00	0.00
v8	0.07	0.00	0.00	0.12	0.01	0.00	0.00	0.00	0.09	0.04	0.00	0.00	0.02	0.00	0.00	0.00	0.05	0.00	0.02	0.01	0.00	0.00	0.20	0.05	0.02	0.00	0.04	0.06	0.00	0.03
v9	0.06	0.06	0.06	0.01	0.00	0.00	0.09	0.09	0.00	0.10	0.04	0.00	0.13	0.00	0.05	0.06	0.03	0.00	0.01	0.03	0.04	0.09	0.03	0.01	0.06	0.00	0.01	0.01	0.00	0.01
v10	0.06	0.00	0.08	0.00	0.00	0.04	0.08	0.04	0.10	0.00	0.00	0.05	0.00	0.08	0.00	0.00	0.06	0.00	0.00	0.05	0.04	0.00	0.01	0.07	0.08	0.15	0.00	0.11	0.01	0.00
v11	0.00	0.12	0.07	0.00	0.17	0.00	0.00	0.00	0.04	0.00	0.00	0.00	0.00	0.03	0.00	0.00	0.00	0.00	0.14	0.05	0.00	0.09	0.00	-0.03	0.00	0.00	0.00	0.00	0.03	0.01
v12	0.04	0.00	0.00	0.07	0.00	0.07	0.00	0.00	0.00	0.05	0.00	0.00	0.00	0.00	0.00	0.00	0.04	0.00	0.00	0.07	0.00	0.00	0.12	0.36	0.00	0.03	0.00	0.00	0.00	0.00
v13	0.00	0.05	0.00	0.00	0.00	0.00	0.00	0.02	0.13	0.00	0.00	0.00	0.00	0.00	0.09	0.00	0.03	0.10	0.07	0.00	0.00	0.10	0.00	0.00	0.18	0.00	0.30	0.10	0.02	0.00
v14	0.00	0.00	0.09	0.05	0.00	0.00	0.01	0.00	0.00	0.08	0.03	0.00	0.00	0.00	0.00	0.07	0.12	0.00	0.00	0.00	0.05	0.00	0.00	0.00	0.00	0.15	0.00	0.00	0.07	0.02
v15	0.00	0.00	0.00	0.00	0.05	0.00	0.00	0.00	0.05	0.00	0.00	0.00	0.09	0.00	0.00	0.14	0.04	0.20	0.03	0.00	0.00	0.08	0.00	0.00	0.06	0.00	0.08	0.00	0.00	0.05
v16	0.00	0.16	0.00	0.00	0.00	0.00	0.10	0.00	0.06	0.00	0.00	0.00	0.00	0.07	0.14	0.00	0.01	0.02	0.02	0.00	0.00	0.10	0.00	0.00	0.04	0.00	0.11	0.00	0.00	0.00
v17	0.07	0.00	0.00	0.02	0.00	0.01	0.12	0.05	0.03	0.06	0.00	0.04	0.03	0.12	0.04	0.01	0.00	0.00	0.00	0.06	0.08	0.00	0.06	0.03	0.10	0.04	0.00	0.05	0.11	0.00
v18	0.00	0.00	0.00	0.00	0.00	0.00	0.00	0.00	0.00	0.00	0.00	0.00	0.10	0.00	0.20	0.02	0.00	0.00	0.01	0.00	0.00	0.00	0.00	0.00	0.00	0.00	0.06	0.00	0.02	0.14
v19	0.00	0.00	0.00	0.02	0.48	0.00	0.00	0.02	0.01	0.00	0.14	0.00	0.07	0.00	0.03	0.02	0.00	0.01	0.00	0.07	0.00	0.05	0.00	0.00	0.02	0.00	0.05	0.00	0.00	0.00
v20	0.00	0.00	0.15	0.00	0.00	0.04	0.05	0.01	0.03	0.05	0.05	0.07	0.00	0.00	0.00	0.00	0.06	0.00	0.07	0.00	0.00	0.00	0.00	0.02	0.03	0.07	0.00	0.04	0.15	0.00
v21	0.03	0.00	0.04	0.00	0.00	0.02	0.03	0.00	0.04	0.04	0.00	0.00	0.00	0.05	0.00	0.00	0.08	0.00	0.00	0.00	0.00	0.00	0.04	0.03	0.00	0.09	0.00	0.01	0.00	0.00
v22	0.00	0.10	0.00	0.00	0.06	-0.02	0.00	0.00	0.09	0.00	0.09	0.00	0.10	0.00	0.08	0.10	0.00	0.00	0.05	0.00	0.00	0.00	0.00	0.00	0.05	0.00	0.16	0.00	0.03	0.00
v23	0.00	0.00	0.02	0.19	0.00	0.10	0.00	0.20	0.03	0.01	0.00	0.12	0.00	0.00	0.00	0.00	0.06	0.00	0.00	0.00	0.04	0.00	0.00	0.10	0.00	0.00	0.00	0.00	0.03	0.00
v24	0.07	0.00	0.01	0.05	0.00	0.13	0.02	0.05	0.01	0.07	-0.03	0.36	0.00	0.00	0.00	0.00	0.03	0.00	0.00	0.02	0.03	0.00	0.10	0.00	0.06	0.00	0.00	0.06	0.00	0.00
v25	0.00	0.00	0.00	0.00	0.00	0.00	0.05	0.02	0.06	0.08	0.00	0.00	0.18	0.00	0.06	0.04	0.10	0.00	0.02	0.03	0.00	0.05	0.00	0.06	0.00	0.04	0.05	0.16	0.06	0.00
v26	0.03	0.00	0.08	0.02	0.00	0.02	0.04	0.00	0.00	0.15	0.00	0.03	0.00	0.15	0.00	0.00	0.04	0.00	0.00	0.07	0.09	0.00	0.00	0.00	0.04	0.00	0.00	0.00	0.19	0.00
v27	0.00	0.10	0.00	0.00	0.00	0.00	0.00	0.04	0.01	0.00	0.00	0.00	0.30	0.00	0.08	0.11	0.00	0.06	0.05	0.00	0.00	0.16	0.00	0.00	0.05	0.00	0.00	0.00	0.00	0.03
v28	0.02	0.00	0.00	0.00	0.00	0.04	0.04	0.06	0.01	0.11	0.00	0.00	0.10	0.00	0.00	0.00	0.05	0.00	0.00	0.04	0.01	0.00	0.00	0.06	0.16	0.00	0.00	0.00	0.10	0.00
v29	0.00	0.00	0.01	0.00	0.00	0.00	0.00	0.00	0.00	0.01	0.03	0.00	0.02	0.07	0.00	0.00	0.11	0.02	0.00	0.15	0.00	0.03	0.03	0.00	0.06	0.19	0.00	0.10	0.00	0.01
v30	0.00	0.00	0.01	0.00	0.00	0.00	0.00	0.03	0.01	0.00	0.01	0.00	0.00	0.02	0.05	0.00	0.00	0.14	0.00	0.00	0.00	0.00	0.00	0.00	0.00	0.00	0.03	0.00	0.01	0.00
v31	0.01	0.00	0.01	0.00	0.02	0.00	0.00	0.02	0.07	0.05	0.00	0.01	0.04	0.03	0.00	0.00	0.09	0.00	0.05	0.05	0.02	0.00	0.03	0.05	0.13	0.04	0.00	0.00	0.08	0.00

v31
0.01
0.00
0.01
0.00
0.02
0.00
0.00
0.02
0.07
0.05
0.00
0.01
0.04
0.03
0.00
0.00
0.09
0.00
0.05
0.05
0.02
0.00
0.03
0.05
0.13
0.04
0.00
0.00
0.08
0.00
0.00
