# Supplementary material for: Network models of driver behavior
Source: PeerJ. 2019 Jan 10;6:e6119. doi: 10.7717/peerj.6119 (PMC6330205; doi:10.7717/peerj.6119)
Supplement: Supplemental Information 7 [file peerj-07-6119-s007.pdf]

Variable	v1	v2	v3	v4	v5	v6	v7	v8	v9	v10	v11	v12	v13	v14	v15	v16	v17	v18	v19	v20	v21	v22	v23	v24	v25	v26	v27	v28	v29	v30	v31	v32	v33	v34	v35	v36	v37	v38	v39	v40
v1	1.00	0.17	0.28	0.15	0.08	0.25	0.26	0.24	0.24	0.27	0.08	0.23	0.19	0.25	0.13	0.19	0.26	0.13	0.08	0.26	0.18	0.10	0.20	0.26	0.24	0.28	0.15	0.22	0.19	0.07	0.18	-0.03	-0.07	0.10	0.05	0.26	0.17	0.21	0.10	0.26
v2	0.17	1.00	0.37	0.12	0.29	0.06	0.24	0.16	0.32	0.19	0.32	0.15	0.33	0.24	0.32	0.36	0.25	0.28	0.30	0.22	0.16	0.36	0.13	0.13	0.27	0.25	0.34	0.12	0.21	0.27	0.20	-0.11	-0.24	0.18	0.25	0.00	-0.02	0.05	-0.14	0.17
v3	0.28	0.37	1.00	0.14	0.19	0.23	0.36	0.20	0.27	0.36	0.30	0.22	0.21	0.38	0.20	0.30	0.30	0.20	0.18	0.37	0.17	0.18	0.17	0.22	0.26	0.39	0.18	0.20	0.29	0.22	0.21	0.00	-0.06	0.13	0.06	0.19	0.06	0.17	0.05	0.22
v4	0.15	0.12	0.14	1.00	0.21	0.24	0.20	0.25	0.17	0.21	0.14	0.31	0.18	0.26	0.15	0.17	0.27	0.15	0.18	0.20	0.17	0.16	0.31	0.26	0.18	0.24	0.16	0.14	0.19	0.09	0.19	-0.01	-0.02	0.10	0.07	0.05	0.14	0.10	0.02	0.12
v5	0.08	0.29	0.19	0.21	1.00	0.06	0.21	0.14	0.24	0.16	0.47	0.14	0.29	0.22	0.38	0.26	0.21	0.23	0.61	0.20	0.13	0.40	0.13	0.09	0.22	0.15	0.30	0.12	0.22	0.27	0.23	-0.15	-0.09	0.37	0.27	-0.10	-0.10	-0.02	-0.17	0.09
v6	0.25	0.06	0.23	0.24	0.06	1.00	0.27	0.21	0.18	0.30	0.06	0.29	0.16	0.23	0.08	0.09	0.25	0.07	0.09	0.25	0.19	0.04	0.24	0.32	0.22	0.30	0.06	0.23	0.19	0.05	0.16	0.04	0.00	0.03	-0.04	0.30	0.26	0.20	0.21	0.19
v7	0.26	0.24	0.36	0.20	0.21	0.27	1.00	0.26	0.37	0.39	0.26	0.29	0.34	0.36	0.27	0.32	0.37	0.22	0.25	0.33	0.26	0.28	0.24	0.32	0.36	0.44	0.24	0.28	0.29	0.23	0.28	-0.05	-0.07	0.13	0.10	0.17	0.19	0.20	0.10	0.25
v8	0.24	0.16	0.20	0.25	0.14	0.21	0.26	1.00	0.36	0.36	0.16	0.29	0.30	0.23	0.19	0.22	0.34	0.23	0.19	0.27	0.18	0.22	0.34	0.31	0.32	0.32	0.26	0.27	0.26	0.21	0.24	-0.07	-0.10	0.11	0.11	0.13	0.16	0.19	0.06	0.22
v9	0.24	0.32	0.27	0.17	0.24	0.18	0.37	0.36	1.00	0.41	0.28	0.22	0.46	0.32	0.38	0.34	0.35	0.41	0.31	0.28	0.22	0.38	0.25	0.29	0.39	0.36	0.40	0.32	0.27	0.31	0.27	-0.13	-0.11	0.17	0.26	0.11	0.11	0.16	-0.01	0.25
v10	0.27	0.19	0.36	0.21	0.16	0.30	0.39	0.36	0.41	1.00	0.28	0.32	0.36	0.44	0.25	0.28	0.44	0.25	0.21	0.40	0.27	0.27	0.28	0.38	0.41	0.50	0.26	0.37	0.35	0.23	0.31	-0.08	-0.09	0.12	0.11	0.19	0.20	0.31	0.12	0.26
v11	0.08	0.32	0.30	0.14	0.47	0.06	0.26	0.16	0.28	0.28	1.00	0.15	0.36	0.31	0.35	0.33	0.24	0.30	0.58	0.29	0.13	0.46	0.11	0.08	0.27	0.25	0.31	0.18	0.29	0.35	0.21	-0.13	-0.09	0.36	0.31	-0.06	-0.08	0.01	-0.11	0.17
v12	0.23	0.15	0.22	0.31	0.14	0.29	0.29	0.29	0.22	0.32	0.15	1.00	0.22	0.27	0.15	0.23	0.32	0.16	0.19	0.31	0.24	0.17	0.36	0.49	0.25	0.32	0.24	0.22	0.25	0.15	0.27	-0.05	-0.03	0.09	0.06	0.14	0.18	0.19	0.04	0.19
v13	0.19	0.33	0.21	0.18	0.29	0.16	0.34	0.30	0.46	0.36	0.36	0.22	1.00	0.33	0.50	0.36	0.40	0.45	0.39	0.34	0.25	0.53	0.24	0.28	0.52	0.33	0.61	0.41	0.37	0.39	0.31	-0.26	-0.19	0.26	0.48	0.07	0.14	0.17	-0.05	0.37
v14	0.25	0.24	0.38	0.26	0.22	0.23	0.36	0.23	0.32	0.44	0.31	0.27	0.33	1.00	0.22	0.33	0.42	0.27	0.25	0.40	0.24	0.28	0.25	0.32	0.37	0.42	0.23	0.29	0.36	0.30	0.31	-0.04	-0.07	0.15	0.10	0.16	0.14	0.21	0.15	0.25
v15	0.13	0.32	0.20	0.15	0.38	0.08	0.27	0.19	0.38	0.25	0.35	0.15	0.50	0.22	1.00	0.40	0.37	0.52	0.40	0.27	0.23	0.47	0.18	0.18	0.41	0.26	0.48	0.27	0.35	0.46	0.27	-0.20	-0.14	0.25	0.41	-0.06	-0.03	0.09	-0.20	0.24
v16	0.19	0.36	0.30	0.17	0.26	0.09	0.32	0.22	0.34	0.28	0.33	0.23	0.36	0.33	0.40	1.00	0.38	0.33	0.33	0.33	0.19	0.38	0.21	0.22	0.38	0.32	0.44	0.19	0.31	0.34	0.26	-0.19	-0.11	0.17	0.28	0.04	-0.04	0.09	-0.19	0.22
v17	0.26	0.25	0.30	0.27	0.21	0.25	0.37	0.34	0.35	0.44	0.24	0.32	0.40	0.42	0.37	0.38	1.00	0.32	0.30	0.41	0.29	0.34	0.32	0.36	0.46	0.46	0.35	0.32	0.39	0.28	0.36	-0.11	-0.11	0.17	0.20	0.13	0.16	0.22	0.02	0.28
v18	0.13	0.28	0.20	0.15	0.23	0.07	0.22	0.23	0.41	0.25	0.30	0.16	0.45	0.27	0.52	0.33	0.32	1.00	0.35	0.26	0.23	0.37	0.19	0.16	0.39	0.34	0.42	0.27	0.33	0.58	0.29	-0.18	-0.08	0.17	0.37	0.04	0.03	0.09	-0.09	0.25
v19	0.08	0.30	0.18	0.18	0.61	0.09	0.25	0.19	0.31	0.21	0.58	0.19	0.39	0.25	0.40	0.33	0.30	0.35	1.00	0.32	0.18	0.48	0.18	0.16	0.34	0.23	0.38	0.22	0.29	0.33	0.30	-0.17	-0.10	0.46	0.29	-0.02	-0.03	0.07	-0.11	0.20
v20	0.26	0.22	0.37	0.20	0.20	0.25	0.33	0.27	0.28	0.40	0.29	0.31	0.34	0.40	0.27	0.33	0.41	0.26	0.32	1.00	0.25	0.28	0.26	0.33	0.38	0.42	0.28	0.32	0.39	0.21	0.33	-0.07	-0.08	0.16	0.15	0.18	0.16	0.25	0.12	0.30
v21	0.18	0.16	0.17	0.17	0.13	0.19	0.26	0.18	0.22	0.27	0.13	0.24	0.25	0.24	0.23	0.19	0.29	0.23	0.18	0.25	1.00	0.18	0.23	0.25	0.26	0.32	0.17	0.22	0.29	0.24	0.24	-0.03	-0.08	0.09	0.09	0.11	0.19	0.14	0.06	0.16
v22	0.10	0.36	0.18	0.16	0.40	0.04	0.28	0.22	0.38	0.27	0.46	0.17	0.53	0.28	0.47	0.38	0.34	0.37	0.48	0.28	0.18	1.00	0.20	0.20	0.42	0.25	0.53	0.28	0.33	0.41	0.29	-0.27	-0.20	0.30	0.47	-0.05	-0.01	0.09	-0.19	0.24
v23	0.20	0.13	0.17	0.31	0.13	0.24	0.24	0.34	0.25	0.28	0.11	0.36	0.24	0.25	0.18	0.21	0.32	0.19	0.18	0.26	0.23	0.20	1.00	0.37	0.27	0.32	0.21	0.21	0.28	0.19	0.25	-0.04	-0.05	0.08	0.09	0.08	0.13	0.14	0.05	0.15
v24	0.26	0.13	0.22	0.26	0.09	0.32	0.32	0.31	0.29	0.38	0.08	0.49	0.28	0.32	0.18	0.22	0.36	0.16	0.16	0.33	0.25	0.20	0.37	1.00	0.38	0.39	0.24	0.32	0.28	0.16	0.28	-0.04	-0.08	0.07	0.04	0.20	0.27	0.25	0.12	0.23
v25	0.24	0.27	0.26	0.18	0.22	0.22	0.36	0.32	0.39	0.41	0.27	0.25	0.52	0.37	0.41	0.38	0.46	0.39	0.34	0.38	0.26	0.42	0.27	0.38	1.00	0.47	0.46	0.43	0.42	0.35	0.35	-0.18	-0.16	0.24	0.30	0.12	0.11	0.25	0.00	0.36
v26	0.28	0.25	0.39	0.24	0.15	0.30	0.44	0.32	0.36	0.50	0.25	0.32	0.33	0.42	0.26	0.32	0.46	0.34	0.23	0.42	0.32	0.25	0.32	0.39	0.47	1.00	0.28	0.35	0.46	0.32	0.37	-0.02	-0.10	0.13	0.10	0.20	0.18	0.26	0.14	0.30
v27	0.15	0.34	0.18	0.16	0.30	0.06	0.24	0.26	0.40	0.26	0.31	0.24	0.61	0.23	0.48	0.44	0.35	0.42	0.38	0.28	0.17	0.53	0.21	0.24	0.46	0.28	1.00	0.31	0.34	0.39	0.31	-0.37	-0.19	0.23	0.52	0.03	0.03	0.11	-0.35	0.30
v28	0.22	0.12	0.20	0.14	0.12	0.23	0.28	0.27	0.32	0.37	0.18	0.22	0.41	0.29	0.27	0.19	0.32	0.27	0.22	0.32	0.22	0.28	0.21	0.32	0.43	0.35	0.31	1.00	0.38	0.25	0.26	-0.12	-0.12	0.15	0.18	0.15	0.21	0.24	0.14	0.27
v29	0.19	0.21	0.29	0.19	0.22	0.19	0.29	0.26	0.27	0.35	0.29	0.25	0.37	0.36	0.35	0.31	0.39	0.33	0.29	0.39	0.29	0.33	0.28	0.28	0.42	0.46	0.34	0.38	1.00	0.33	0.40	-0.13	-0.10	0.18	0.18	0.09	0.10	0.19	0.03	0.28
v30	0.07	0.27	0.22	0.09	0.27	0.05	0.23	0.21	0.31	0.23	0.35	0.15	0.39	0.30	0.46	0.34	0.28	0.58	0.33	0.21	0.24	0.41	0.19	0.16	0.35	0.32	0.39	0.25	0.33	1.00	0.28	-0.19	-0.11	0.23	0.28	-0.05	-0.03	0.08	-0.14	0.19
v31	0.18	0.20	0.21	0.19	0.23	0.16	0.28	0.24	0.27	0.31	0.21	0.27	0.31	0.31	0.27	0.26	0.36	0.29	0.30	0.33	0.24	0.29	0.25	0.28	0.35	0.37	0.31	0.26	0.40	0.28	1.00	-0.08	-0.07	0.17	0.17	0.13	0.11	0.19	0.01	0.28
v32	-0.03	-0.11	0.00	-0.01	-0.15	0.04	-0.05	-0.07	-0.13	-0.08	-0.13	-0.05	-0.26	-0.04	-0.20	-0.19	-0.11	-0.18	-0.17	-0.07	-0.03	-0.27	-0.04	-0.04	-0.18	-0.02	-0.37	-0.12	-0.13	-0.19	-0.08	1.00	0.21	-0.12	-0.32	0.09	0.03	-0.01	0.22	-0.11
v33	-0.07	-0.24	-0.06	-0.02	-0.09	0.00	-0.07	-0.10	-0.11	-0.09	-0.09	-0.03	-0.19	-0.07	-0.14	-0.11	-0.11	-0.08	-0.10	-0.08	-0.08	-0.20	-0.05	-0.08	-0.16	-0.10	-0.19	-0.12	-0.10	-0.11	-0.07	0.21	1.00	-0.10	-0.17	-0.01	-0.05	-0.07	0.05	-0.10
v34	0.10	0.18	0.13	0.10	0.37	0.03	0.13	0.11	0.17	0.12	0.36	0.09	0.26	0.15	0.25	0.17	0.17	0.17	0.46	0.16	0.09	0.30	0.08	0.07	0.24	0.13	0.23	0.15	0.18	0.23	0.17	-0.12	-0.10	1.00	0.22	0.02	0.01	0.05	-0.06	0.16
v35	0.05	0.25	0.06	0.07	0.27	-0.04	0.10	0.11	0.26	0.11	0.31	0.06	0.48	0.10	0.41	0.28	0.20	0.37	0.29	0.15	0.09	0.47	0.09	0.04	0.30	0.10	0.52	0.18	0.18	0.28	0.17	-0.32	-0.17	0.22	1.00	-0.12	-0.10	-0.01	-0.26	0.22
v36	0.26	0.00	0.19	0.05	-0.10	0.30	0.17	0.13	0.11	0.19	-0.06	0.14	0.07	0.16	-0.06	0.04	0.13	0.04	-0.02	0.18	0.11	-0.05	0.08	0.20	0.12	0.20	0.03	0.15	0.09	-0.05	0.13	0.09	-0.01	0.02	-0.12	1.00	0.40	0.46	0.39	0.45
v37	0.17	-0.02	0.06	0.14	-0.10	0.26	0.19	0.16	0.11	0.20	-0.08	0.18	0.14	0.14	-0.03	-0.04	0.16	0.03	-0.03	0.16	0.19	-0.01	0.13	0.27	0.11	0.18	0.03	0.21	0.10	-0.03	0.11	0.03	-0.05	0.01	-0.10	0.40	1.00	0.29	0.34	0.21
v38	0.21	0.05	0.17	0.10	-0.02	0.20	0.20	0.19	0.16	0.31	0.01	0.19	0.17	0.21	0.09	0.09	0.22	0.09	0.07	0.25	0.14	0.09	0.14	0.25	0.25	0.26	0.11	0.24	0.19	0.08	0.19	-0.01	-0.07	0.05	-0.01	0.46	0.29	1.00	0.32	0.53
v39	0.10	-0.14	0.05	0.02	-0.17	0.21	0.10	0.06	-0.01	0.12	-0.11	0.04	-0.05	0.15	-0.20	-0.19	0.02	-0.09	-0.11	0.12	0.06	-0.19	0.05	0.12	0.00	0.14	-0.35	0.14	0.03	-0.14	0.01	0.22	0.05	-0.06	-0.26	0.39	0.34	0.32	1.00	0.22
v40	0.26	0.17	0.22	0.12	0.09	0.19	0.25	0.22	0.25	0.26	0.17	0.19	0.37	0.25	0.24	0.22	0.28	0.25	0.20	0.30	0.16	0.24	0.15	0.23	0.36	0.30	0.30	0.27	0.28	0.19	0.28	-0.11	-0.10	0.16	0.22	0.45	0.21	0.53	0.22	1.00
