# Supplementary material for: Network models of driver behavior
Source: PeerJ. 2019 Jan 10;6:e6119. doi: 10.7717/peerj.6119 (PMC6330205; doi:10.7717/peerj.6119)
Supplement: Supplemental Information 8 [file peerj-07-6119-s008.pdf]

Variable	v1	v2	v3	v4	v5	v6	v7	v8	v9	v10	v11	v12	v13	v14	v15	v16	v17	v18	v19	v20	v21	v22	v23	v24	v25	v26	v27	v28	v29	v30	v31	v32	v33	v34	v35	v36	v37	v38	v39	v40
v1	0.00	0.03	0.09	0.00	0.00	0.07	0.04	0.06	0.05	0.01	-0.02	0.02	0.00	0.03	0.00	0.02	0.03	0.00	-0.02	0.04	0.02	-0.01	0.01	0.04	0.00	0.02	0.00	0.02	0.00	-0.04	0.00	0.00	0.00	0.02	0.00	0.10	0.00	0.00	0.00	0.05
v2	0.03	0.00	0.22	0.00	0.07	-0.01	0.00	0.00	0.08	-0.03	0.04	0.00	0.03	0.00	0.03	0.11	0.00	0.02	0.00	0.00	0.00	0.08	0.00	0.00	0.00	0.01	0.04	-0.05	0.00	0.01	0.00	0.03	-0.15	0.00	0.01	-0.01	0.00	0.00	-0.04	0.00
v3	0.09	0.22	0.00	0.00	0.01	0.04	0.11	0.00	0.00	0.07	0.12	0.00	-0.03	0.11	0.00	0.05	0.00	0.00	-0.05	0.11	0.00	-0.04	-0.01	0.00	0.00	0.09	-0.01	0.00	0.01	0.01	0.00	0.03	0.01	0.00	-0.02	0.06	-0.04	0.00	0.00	0.00
v4	0.00	0.00	0.00	0.00	0.10	0.10	0.00	0.08	0.00	0.00	0.00	0.12	0.00	0.08	0.00	0.00	0.05	0.01	0.00	0.00	0.01	0.00	0.14	0.01	0.00	0.01	0.00	-0.01	0.00	-0.04	0.00	0.01	0.01	0.00	0.00	-0.03	0.05	0.00	-0.01	0.00
v5	0.00	0.07	0.01	0.10	0.00	0.00	0.03	0.00	0.00	0.00	0.10	0.00	0.00	0.01	0.12	0.00	0.00	-0.04	0.37	0.00	0.00	0.05	0.00	0.00	-0.01	-0.03	0.00	-0.02	0.00	0.00	0.01	0.00	0.00	0.09	0.00	-0.02	-0.06	-0.02	-0.02	-0.01
v6	0.07	-0.01	0.04	0.10	0.00	0.00	0.06	0.00	0.00	0.06	0.00	0.08	0.00	0.00	0.00	-0.01	0.02	-0.02	0.00	0.02	0.02	-0.04	0.04	0.07	0.01	0.04	-0.01	0.04	0.00	-0.01	0.00	0.01	0.01	0.00	-0.01	0.11	0.06	0.00	0.04	0.00
v7	0.04	0.00	0.11	0.00	0.03	0.06	0.00	0.00	0.10	0.04	0.03	0.04	0.04	0.05	0.02	0.08	0.04	-0.02	0.00	0.00	0.04	0.03	0.00	0.03	0.02	0.12	0.00	0.00	0.00	0.00	0.02	0.00	0.00	0.00	-0.04	0.00	0.05	0.00	0.03	0.00
v8	0.06	0.00	0.00	0.08	0.00	0.00	0.00	0.00	0.14	0.09	0.00	0.04	0.02	-0.02	-0.02	0.00	0.06	0.00	0.00	0.01	0.00	0.00	0.15	0.03	0.03	0.01	0.02	0.03	0.00	0.02	0.01	0.00	-0.01	0.00	-0.01	0.00	0.02	0.01	0.01	0.00
v9	0.05	0.08	0.00	0.00	0.00	0.00	0.10	0.14	0.00	0.12	0.00	0.00	0.10	0.02	0.03	0.02	0.00	0.13	0.02	0.00	0.00	0.04	0.01	0.03	0.01	0.01	0.05	0.04	-0.03	0.00	0.00	0.00	0.00	0.00	0.00	0.00	0.00	0.00	0.00	0.00
v10	0.01	-0.03	0.07	0.00	0.00	0.06	0.04	0.09	0.12	0.00	0.07	0.03	0.02	0.12	0.00	0.00	0.09	-0.01	-0.02	0.06	0.02	0.00	0.00	0.06	0.03	0.15	0.00	0.08	0.00	0.00	0.01	0.00	0.00	-0.01	-0.02	0.00	0.01	0.11	0.00	-0.02
v11	-0.02	0.04	0.12	0.00	0.10	0.00	0.03	0.00	0.00	0.07	0.00	0.00	0.01	0.07	0.00	0.03	-0.01	0.00	0.28	0.03	-0.01	0.12	-0.03	-0.07	-0.01	0.00	-0.01	0.00	0.03	0.06	-0.01	0.01	0.00	0.09	0.04	-0.03	-0.04	-0.04	0.00	0.01
v12	0.02	0.00	0.00	0.12	0.00	0.08	0.04	0.04	0.00	0.03	0.00	0.00	0.00	0.00	-0.02	0.02	0.02	0.00	0.01	0.06	0.05	0.00	0.12	0.29	-0.03	0.00	0.04	0.00	0.00	0.00	0.04	0.00	0.01	0.00	-0.02	0.00	0.00	0.00	-0.01	0.00
v13	0.00	0.03	-0.03	0.00	0.00	0.00	0.04	0.02	0.10	0.02	0.01	0.00	0.00	0.02	0.10	0.00	0.03	0.05	0.02	0.00	0.01	0.11	0.00	0.00	0.11	0.00	0.24	0.11	0.00	0.00	0.00	-0.01	-0.02	0.01	0.16	-0.02	0.06	0.00	0.06	0.07
v14	0.03	0.00	0.11	0.08	0.01	0.00	0.05	-0.02	0.02	0.12	0.07	0.00	0.02	0.00	-0.04	0.08	0.11	0.00	0.00	0.08	0.00	0.02	0.00	0.03	0.01	0.05	0.00	0.00	0.05	0.07	0.03	0.01	0.00	0.00	-0.03	0.00	0.00	0.00	0.10	0.00
v15	0.00	0.03	0.00	0.00	0.12	0.00	0.02	-0.02	0.03	0.00	0.00	-0.02	0.10	-0.04	0.00	0.08	0.09	0.21	0.01	0.00	0.03	0.06	0.00	0.00	0.04	-0.01	0.04	0.01	0.05	0.09	0.00	0.00	0.00	0.01	0.06	-0.06	0.00	0.01	-0.06	0.03
v16	0.02	0.11	0.05	0.00	0.00	-0.01	0.08	0.00	0.02	0.00	0.03	0.02	0.00	0.08	0.08	0.00	0.08	0.00	0.01	0.07	0.00	0.03	0.02	0.00	0.05	0.01	0.10	-0.02	0.01	0.04	0.00	-0.02	0.00	0.00	0.00	0.02	-0.07	0.00	-0.10	0.01
v17	0.03	0.00	0.00	0.05	0.00	0.02	0.04	0.06	0.00	0.09	-0.01	0.02	0.03	0.11	0.09	0.08	0.00	0.00	0.01	0.07	0.05	0.02	0.06	0.03	0.10	0.07	0.00	0.00	0.03	0.00	0.06	0.00	0.00	0.00	0.00	0.00	0.01	0.01	0.00	0.00
v18	0.00	0.02	0.00	0.01	-0.04	-0.02	-0.02	0.00	0.13	-0.01	0.00	0.00	0.05	0.00	0.21	0.00	0.00	0.00	0.06	0.00	0.01	0.00	0.00	-0.03	0.04	0.06	0.01	0.01	0.02	0.36	0.02	0.00	0.03	-0.03	0.09	0.03	0.00	-0.01	0.00	0.02
v19	-0.02	0.00	-0.05	0.00	0.37	0.00	0.00	0.00	0.02	-0.02	0.28	0.01	0.02	0.00	0.01	0.01	0.01	0.06	0.00	0.08	0.01	0.09	0.01	0.00	0.01	0.00	0.01	0.00	0.00	0.00	0.07	0.00	0.00	0.22	0.00	0.00	0.00	0.00	0.00	0.00
v20	0.04	0.00	0.11	0.00	0.00	0.02	0.00	0.01	0.00	0.06	0.03	0.06	0.00	0.08	0.00	0.07	0.07	0.00	0.08	0.00	0.02	0.00	0.01	0.03	0.03	0.05	0.01	0.04	0.09	-0.03	0.05	0.00	0.00	0.00	0.00	0.00	0.01	0.02	0.06	0.03
v21	0.02	0.00	0.00	0.01	0.00	0.02	0.04	0.00	0.00	0.02	-0.01	0.05	0.01	0.00	0.03	0.00	0.05	0.01	0.01	0.02	0.00	0.00	0.04	0.01	0.00	0.06	-0.01	0.01	0.06	0.07	0.03	0.01	-0.01	0.00	0.00	0.00	0.09	0.00	0.00	0.00
v22	-0.01	0.08	-0.04	0.00	0.05	-0.04	0.03	0.00	0.04	0.00	0.12	0.00	0.11	0.02	0.06	0.03	0.02	0.00	0.09	0.00	0.00	0.00	0.01	0.01	0.06	-0.01	0.10	0.02	0.02	0.07	0.01	-0.05	-0.04	0.02	0.14	-0.02	0.00	0.00	-0.01	0.01
v23	0.01	0.00	-0.01	0.14	0.00	0.04	0.00	0.15	0.01	0.00	-0.03	0.12	0.00	0.00	0.00	0.02	0.06	0.00	0.01	0.01	0.04	0.01	0.00	0.12	0.00	0.05	0.00	0.00	0.05	0.01	0.03	0.00	0.00	0.00	0.00	-0.01	0.00	0.00	0.00	-0.01
v24	0.04	0.00	0.00	0.01	0.00	0.07	0.03	0.03	0.03	0.06	-0.07	0.29	0.00	0.03	0.00	0.00	0.03	-0.03	0.00	0.03	0.01	0.01	0.12	0.00	0.09	0.06	0.02	0.05	0.00	0.00	0.03	0.00	0.00	-0.01	-0.03	0.00	0.10	0.03	0.00	0.00
v25	0.00	0.00	0.00	0.00	-0.01	0.01	0.02	0.03	0.01	0.03	-0.01	-0.03	0.11	0.01	0.04	0.05	0.10	0.04	0.01	0.03	0.00	0.06	0.00	0.09	0.00	0.13	0.08	0.14	0.05	0.01	0.02	0.00	-0.02	0.05	0.00	-0.01	-0.01	0.03	0.00	0.06
v26	0.02	0.01	0.09	0.01	-0.03	0.04	0.12	0.01	0.01	0.15	0.00	0.00	0.00	0.05	-0.01	0.01	0.07	0.06	0.00	0.05	0.06	-0.01	0.05	0.06	0.13	0.00	0.00	0.00	0.15	0.05	0.06	0.04	0.00	-0.01	-0.03	0.00	0.00	0.01	0.05	0.00
v27	0.00	0.04	-0.01	0.00	0.00	-0.01	0.00	0.02	0.05	0.00	-0.01	0.04	0.24	0.00	0.04	0.10	0.00	0.01	0.01	0.01	-0.01	0.10	0.00	0.02	0.08	0.00	0.00	0.04	0.02	0.03	0.04	-0.15	0.00	0.00	0.17	0.05	0.03	0.00	-0.31	0.08
v28	0.02	-0.05	0.00	-0.01	-0.02	0.04	0.00	0.03	0.04	0.08	0.00	0.00	0.11	0.00	0.01	-0.02	0.00	0.01	0.00	0.04	0.01	0.02	0.00	0.05	0.14	0.00	0.04	0.00	0.13	0.02	0.00	-0.01	-0.02	0.00	0.00	0.00	0.04	0.02	0.09	0.00
v29	0.00	0.00	0.01	0.00	0.00	0.00	0.00	0.00	-0.03	0.00	0.03	0.00	0.00	0.05	0.05	0.01	0.03	0.02	0.00	0.09	0.06	0.02	0.05	0.00	0.05	0.15	0.02	0.13	0.00	0.04	0.15	-0.01	0.00	0.00	0.00	-0.01	0.00	0.00	0.00	0.02
v30	-0.04	0.01	0.01	-0.04	0.00	-0.01	0.00	0.02	0.00	0.00	0.06	0.00	0.00	0.07	0.09	0.04	0.00	0.36	0.00	-0.03	0.07	0.07	0.01	0.00	0.01	0.05	0.03	0.02	0.04	0.00	0.03	-0.02	0.00	0.03	-0.01	-0.04	-0.03	0.00	-0.02	0.00
v31	0.00	0.00	0.00	0.00	0.01	0.00	0.02	0.01	0.00	0.01	-0.01	0.04	0.00	0.03	0.00	0.00	0.06	0.02	0.07	0.05	0.03	0.01	0.03	0.03	0.02	0.06	0.04	0.00	0.15	0.03	0.00	0.00	0.00	0.00	0.00	0.01	0.00	0.00	0.00	0.06
v32	0.00	0.03	0.03	0.01	0.00	0.01	0.00	0.00	0.00	0.00	0.01	0.00	-0.01	0.01	0.00	-0.02	0.00	0.00	0.00	0.00	0.01	-0.05	0.00	0.00	0.00	0.04	-0.15	-0.01	-0.01	-0.02	0.00	0.00	0.13	0.00	-0.11	0.03	0.00	0.00	0.06	-0.02
v33	0.00	-0.15	0.01	0.01	0.00	0.01	0.00	-0.01	0.00	0.00	0.00	0.01	-0.02	0.00	0.00	0.00	0.00	0.03	0.00	0.00	-0.01	-0.04	0.00	0.00	-0.02	0.00	0.00	-0.02	0.00	0.00	0.00	0.13	0.00	-0.01	-0.04	0.00	-0.03	-0.02	0.00	0.00
v34	0.02	0.00	0.00	0.00	0.09	0.00	0.00	0.00	0.00	-0.01	0.09	0.00	0.01	0.00	0.01	0.00	0.00	-0.03	0.22	0.00	0.00	0.02	0.00	-0.01	0.05	-0.01	0.00	0.00	0.00	0.03	0.00	0.00	-0.01	0.00	0.02	0.01	0.00	0.00	0.00	0.03
v35	0.00	0.01	-0.02	0.00	0.00	-0.01	-0.04	-0.01	0.00	-0.02	0.04	-0.02	0.16	-0.03	0.06	0.00	0.00	0.09	0.00	0.00	0.00	0.14	0.00	-0.03	0.00	-0.03	0.17	0.00	0.00	-0.01	0.00	-0.11	-0.04	0.02	0.00	-0.06	-0.05	-0.03	-0.03	0.10
v36	0.10	-0.01	0.06	-0.03	-0.02	0.11	0.00	0.00	0.00	0.00	-0.03	0.00	-0.02	0.00	-0.06	0.02	0.00	0.03	0.00	0.00	0.00	-0.02	-0.01	0.00	-0.01	0.00	0.05	0.00	-0.01	-0.04	0.01	0.03	0.00	0.01	-0.06	0.00	0.20	0.19	0.15	0.24
v37	0.00	0.00	-0.04	0.05	-0.06	0.06	0.05	0.02	0.00	0.01	-0.04	0.00	0.06	0.00	0.00	-0.07	0.01	0.00	0.00	0.01	0.09	0.00	0.00	0.10	-0.01	0.00	0.03	0.04	0.00	-0.03	0.00	0.00	-0.03	0.00	-0.05	0.20	0.00	0.04	0.16	0.00
v38	0.00	0.00	0.00	0.00	-0.02	0.00	0.00	0.01	0.00	0.11	-0.04	0.00	0.00	0.00	0.01	0.00	0.01	-0.01	0.00	0.02	0.00	0.00	0.00	0.03	0.03	0.01	0.00	0.02	0.00	0.00	0.00	0.00	-0.02	0.00	-0.03	0.19	0.04	0.00	0.11	0.33
v39	0.00	-0.04	0.00	-0.01	-0.02	0.04	0.03	0.01	0.00	0.00	0.00	-0.01	0.06	0.10	-0.06	-0.10	0.00	0.00	0.00	0.06	0.00	-0.01	0.00	0.00	0.00	0.05	-0.31	0.09	0.00	-0.02	0.00	0.06	0.00	0.00	-0.03	0.15	0.16	0.11	0.00	0.10
v40	0.05	0.00	0.00	0.00	-0.01	0.00	0.00	0.00	0.00	-0.02	0.01	0.00	0.07	0.00	0.03	0.01	0.00	0.02	0.00	0.03	0.00	0.01	-0.01	0.00	0.06	0.00	0.08	0.00	0.02	0.00	0.06	-0.02	0.00	0.03	0.10	0.24	0.00	0.33	0.10	0.00
